# Handling variability and incompleteness of biological data by flexible nets: a case study for Wilson disease

**DOI:** 10.1038/s41540-017-0044-x

**Published:** 2018-01-11

**Authors:** Jorge Júlvez, Duygu Dikicioglu, Stephen G. Oliver

**Affiliations:** 1 Cambridge Systems Biology Centre, University of Cambridge, Cambridge, UK; 2 Department of Biochemistry, University of Cambridge, Cambridge, UK; 3 Department of Computer Science and Systems Engineering, University of Zaragoza, Zaragoza, Spain; 4 Department of Chemical Engineering and Biotechnology, University of Cambridge, Cambridge, UK

## Abstract

Mathematical models that combine predictive accuracy with explanatory power are central to the progress of systems and synthetic biology, but the heterogeneity and incompleteness of biological data impede our ability to construct such models. Furthermore, the robustness displayed by many biological systems means that they have the flexibility to operate under a range of physiological conditions and this is difficult for many modeling formalisms to handle. Flexible nets (FNs) address these challenges and represent a paradigm shift in model-based analysis of biological systems. FNs can: (i) handle uncertainties, ranges and missing information in concentrations, stoichiometry, network topology, and transition rates without having to resort to statistical approaches; (ii) accommodate different types of data in a unified model that integrates various cellular mechanisms; and (iii) be employed for system optimization and model predictive control. We present FNs and illustrate their capabilities by modeling a well-established system, the dynamics of glucose consumption by a microbial population. We further demonstrate the ability of FNs to take control actions in response to genetic or metabolic perturbations. Having bench-marked the system, we then construct the first quantitative model for Wilson disease—a rare genetic disorder that impairs copper utilization in the liver. We used this model to investigate the feasibility of using vitamin E supplementation therapy for symptomatic improvement. Our results indicate that hepatocytic inflammation caused by copper accumulation was not aggravated by limitations on endogenous antioxidant supplies, which means that treating patients with antioxidants is unlikely to be effective.

## Flexible Nets: A modeling formalism for systems biology

In order to study complex dynamical systems, appropriate mathematical models that capture the system features are necessary. Biological systems, in particular, require flexible modeling approaches for their study since they exhibit variable quantifiable responses under different conditions. Moreover, data about a given biological system are often uncertain or unavailable. Here, a group of scientists from the University of Cambridge introduce Flexible Nets (FNs), a novel approach for the modeling, analysis, and control of biological systems. After presenting the FN approach, they show how a well-known system of glucose consumption and utilization by yeast can be modeled, analyzed and controlled. Then, FNs are used to build and analyze the first quantitative and predictive model of Wilson disease (a heritable defect in copper utilization). They demonstrate that FN simulations permit an evaluation of the relative efficacy of different therapeutic options.

## Introduction

In silico mathematical models are valuable tools in biological research since they can not only deepen our understanding of biological systems, especially when data availability is a limiting factor, but also reduce the time and costs associated with experiments through predicting their outcomes. These goals can only be achieved if suitable models and methods to facilitate the design, analysis, and optimization of biological systems are available. Model construction relies on the existence of formalisms that determine how models can be built. Computational biomodeling has always borrowed modeling formalisms from other disciplines to enable model construction and analysis. Some of the most widely used formalisms in biology include, but are not limited to, Boolean networks,^1–3^ Bayesian networks,^4,5^ cellular automata,^6,7^ constraint-based models,^8,9^ ordinary differential equations (ODEs),^10–12^ Petri nets^13–16^ and process algebras.^17–19^ Although these formalisms have been used in biology and have achieved various degrees of success, they do not address all of the difficulties presented by biological systems.

The quantity and quality of available biological data present serious challenges for the construction of models for biological systems. Despite the so-called “avalanche” of biological data, many data sets are necessarily incomplete. Moreover, there is a shortage of particular classes of data, e.g., the biochemical composition of different cell types, the kinetic constants of enzymes, or the structure of rate equations. Furthermore, models must be able to seamlessly accommodate, at various levels, heterogeneous data related to network topology, stoichiometry, rates of reactions, and the regulation of biological events. Conversely, models can also suffer from the availability of too much data since some biological systems have been investigated repeatedly, with only slight modifications, generating a range of allowable values that describe essentially similar situations. In addition, if the number of available data sets is not sufficiently large to permit the application of probabilistic approaches, models often suffer from either a form of averaging, which eliminates variability, or from the cherry-picking of data, which can introduce bias into the model’s predictions.

Here, we present a novel framework, which we call Flexible Nets (FNs), that specifically targets the modeling and optimization challenges presented by a wide range of biological systems. FNs can easily integrate heterogeneous data into a single model, and can cope with missing or varying information related to concentrations, stoichiometry, network topology, rates of reactions, and regulation of biological systems. Of the most popular modeling approaches used in systems biology, only constraint-based models can handle uncertainties in the flows of reactions. However, such models cannot accommodate concentrations, and their analysis is usually limited to the steady state. In contrast to constraint-based models, ODEs are deterministic descriptions of the system dynamics that can provide a precise time trajectory of the system. However, ODEs cannot account for the different system trajectories that arise as a result of uncertain parameters, moreover, the control of non-linear ODEs is a challenging problem. A major advantage of FNs with respect to these formalisms lies in its ability to track the evolution of concentrations in time-course analyses while handling uncertain kinetic and stoichiometric parameters. Moreover, the guarded FNs introduced here are an appealing alternative to control non-linear systems by considering a piecewise linear approximation of the system dynamics.

While FNs have been inspired by Petri nets, these two formalisms differ in a number of basic aspects. From a structural perspective, Petri nets are bipartite directed graphs whose nodes are places and transitions. In contrast, FNs consist of two nets: the event net and the intensity net. Each of these nets is a tripartite graph. The nodes of the event net are places, transitions, and event handlers; those of the intensity net are places, transitions, and intensity handlers. These nodes are connected both by directed and undirected edges (in the following, directed edges will be referred as “arcs”, and undirected edges as “edges”). The event net is used to model the stoichiometric relationships between reactants and products. For instance, an event net can be used to represent metabolic pathways by representing graphically the amounts of metabolites that are consumed and produced by the reactions involved in the pathways. The event net does not specify any rates for the modeled reactions. Such rates are expressed graphically by an intensity net that is connected to the event net. For instance, the reaction rates of the mentioned metabolic pathways can be specified by means of an intensity net. Each handler is associated with a set of inequalities that establishes the stoichiometric relationships in the event net, and the speed of reactions in the intensity net. Such inequalities can be used to model system uncertainties. For example, by associating inequalities with event handlers, different states are possible in FNs after the occurrence of an event (in contrast to Petri nets where the resulting state after the firing of a transition is deterministic). The association of inequalities with intensity handlers enables a range of reaction rates to be assigned to a given state. Moreover, the addition of guards to an intensity net offers the possibility of modeling the complex and non-linear dynamics of biological systems. All the potential evolutions of an FN arising from the existing uncertainties are accounted for by state equations that contain matrix inequalities.

In addition to the FNs modeling formalism, we have also designed the relevant methods to monitor the evolution of FNs over time. As FNs can handle initial concentrations, stoichiometry, reaction rates, and the regulation of reactions as variables, it is straightforward to derive methods to control and optimize biological processes. Note that FNs represent an abstract formalism and, as such, can be used straightforwardly to model dynamical systems in a wide range of different application domains, e.g., manufacturing, logistics, computer networks, traffic systems, etc. Hence their applicability goes far beyond biological systems (see section FNs for different interpretations of a net that originally modeled a biological system).

We demonstrate the capabilities of FNs using a widely studied biological process as a test object: the uptake of glucose and its subsequent utilization in yeast, which we call glucose consumption hereafter. Having bench-marked the methodology against this well-studied system, we then constructed the first computable model for Wilson disease. This model can account for the range of healthy or sick states documented, and can also cope with the missing information on the mechanistic details of the disease. The predictions made by analyzing this model have allowed us to investigate the utility of employing exogenous antioxidant supplements to alleviate the inflammatory response in the hepatocytes that occurs during copper accumulation. The model also suggests an alternative way to monitor the impact of a therapy on the level of copper stores in the liver without having to resort to invasive techniques, such as a liver biopsy.

## Results

### FNs overview

**Fig.1:**
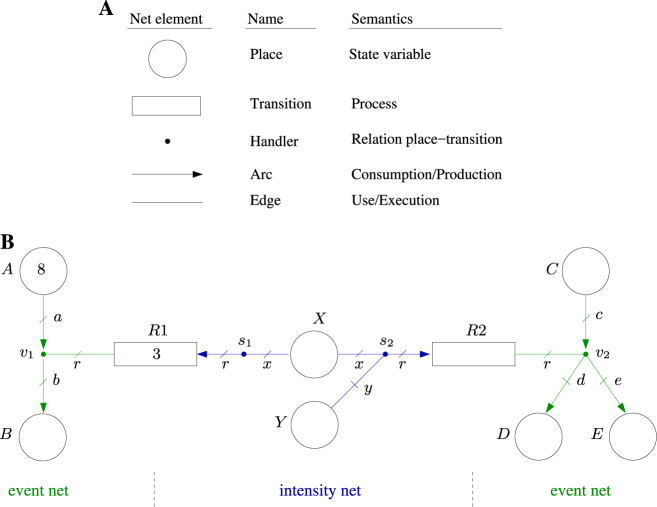
Constitutive elements of an FN and FN example. **a** Handlers provide a “flexible” layer between places and transitions. In the event net, places are connected to event handlers through arcs, and transitions are connected to event handlers through edges. In the intensity net, transitions are connected to intensity handlers through arcs, and places are connected to intensity handlers through edges. **b** FN modeling a system with two reactions, *R*1: *A* →* B* and *R*2: *C* → *D *+* E*, that are regulated by the molecules *X* (e.g., an enzyme) and *Y* (e.g., an activator). An FN consists of an event net (green elements) and an intensity net (blue elements) that share places and transitions. While the event net models how the reactions determine the changes in the state, the intensity net models how the state determines the rates of the reactions. The potential evolutions of the FN depend on the inequalities associated with handlers

A biological system can be described by its “state”, which represents the quantity of each of the species considered in the system at a given point in time, and by a set of “processes” (or events) specifying how the state changes over time. While the state refers to tangible components, e.g., numbers or concentration of molecules of each species, processes refer to any reaction, transformation, or translocation that produces a state change. The state of an FN can be graphically depicted by “places” and the processes by “transitions” (see Fig. 1a for an overview of the graphical representation and semantics of the net elements). FNs provide explicit and formal means to model the following two sorts of relationships between states and processes: (a) the processes determine how the state changes; (b) the state determines the rate at which the processes are carried out. Such relationships are established by sets of linear inequalities associated with a net element, called “handler”, that connects places and transitions. Linear inequalities make it easy for the practitioner to introduce uncertainties into the model. Thus, an FN consists of two subnets: an event net and an intensity net. The event net models how the processes determine the changes in the state; the intensity net models how the state determines the rates of the processes.

### Event net

In the following, the FN in Fig. 1b is used to introduce the basic features and modeling capabilities of FNs. Consider a simple biological system consisting of two reactions *R*1 : *A* → *B* and *R*2 : *C *→ *D* + *E*. The stoichiometry of these reactions is modeled by the “event net” (see net elements in green). In the FN, each type of molecule (*A*, *B*, *C*, *D*, and *E*) is associated with a “place” which is depicted as a circle. The amount (number of molecules or concentration) of each type of molecule is called a “marking”. The marking of a given place *p* is denoted *m*[*p*] (the initial marking of *p* is denoted *m*_0_[*p*]). The marking of a place can be written inside the place, e.g., *m*[*A*] = 8 in Fig. 1b. The marking of a place *p* is also known as the number of tokens in *p*. FNs can accommodate uncertain initial markings by means of inequalities, e.g., one can specify that the initial marking of *C* can be any value between 10 and 20 by associating the inequality 10 ≤ *m*_0_[*C*] ≤ 20 with place *C*.

Similar to the way places model molecules, “transitions”, which are depicted as rectangles (see *R*1 and *R*2 in Fig. 1b), are used to model reactions. In FNs, the number of tokens consumed and produced by the occurrence of a reaction is determined by a net element called “event handler”, which is depicted as a dot (see *v*_1_ and *v*_2_ in Fig. 1b). Event handlers are connected to transitions by edges which are used to specify that the occurrence of a reaction will be used by the event handler to change the marking of the places involved in the reaction. Such places are connected to the event handlers by directed arcs. An arc from a place to an event handler means that the place is a reactant, i.e., its number of tokens will decrease when the reaction happens. Similarly, an arc from an event handler to a place means that the place is a product, i.e., its number of tokens will increase when the reaction happens. Each edge and arc is labeled to facilitate its identification (to avoid confusion, all the arcs and edges connected to a given handler must have different labels). The actual number of tokens consumed and produced by the occurrence of a reaction is derived from the set of inequalities that is associated with each event handler and that makes use of the labels of the arcs and edges connected to them.

**Fig. 2:**
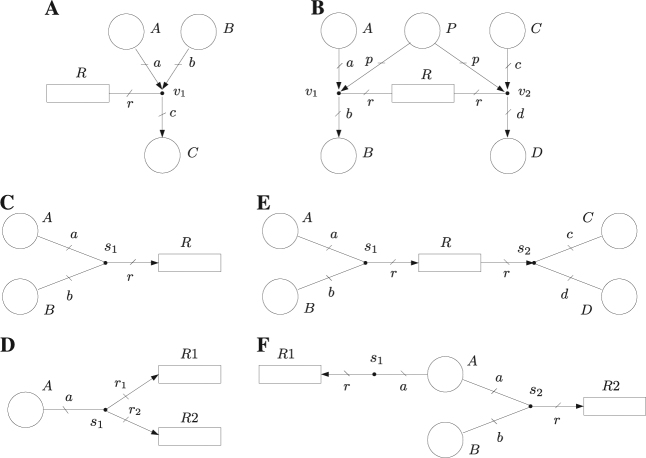
Event net examples (**a **and** b**) and intensity net examples (**c, d, e **and** f**). **a** Let us assume that the stoichiometry of a given reaction *R* is not completely known and can be expressed as *R*: *nA* + 2*nB* → *C* where *n* is known to be between 10 and 12. That is, the production of one molecule of type *C* requires a quantity of molecules of type *A* that is in [10,12] and twice as many molecules of type *B*. Such a reaction can be modeled by the event net in **A** together with the set of inequalities 10*r* ≤ *a* ≤ 12*r*;* b* = 2*a*; *c* = *r* associated with event handler *v*_1_. *R* can be expressed as *R*:*nA* + 2*nB* → *C* where *n* is known to be in [10,12]. The inequalities in *v*_1_ are interpreted as follows: If one reaction occurs, then *r* = 1 and one action in *R* is used to change the marking in places. In order to satisfy the inequalities, *a*, which is the number of tokens consumed from *A*, is between 10 and 12 (the actual value of *a* is chosen in a non-deterministic way for each occurrence of *R*), *b*, which is the number of tokens consumed from *B*, is *b* = 2*a*, and *c*, which is the number of tokens produced in *C*, is *c* = *r* = 1. Assume that the initial marking of places is *m*_0_[*A*] = 20, *m*_0_[*B*] = 22, *m*_0_[*C*] = 0. The occurrence of *R* drives the system non-deterministically either to *m*[*A*] = 10, *m*[*B*] = 2, *m*[*C*] = 1, or to *m*[*A*] = 9, *m*[*B*] = 0, *m*[*C*] = 1. Notice that, for this initial marking, *a* cannot take a value higher than 11 as it would lead to a negative *m*[*B*]. The consumption and production of tokens is instantaneous and coincident. **b** Net modeling a reaction *R* with two alternative set of reactants and products, the set of equalities associated with the event handlers are *v*_1_:*a* = *p* = *b* = *r*, *v*_2_:*c* = *p* = *d* = *r*; either *A* and *P* are reactants and *B* is product, or *C* and *P* are reactants and *D* is product. In a chemical context, this net might model a reaction *R* that either takes *A* and *P* as reactants and *B* as product, or *C* and *P* as reactants and *D* as product, i.e., *R* is either *A* + *P* → *B* or *C* + *P* → *D*. In the first case, the occurrence of the reaction, i.e., the action in *R*, is implemented by the edge connected to *v*_1_, and in the second case by the edge connected to *v*_2_. The selection of the event handler that uses the action is done non-deterministically for every occurrence of the reaction. This net can be interpreted in different ways: (a) The transition could model an event that can be observed, and whose occurrence can produce different marking changes which are modeled by the event handlers. For instance, the transition can model a biochemical event such as phosphorylation, but it is not possible for the observer to determine whether molecule *A* or *C* has been phosphorylated. (b) The transition could model an input action whose effect on the system is not fully controllable as any of the connected event handlers can make use of the reaction. **c** Net establishing a synchronization between the tokens in *A* and the tokens in *B* by means of the equation *s*_1_:2*a* = *b* = 2*r* associated with *s*_1_. More precisely, when a token in *A* synchronizes with two tokens of *B*, the intensity in transition *R* is increased by one unit. **d** Let *s*_1_:*a* = *r*_1_ = *r*_2_, then a token in *A* produces simultaneously an intensity unit both in *R*1 and *R*2, i.e., it can be informally said that the intensities of *R*1 and *R*2 are synchronized by the tokens in *A*. **e** Let *s*_1_:*a* = *b* = *r* and *s*_2_:*c* = *d* = *r*, then a token in *A* together with a token in *B* produce an increase of one unit in the intensity of *R*. Similarly, a token in *C* together with a token in *D* produce a decrease of one unit in the intensity of *R*. Thus, the tokens in *A* and *B* can be seen as positive modulators and the tokens in *C* and *D* as negative modulators. **f** Let *s*_1_:10*a* ≤ *r* ≤ 12*a* and *s*_2_:*a* = *b* = *r*. The net models a choice in place *A*, i.e., a token in *A* can be used either to produce an intensity within the interval [10,12] in *R*1 or, together with a token in *B*, an intensity in *R*2 of one unit. The choice of the intensity handler that uses the tokens in *A* is non-deterministic; in contrast to the actions used by event handlers, it can change over time. In other words, a given token in *A* can modulate the intensity in *R*1 during a given time period and then synchronize with a token in *B* to modulate intensity in *R*2

For instance, the labels *a*, *b* and *r* of the arcs and edge connected to event handler *v*_1_ denote the number of tokens consumed from *A*, the number of tokens produced in *B*, and the number of occurrences of reaction *R*1 respectively. Assume that the equations *a* = *b* = *r* are associated with *v*_1_. Such equations imply that the number of tokens consumed from *A* is equal to the number of tokens produced in *B*, and equal to the number of occurrences of *R*1. It follows that a single occurrence of reaction *R*1, i.e., *r* = 1, entails *a* = *b* = 1, i.e., it consumes one token from *A* and produces one token in *B*. In other words, the labels of arcs and edges are used as variables in the equations to specify the stoichiometric relations. Uncertain stoichiometric relations can easily be captured by inequalities. For instance, the association of *a* = *r*; 95*r* ≤ *b* ≤ 105*r* with *v*_1_ implies that a single occurrence of *R*1 consumes one token from *A*, and the number of tokens produced in *B* is selected nondeterministically in the interval [95,105]. Such a set of inequalities could be useful to model the decomposition of a large complex into an uncertain number of smaller components. As another example, the association of *r* = *c* = *d*; 12*r* ≤ *e* ≤ 14*r* with *v*_2_ would mean that each occurrence of reaction *R*2 consumes a token from *C*, produces a token in *D* and produces an uncertain number of tokens in the interval [12,14] in *E*. See Fig. 2a, b for other modeling capabilities of event nets.

### Intensity net

The “event net”, introduced above, models exclusively stoichiometric relationships. In order to model the system dynamics, i.e., the rate of the reactions represented by the transitions, the “intensity net” is introduced (see net elements in blue in Fig. 1b). The speed (or rate) of a transition *t* is called intensity, it is denoted *λ*[*t*] and it represents the number of times *t* occurs per time unit. The default intensity of *t* is denoted *λ*_0_[*t*], which is a default speed that can be increased or decreased by the “intensity net”. The default intensity of a transition *t*, *λ*_0_[*t*], can be written inside *t*, for instance the default intensity of *R*1 is *λ*_0_[*R*1] = 3. Similarly to the initial marking in an “event net”, uncertain default intensities can be incorporated by means of inequalities, e.g., 3.9 ≤ *λ*_0_[*R*2] ≤ 4.1 would mean that the default intensity of *R*2 is uncertain but within the interval [3.9,4.1]. Notice that if the speed of a transition *t* can be manipulated by an external controller, then *λ*_0_[*t*] can be interpreted as a control action that is applied to the system.

As in an event net, the key element of an intensity net is an “intensity handler”, which is depicted as a dot (see *s*_1_ and *s*_2_ in Fig. 1b). Places and transitions are connected to intensity handlers by edges and arcs respectively. A place connected to an intensity handler means that the tokens in that place are able to modify the speed of the transitions connected to the handler; an arc to/from the transitions implies that the speed will be increased/decreased by such tokens. Thus, in contrast to event nets, the tokens are neither consumed nor produced in the intensity net, just used to modulate the speed of transitions.

The speed of the transitions is determined by the set of inequalities associated with the intensity handlers. For instance, the intensity handler *s*_1_ in Fig. 1b is connected to place *X* (for instance, an enzyme) and transition *R*1; this means that the rate of *R*1 depends on the marking of *X*. The labels *r* and *x* of the arc and edge connected to *s*_1_ denote the increase of intensity in *R*1 produced by *s*1, and the number of tokens of *X* used by *s*_1_ to produce intensity in *R*1 respectively. If the equation *r* = 2*x* is associated with *s*_1_ then each token in *X* used by the intensity handler increases the rate of *R*1, which by default is *λ*_0_[*R*1] = 3, by two units. As an example, assume that the marking of *X* is 4, i.e., *m*[*X*] = 4, then the rate of *R*1 is *λ*[*R*1] = *λ*_0_[*R*1] + 2*m*[*X*] = 11 if *s*_1_ uses all the tokens in *X* to produce intensity in *R*1. Uncertain reaction rates can be modeled by inequalities, e.g., the inequalities 1.9*x* ≤ *r* ≤ 2.1*x* associated with *s*_1_ mean that each token increases the speed of *R*1 in a nondeterministic amount in the interval [1.9,2.1]. The fact that the speed of a reaction might depend on the coordinated activity of several molecules can be modeled by connecting several places to an intensity handler. Assume that the equations *r* = *x* = 0.5*y* are associated with *s*_2_. That would imply that one token in *X* must be associated with two tokens in *Y* (*Y* might represent an enzyme activator) to increase the speed of *R*2 by one unit. Note that a token in *A* can be used either to modulate the speed of *R*1 or *R*2, such a choice is nondeterministic.

The intensity nets in Fig. 2c–f show some of the basic modeling capabilities provided by the intensity handlers such as synchronization, choice, uncertainty handling, and interplay between positive and negative modulators. Notice that the nets in Fig. 2 can be seen as basic building blocks with which to construct larger and more complex nets.

### Flexible nets (FNs)

The combination of an event net and an intensity net results in an FN (see Fig. 1b). In an FN, the event net determines the way intensities produce marking changes, while the intensity net determines the way tokens produce intensity changes. Notice that FNs represent an abstract modeling formalism not necessarily constrained to biological systems. In fact, the FN in Fig. 1b admits interpretations from other application areas. For instance, from a manufacturing perspective *R*1 and *R*2 could model actions performed on two assembly lines, one line starting at *A* and the other at *C*. From this point of view, *X* could be a shared resource, e.g., a robot, required by both lines and *Y* a resource just required by one assembly line. From a computing point of view, *A* and *C* could be seen as data streams that need to be processed by server *X*, and by server *X* in cooperation with server *Y*, respectively.

Given that an FN can accommodate uncertain parameters, there is no unique possible trajectory for a modeled system, but a number of possible trajectories arising from the inequalities associated with initial markings, default intensities, event and intensity handlers. In order to account for all these possible trajectories, a set of linear and quadratic constraints has been developed (see S1). The values that satisfy these constraints are the possible states that the system can reach. In order to compute a state that maximizes a given property, an objective function can be added to these constraints; this results in a programming problem that can be solved by state-of-the-art solvers.^20,21^ Among the existing control possibilities, FNs are especially well suited for model predictive control.^22^ Under model predictive control, a number of time intervals (or sample times) are considered, and the programming problem described above yields the state of the FN, as well as the control action *λ*_0_, for each time interval. Such a control action is implemented during the first interval only, at the end of the first interval the state of the system is updated and the procedure is repeated. See S1 for the model predictive control approach in FNs.

**Fig. 3:**
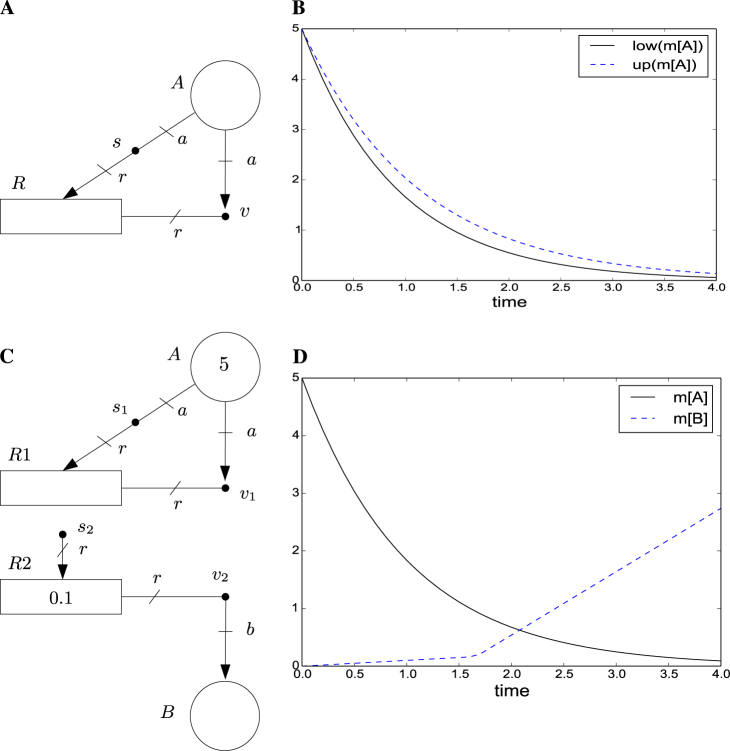
FN and guarded FN examples. **a** FN modeling an exponential decay with uncertain rate. Let *v*:*a* = *r* and *s*:0.9*a* ≤ *r* ≤ 1.1*a*, then the FN models the exponential decay of a molecule of type *A* whose exact rate is uncertain but known to be in the interval [0.9[*A*],1.1[*A*]], where [*A*] is the concentration of *A*. **b** Evolution of two objective functions with *m*_0_[*A*] = 5 and *λ*_0_[*R*] = 0. The dashed and the solid lines correspond to the maximization and the minimization of *m*[*A*], respectively. The trajectories provide an upper and a lower bound for the potential evolutions of *m*[*A*]. The plot has been obtained by model predictive control with sample time of 0.1 units and a prediction horizon of one step. **c** Guarded FN modeling an activation process. Let *v*_1_:*a* = *r*, *v*_2_:*b* = *r*, *s*_1_:*a* = *r* and *s*_2_:*r* = 0 if *m*[*A*]>1, *s*_2_:*r* = 1 otherwise. Notice that the equations associated with *s*_2_ depend on the state, more precisely on *m*[*A*]. Moreover, note that the tokens in *A* are not used to produce intensity in *R*2, and hence *A* is not connected to *s*_2_. The net models an exponential decay of molecule *A*, and a constant production rate of molecule *B* of 0.1 (this is modeled by *λ*_0_[*R*2] = 0.1) when *m*[*A*]>1 and of 1.1 when *m*[*A*] ≤ 1. Thus, *A* can be seen as a repressor that only allows a residual production of *B* when *m*[*A*] is higher than 1. There are only two regions in this net, one is defined as *m*[*A*]>1, the other as *m*[*A*] ≤ 1. **d** Marking evolution with *m*_0_[*A*] = 5, *m*_0_[*B*] = 0, *λ*_0_[*R*1] = 0, *λ*_0_[*R*2] = 0.1, and the objective function is to minimize *m*[*B*]. It can be seen that the linear growth of *m*[*B*] changes from rate 0.1 to 1.1 when *m*[*A*] falls below 1. The plot has been obtained through model predictive control with a sample time of 0.1 and a prediction horizon of one step

The FN in Fig. 3a models an exponential decay, e.g., the degradation of a molecule (the marking *m*[*A*] would be the number of molecules), with uncertain rate. Figure 3b shows two potential time trajectories of the FN, *low*(*m*[*A*]) and *up*(*m*[*A*]), that have been obtained by means of the mentioned programming problem. More precisely, once the linear and quadratic constraints are established, *low*(*m*[*A*])/*up*(*m*[*A*]) is obtained by an objective function that minimizes/maximizes *m*[*A*]. In this way, *low*(*m*[*A*]) and *up*(*m*[*A*]) are lower and upper bound trajectories that encompass all the possible time trajectories arising from the uncertain rate.

### Guarded FNs

Intensity nets can model intensity uncertainties by means of the linear inequalities associated with the intensity handlers. Although such linear inequalities can also be used to approximate non-linear dynamics, the resulting accuracy may not be satisfactory. In order to better approximate non-negative dynamics, guarded FNs are proposed.

In a guarded net, the set of potentially reachable markings is partitioned into a set of regions. Each region defines an “operation mode”, and intensity handlers are associated with a set of linear inequalities per region. The set of linear inequalities determining how intensities are produced is the one associated with the region where the marking lies. See Fig. 3c, d for a guarded net modeling the activation of production of molecule *B* when *m*[*A*] is below 1.

In order to account for guarded FNs, the programming problems developed for FNs were extended and the addition of binary variables was required. For the sake of simplicity, both FNs and guarded FNs will be referred to as FNs in what follows. Formal definitions of FNs and methods to analyze them through programming problems are provided in S1. We have developed a new software tool called *fnyzer* (see Code in SI) that implements these methods.

### Validation of the FN methodology: dynamics of glucose consumption by yeast population

As a proof-of-concept study, we modeled the uptake and consumption of glucose by yeast cells leading to cell duplication and consequent growth of the population. We mathematically described how these dynamic events are coordinated by different hexose transporters and glucose sensors as the availability of glucose varies over the course of time. We then studied situations in which we introduced perturbations to an ideal system and we describe control actions to overcome these perturbations so that the population could cope with changes in the physiological parameters, or with spontaneous mutations in the cells during cultivation. Although novel features, such as the modeling of the hierarchical coordination of dynamic metabolic events or the identification of control actions that need to be taken to meet given criteri,a can only be exploited by the FN methodology, we bench-marked our results against a parallel analysis that used ODEs whenever possible.

**Fig. 4:**
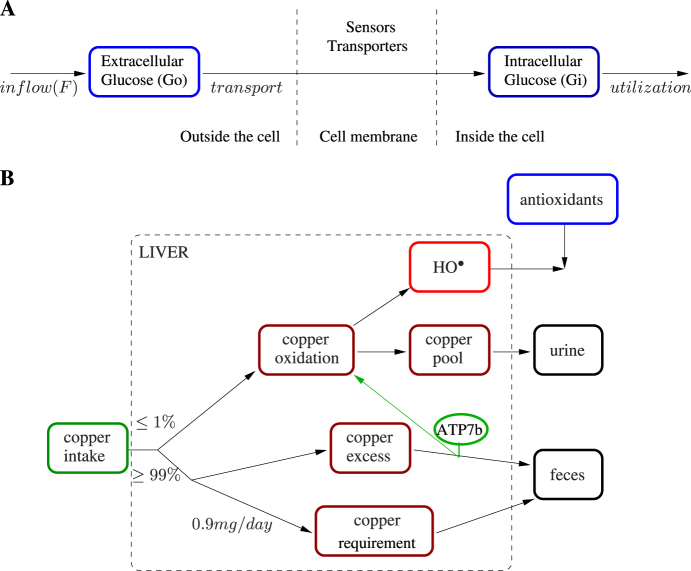
Main flows of the glucose consumption and Wilson disease models. **a** Sketch of the flows in the glucose consumption model. The model captures the amount of extracellular (left) and intracellular (right) glucose. An inflow (F) of glucose can be fed into the system, this flow will be determined by the solution of control methods. The extracellular glucose is sensed and transported into the cell, where it is utilized. **b** Sketch of the main flows in the Wilson disease model. The copper intake flow is initially split in two. One of the flows, accounting for at most 1% of the intake^33^ undergoes oxidation resulting in the production of hydroxyl radicals. These radicals can be neutralized by antioxidants. After oxidation, copper is excreted in the urine via the kidneys. The remainder of the intake flow, accounting for at least 99% of the intake, is split in two further flows. One of them is used to fulfill the body’s copper requirements, which are around 0.9 mg/day.^31^ The copper excess is not used and is removed through the feces. If ATP7b is non-functional, this impedes the normal excretion of the excess copper into the bile ducts and, as a result, some of it is oxidized, producing more hydroxyl radicals

We tracked the fate of an average individual cell from its inoculation into a glucose-rich batch culture and followed the trajectory of its uptake of glucose, growth, and its cell division until the carbon source available per average cell became scarce due to both consumption and the increase in the size of the population. In order to simulate a complete batch growth profile, we considered seven replication periods of 105 min each. We illustrate the approach as a black-box cell system where the glucose was transported and used for growth and maintenance. Figure 4a sketches the glucose flows in the modeled system (see S2 for details on the FN modeling the system and its parameters). The amount of extracellular glucose (Go) can be increased by means of an inflow (F) that represents a control action that can be applied into the system in order to control it. The model includes two sensors (Rgt2p and Snf3p) and nine tranporters (Hxt1p-7p, Hxt13p and Hxt17p). Each sensor and each transporter has an affinity profile based on Go. If at least one sensor and one transporter is active for the current amount of Go, then the transport rate is positive (see S2 for the rate equation), otherwise the transport rate becomes 0. The utilization rate of intracellular glucose (Gi) was set to 199*e*-6 mM/min to meet the glucose-equivalent energy required for maintenance^23^ normalized for a single yeast cell.

Three possible situations for the glucose consumption system were assessed. We first investigated how the wild-type yeast cell transported glucose across its membrane and utilized it for growth and maintenance. A cell division was assumed to take place at the end of each doubling period, approximated by the accumulation of intracellular glucose to double its initial content. Only half of the remaining amount of extracellular glucose was available for a single cell after each division.

**Fig. 5:**
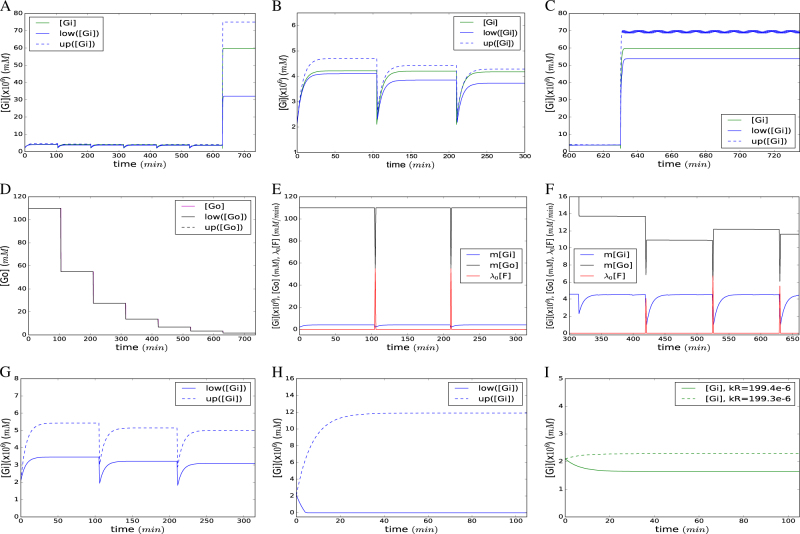
Analysis of the glucose consumption system by FNs. **a** Evolution of intracellular glucose for a wild type yeast cell configuration throughout the cultivation time. **b** A close-up trajectory for [*Gi*] during the initial 3 replication events. **c** Tighter upper and lower bounds achieved during the transition from the low-affinity to high-affinity regions by lowering the sample time to 0.1 min. In **a**–**c**, the blue solid line, blue dashed line, and the green solid line represent the lower bound, upper bound, and the ODE trajectories for [*Gi*], respectively. **d** Evolution of extracellular glucose for the wild type cell population throughout cultivation. The black solid line, black dashed line, and the magenta solid line represent the lower bound, upper bound, and the ODE trajectories for [*Go*], respectively. **e** Control action, *λ*_0_[*F*], to maintain a steady supply of [*Go*] at 110 mM. **f** Control action, *λ*_0_[*F*], taken to overcome the effect of *hxt4*Δ*hxt13*Δ*hxt17*Δ triple deletion on the cultivation. The control action was required at *t* = 420 min to keep [*Gi*]>1*e*-6 mM and minimize the amount of glucose introduced to the system. The obtained control law imposes an impulse of glucose *F* to return the system to a state with a non-zero transport rate. Similar impulses are required for subsequent cell divisions. In **e**–**f**, the black, blue and red solid lines represent the trajectories of *m*[*Go*], *m*[*Gi*], and *λ*_0_[*F*], respectively. **g** Upper-bound and lower-bound trajectories of [*Gi*] with uncertain utilization rate in the interval [198.9*e*-6,199.1*e*-6] mM/min. **h** Upper-bound and lower-bound bound trajectories of [*Gi*] with uncertain utilization rate in the interval [198.0*e*-6,200.0*e*-6] mM/min. In **g**–**h**, the solid and dashed lines correspond to the lower and upper bound trajectories of [*Gi*]. **i **Trajectories of [*Gi*] predicted by the ODE model with exact utilizaton rates of 199.4*e*-6 mM/min and 199.3*e*-6 mM/min. In **i**, the solid line corresponds to a utilization rate of 199.4*e*-6 mM/min, and the dashed line to a utilization rate of 199.3*e*-6 mM/min

The trajectories we obtained using the FN model for the evolution of intracellular and extracellular glucose concentrations as the latter become depleted were observed to follow a similar path to the trajectories obtained by the ODE models. This validated the operating principles of the methodology and were also congruent with empirical physiological observations on yeast cells (Fig. 5a–d).^24^ These trajectories were obtained by model predictive control^22^ with a sample time of 1.0 min and a prediction horizon of one step (according to this control approach, the programming problem derived from the FN model is optimized at each sample time, and the obtained solutions represent the values of the state variables that optimize the given objective function). A shift in intracellular dynamics was observed as the availability of extracellular glucose changed over time. The remarkable increase we observed in the rate of glucose uptake and its intracellular concentration upon depletion of the external resources was in line with earlier reports.^25^ The FN upper-bound and lower-bound trajectories for [*Gi*] and [*Go*] were shown to enclose the ODE trajectory throughout the time frame of analysis. The maximum deviation from the ODE prediction remained within 12% throughout the low-affinity regions (Fig. 5a, b) whereas the maximum deviation was as high has 95% in the high-affinity regions (Fig. 5a). Tighter bounds with the maximum deviation remaining within 17% were achieved with a shorter sample time of 0.1 min (zoomed in section in Fig. 5c) in the non-flat, high-affinity region of the graph. The maximum deviation between the upper-bound and lower-bound trajectories and the ODE trajectory for [*Go*] remained negligible throughout the time course of analysis (Fig. 5d). The substantial difference in the number of glucose molecules available in the cell as compared to that outside was the main reason for the differences observed in the deviation trends for [*Gi*] and [*Go*]. Lowering the sampling time provided an immediate solution to overcome this challenge.

Having bench-marked the formalism, we then exploited this system and demonstrated how FNs can be used to handle two very different control problems in biology. The first control problem focused on maintaining the physiological state of a microbial fermentation. The control goal was to compute the time trajectory of *λ*_0_[*F*], so that [*Go*] is maximized and constrained to [*Go*] ≤ 110 mM. The control action *λ*_0_[*F*] was obtained by optimizing a programming problem that had “max [*Go*]” as the objective function and was subject to the constraints associated with the FN model plus [*Go*] ≤ 110 mM. In this programming problem, *λ*_0_[*F*] was taken as a variable and the actual value of *λ*_0_[*F*] obtained by the optimization was the control action to be implemented in the system. The overall time trajectory of *λ*_0_[*F*] was obtained by a model predictive control approach, according to which the mentioned programming problem was solved at each sample time, and the control action was applied at each of the sample times. The computed trajectory suggested that a reasonably low and nearly constant supply of glucose (199*e*-6 mM/min) was required throughout the cultivation with impulses of glucose provided at the time of cell division (Fig. 5e).

As the second control problem, we studied the question of how to overcome the physiological problems arising in a microbial fermentation as a consequence of a genetic modification of the yeast. A deleterious genetic change (*hxt4*Δ*hxt13*Δ*hxt17*Δ) was considered in this situation. While this is a very extreme case that would never occur by spontaneous mutation, it presented a suitably tough challenge to use to test the control capabilities of FNs. The goal was to maintain the cell in a physiological state where these medium-affinity transporters (see S2) would not be required for survival or for growth, while the fermentation was still economically feasible. The control goal was to keep [*Gi*] ≥ 1*e*-6 mM and to minimize the amount of glucose put into the system. The control trajectory was obtained through the approach described in the previous control problem. No control action was required when the cultivation proceeded with the relevant transporters in action and [*Gi*] was above the set threshold. The cell division event at 420 min moved [*Go*] to a region where the system could not operate due to the absence of the relevant transporters (see region low_A in S2). The control law that was invoked to counteract the cessation of glucose uptake, which would in time lead to the depletion of [*Gi*], dictated the impulse-like supplementation of glucose (*λ*_0_[*F*]) to bring the system back to the region low_B (see S2). Similar impulses of glucose were required for the subsequent division events (Fig. 5f).

As a final validation exercise, we investigated the responsiveness of the system to the rate at which glucose is consumed for maintenance purposes. In order for the model to correctly represent yeast physiology, we expect the glucose-equivalent maintenance requirement, which is independent of the specific growth rate, and consequently of the availability of nutrient and energy resources ([*Go*]), by definition, to remain unchanged. Since the maintenance portion should represent the minimal survival requirement for the cell, the model was expected to be highly sensitive to even very small reductions in this value.^26^ To test this notion, we ran several simulations varying the rates of maintenance and monitored how [*Gi*] was affected during replication events. Confirming our predictions, the model only tolerated ±0.05% deviation from the empirical value (Fig. 5g) to maintain “survival”, and [*Gi*] was observed to be rapidly depleted within a single replication event, indicating inviability in the model even when this range was extended to ±0.5% (Fig. 5h). We then identified the break-even confidence interval for the maintenance requirement as ±0.15% as predicted by the ODE model (Fig. 5i).

The modeling and control of single-cell behavior, such as the problem discussed above, is of particular interest when implementing design and control strategies for microfluidic cell culture systems such as “lab-on-a-chip” devices. FNs can also be readily exploited to model and control systems of populations of asynchronously dividing cells, e.g., a population of cells in a fermenter at either the laboratory or industrial scale.

### Modeling of copper accumulation in Wilson disease and its treatment

Rare medical disorders present challenges that are associated with both the data and the available analytical tools. The low rate of incidence of such diseases hinders the collection of extensive data on both patients and disease mechanisms. The impracticability of gathering large patient cohorts makes it difficult or impossible to conduct extensive drug trials. The limited size of the target group is often a financial discouragement to the development of new treatments. All of this means that rare or neglected diseases are very likely to benefit from the application of model-based approaches, to accelerate both clinical and non-clinical research, since they can substantially reduce the time and costs associated with an in-depth investigation of a condition or the evaluation of alternative treatment strategies, shortening the path to success.^27^

Wilson disease is an autosomal recessive heritable disorder affecting an individual’s copper metabolism. The copper taken in with the diet and stored by the hepatocytes in the liver is transferred to a carrier protein called apoceruloplasmin (ACP) to form a six-copper binding protein known as ceruloplasmin (CP). CP is then released into the bloodstream, and is the source of copper for organs such as the brain and kidneys; any excess copper is excreted into the bile ducts. Both of these key functions in copper metabolism are mediated by the copper-transporting P-type ATPase; ATP7b.^28,29^ Mutations in the gene encoding ATP7b can result in the accumulation of copper deposits in the body leading to Wilson disease.

The disease is very rare and presents a multisystemic symptomatology with non-specific neurological, hepatic, psychiatric, or osseo-muscular manifestations (Orphanet; citing information at ref. ^30^). Although some progress in understanding disease mechanisms and treatment strategies has been made, a number of issues regarding its treatment remain open. Generally, the course of treatment involves reduction in the dietary intake of copper; inhibition of copper absorption; stimulation of the release of accumulated copper in various tissues; alleviation and, if possible, reversal of any possible organ and tissue damage observed at diagnosis. A lifelong regime of maintenance therapy is then prescribed to prevent the reaccumulation of copper. An alternative treatment regime, involving the use of antioxidants in the treatment has been reported in some studies and has been claimed to sometimes result in symptomatic improvement.^31^ Furthermore, although the extent of accumulation of copper in the liver is generally accepted as a clinical diagnostic measure, follow-up liver biopsies to monitor whether treatment results in reduced copper levels are not commonly performed. Instead, the progress of the treatment is usually monitored by taking blood samples and measuring 24-h urinary copper excretion rates. We set out to compare the likely efficacy of the different treatments by exploiting FN modeling.

The disease begins with liver dysfunction, with patients affected in their early years being likely to be affected by liver damage.^32^ whilst older victims tend to present with neurological symptoms. Therefore, we chose to model the hepatocytes, where the disease first manifests itself. We defined the model boundaries considering the liver as the system. First, we allowed the model to select a value for the daily intake of copper that is within the range recommended by the WHO; this value then determines the influx of copper to the system (CI). We allow 99% of this amount to be processed through the control of ATP7b. The copper required to be released into the bloodstream to be transported to different parts of the body, such as the brain and the kidneys, is transferred (at rate CR) onto ACPs and the holoceruloplasmins are then released into the bloodstream. Excess copper is guided to the biliary ducts (CFBD) in a process that is also under the control of ATP7b. One percent of the copper remains unbound and is readily oxidized in the presence of H_2_O_2_. The copper is then discharged from the liver and discarded by urinary excretion (CFUE). This oxidation reaction generates hydroxyl radicals that are neutralized by glutathione, the natural antioxidant found in the liver. A schematic representation of this model is provided in Fig. 4b (a detailed description of the FN model and its parameters can be found in the S3). The model parameters depend on the “case” or “state” under consideration. Here, we have considered four different states: healthy (no copper accumulation), sick (copper accumulation dependent on ATP7b functionality), copper absorption blocking treatment, and urinary excretion induction treatment.

In order to construct our model, we first used medical practice guidelines to constrain the system to represent the fully functional copper metabolism of healthy individuals. We identified the maximum average daily copper intake as 1.98 mg in order to avoid any accumulation of copper in the liver. We carried the analysis out for a period of 70 years to monitor how the system evolved throughout a realistic lifespan. The upper bound of the range we explored denoted the maximum tolerable limit for copper ingestion without causing toxicity (10 mg/day); therefore, the fact that the model predicted the maintenance of a healthy hepatic copper balance only when the average copper intake in the diet was considerably lower than this maximum tolerable limit is consistent with reported dietary guidelines. However, we should also note that this value represents the daily amount of copper that would be available in the hepatocytes for further processing. It assumes that the absorption of copper from the intestines, and its conveyance via Cmt1 and Atp7a into the system boundaries (which were not investigated within the scope of this analysis), were carried out with 100% efficiency. This source of underestimation should be taken into consideration in evaluating the model predictions.

**Fig. 6:**
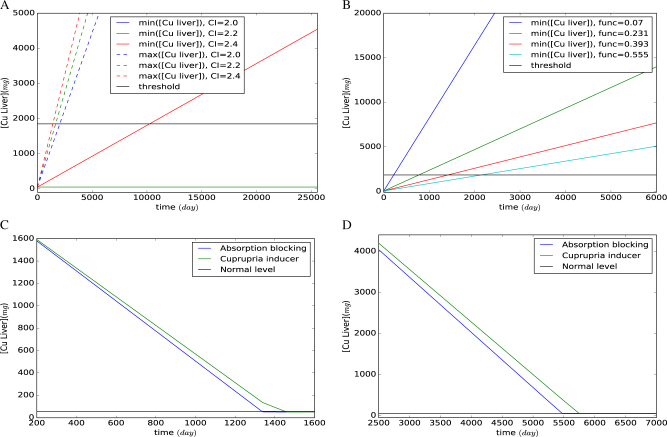
Copper accumulations predicted by the FN model (**a**–**b**) and performance of two treatments (**c-d**). **a** Maximum and minimum level of copper accumulations for different daily intakes. Red/green/blue lines refer to daily copper intakes of 2.0/2.2/2.4 mg. Maximum/minimum accumulations are represented with dotted/solid lines. The minimum accumulations for 2.0 mg/day and 2.2 mg/day copper intakes are constant and have the same profile. The black line at 1850 mg represents the diagnostic threshold. **b** Minimum copper accumulations for different levels of ATP7b enzyme activity. The cyan/red/green/blue lines correspond to 7%/23.1%/39.3%/55.5% of wild-type activity. The black line at 1850 mg represents the diagnostic threshold. **c–d** Profiles of the accumulation of copper during treatments to either block its absorption or induce its excretion (also known as cuprupria induction), with initial copper concentrations of 1850 mg in C and 7400 mg in **D**. The black line represents a normal copper level in a healthy individual

We also investigated the fate of hepatic copper for various daily copper intakes that were representative of different diets. We determined the maximum and minimum level of accumulated copper that could be caused by these different diets (Fig. 6a). Maximizing the rate of the accumulation of copper in the liver rapidly caused an excess accumulation of the metal, irrespective of the copper intake value chosen within the defined range However, since living systems operate to maintain a steady state as long as possible, it is not unlikely that the cellular objectives coincided with the minimization of the hepatic accumulation of copper in this in silico analysis. The hepatic accumulation was not completely unavoidable even when the intake rate was minimized. However, for a daily intake of 2.23 mg of copper into the liver, the accumulation throughout life was not so high as to exceed the limits provided in the medical guidelines for disease diagnosis (1850 mg equivalent to ≥ 250 μg/g* dry weight*).

The model was then used to investigate Wilson disease with various degrees of loss of ATP7b enzyme activity (Fig. 6b). We constrained the model to minimize the accumulation of copper in the liver since the robustness of the living system will act to minimize the effect of ATP7b dysfunction. We observed that proportional activities (compared to wild type) above 0.7 did not cause any accumulation of copper in the liver with the given constraints and the adopted objective. This was in line with earlier reports on the genotype–phenotype relationships in Wilson disease investigating the loss of ATP7b activity in mutants having both a pathogenic or a neutral phenotype where any mutant with a loss in enzyme activity less than 30% maintained normal copper transport capability^33^ and thus was considered neutral in its phenotypic effect. We investigated a relatively severe case of a 44% loss in ATP7b activity and determined that the hepatic copper accumulation exceeds the diagnostic limit of 1850 mg at the age of 28 (Fig. 6b). The model successfully predicted cases, where as much as 93% of the activity of the adenosine triphosphatase 7 was lost, leading to sufficiently high hepatic copper accumulation to allow definitive diagnosis within the first year of life (blue line in Fig. 6b). Any further loss in enzyme activity did not yield feasible predictions within the constraints used in the model, although human mutants leading to even higher losses in enzyme activity have been reported.^33^ However, cases with extremely early onset of the disease^34,35^ caused by mutations leading to a severe loss of ATP7b did not always present in a manner that complied with the constraints we had imposed on system, based on the published practitioners’ guidelines.^31^ Such an example was observed for the case of a 9-month-old patient with no change in urinary copper concentration despite a high hepatic copper accumulation (*ca*. 5550 mg) and high free serum copper concentration.^35^ The inability of the constrained model to account for these extreme cases may indicate the existence of as yet undiscovered mechanisms involved in the very early onset of the disease.

Having established the “sick” states in silico, we then investigated two alternative treatment strategies. The first involves blocking the intestinal absorption of copper and limiting the copper available for transport into the liver. This strategy will be referred as “copper absorption blocking”. Since normal bodily functions still demand a fixed amount of copper, insufficient uptake of copper creates a copper deficit that the body meets by calling on the copper accumulated in the liver, thus gradually relieving the diseased condition. The other treatment strategy involves the drug-induced release of hepatic copper stores by directing copper towards urinary excretion. This second strategy will be called “urinary excretion induction", or "cupruria-inducing treatment”. Both these routes are employed in the clinic in a two-stage therapeutic regime. The first stage is the *acute* or symptomatic treatment that aims at depleting the accumulated copper; the second stage is the "maintenance" treatment that consists of a 25% reduction in treatment dosages for lifelong maintenance therapy.

We focused on the severe loss of 93% of ATP7b enzyme activity and investigated the effect of early detection on the progress of both treatments, and how the length of the acute treatment varied in the two treatment regimes. The model was used to predict the copper depletion profiles from two different initial levels of copper accumulation: (a) the lower diagnostic limit for hepatic copper accumulation adopted in liver biopsies (1850 mg) (Fig. 6c) and (b) four times this lower limit as the upper bound (7400 mg) (Fig. 6d) to account for diagnosis at a much later stage.^36^ We monitored the depletion of the hepatic copper stores during treatment as a measure of recovery. We found that, although the two treatment strategies have different mechanisms of action, the time of response to either treatment was predicted to be similar. For a model of the hepatic copper accumulation of 1850 and 7400 mg at the time of diagnosis, the absorption blocking treatment could deplete copper stores in 1335 days and 5470 days, respectively; whereas these values were determined as 1450 days and 5744 days in the case of modeling cuprupria-inducing treatment.

We also computed the maximum allowable copper intake during the maintenance treatment in order to ensure that hepatic copper levels remain within healthy bounds. For the absorption blocking treatment, this maximum intake computed by the FN model was 1.34 mg/day, and for the urinary excretion induction treatment 1.67 mg/day.

The accumulation of copper was reported to trigger inflammation in the liver since the copper readily oxidizes generating hydroxyl radicals.^37^ As a final query, we investigated the potential for using exogenous antioxidant supplements to relieve such inflammation. Supplementation of the diet with alpha-tocopherol (vitamin E) has been suggested as a possible route to provide symptomatic improvement for individuals suffering from Wilson disease.^31^ Our results indicated that the endogenous supply of glutathione was sufficient to address the burden of hydroxyl radicals formed by the oxidation of the copper accumulating in the liver. This suggests dietary supplementation with antioxidants is unlikely to provide any additional benefits to the established two-phase therapy.

## Discussion

We have developed and implemented FNs, as a formal framework for the modeling, analysis, and control of complex dynamic systems. FNs were inspired by Petri nets and are useful for intuitively modeling the relationships between the state of the system and the processes altering it. FNs are able to approximate non-linear dynamics, and handle uncertain parameters without relying on statistical methods. This framework should be especially valuable to meet the modeling demands of biological systems and to overcome the challenges associated with the use of the available biological data. The strength of the FN approach, as demonstrated by the design and control of the model of glucose consumption, lies in its ability to incorporate highly heterogeneous and imprecise data but, nevertheless, provide predictive models of biological systems and predictive control of bioprocesses. FNs thus fill gaps in the utility of existing formalisms and provide an important new tool that may be exploited by both research scientists and bioprocess engineers.

In this paper, we have demonstrated that predictive mathematical models constructed and analyzed using the FN formalism, which has been specifically designed to meet the demands of biological systems, can make a significant contribution to our understanding, especially when only limited information on the system is available. FNs have allowed us to integrate and exploit uncertain biological data by providing a unified formal model that is suitable for mathematical analysis. In particular, FNs have proven their usefulness in the investigation of the physiological changes that occur in patients suffering from, or being treated for, Wilson disease. We determined that the body’s endogenous antioxidant supplies were sufficient to meet the production of additional hydroxyl radicals caused by the accumulation of copper in the liver. This allowed us to conclude that the inflammation in the liver cannot be entirely due to the formation of hydroxyl radicals and that other, indirect routes must be involved in the induction of oxidative stress. Furthermore, we identified the trajectory for how the level of copper accumulated in the liver could be reduced by treatment. This model can correctly suggest a projection of how the treatment will alter the copper content of the liver, without having to resort to invasive methods.

## Methods

The inequalities associated with the handlers make the evolution of FNs non-deterministic (note that deterministic behaviors can also be modeled with appropriate inequalities). In order to cope with such non-determinism, a set of mathematical approaches that constrain the potential markings that the net can reach has been developed. Namely, approaches to constrain the following set of markings are available: a) markings that are reachable just using the event net (this ignores the intensity net and corresponds to an untimed interpretation); b) markings that are reachable after *τ* time units, where *τ* is a positive real number (this corresponds to a transient state analysis).

The obtained constraints represent necessary conditions for reachability. The tightness of the constraints depends on the net structure and on *τ* for transient analyses (generally the lower the value of *τ*, the tighter the constraints). In order to obtain a trajectory of the marking over time, intermediate sampling instants (not necessarily evenly distributed) can be introduced. At each sampling time, the marking is a variable within a bounded set and time-dependent parameters can be modeled. This usually involves using a high number of inequalities to define the constraints. To alleviate this complexity, model predictive control^22^ approaches have been implemented. Such approaches can deal with sample times of different lengths and can reset the marking when a given condition is satisfied.

Once the constraints of an FN are obtained, objective functions of interest can be defined to analyze the system. This results in an optimization problem whose solution represents a bound for all the potential system behaviors (see S1 for details). In FNs, both the initial marking, *m*_0_, and the default intensities, *λ*_0_, can be taken as variables whose values must satisfy given sets of linear inequalities. In this way, *m*_0_ and *λ*_0_ become variables of the problem to be optimized. Once the problem is solved, the values assigned to *m*_0_ and *λ*_0_ can be implemented in the real system to optimize the defined objective function. Thus, both analysis and control are carried out in a similar way in FNs, the only difference being that *m*_0_ and *λ*_0_ are taken as control variables when control is considered.

The net models, together with the analysis and control methods, have been implemented in Python 2.7 (https://www.python.org). The software makes use of Pyomo^38,39^ to build the optimization problems and Gurobi^21^ and CPLEX^20^ to solve them.

The performance of FNs remained within reasonable limits of CPU time (Intel i7, 2.00 GHz, 8 GiB, Ubuntu 14.04 LTS). Namely, the CPU time to compute one step in the glucose consumption model was 5.96 s for the wild-type cell and 44.36 s for the mutant with the state space partitioned in four and ten regions, respectively. With respect to the Wilson disease model, the profiles were obtained by optimizing the transient state of the FN over: (a) 10 steps of 7 years for the healthy and sick states (CPU time: 12.14 s); (b) 12 steps of 4 months for the acute treatments starting at a hepatic copper content of 1850 mg (CPU time: 34.47 s); (c) 24 steps of 9 months for the acute treatments starting at a hepatic copper content of 7400 mg (CPU time: 98.04 s); and (d) 4 steps 5 years for the maintenance treatments (CPU time: 4.44 s). The dense sampling in the acute treatments allowed us to reveal the slight differences in their profiles (Fig. 6c, d).

### Supplementary information

**S1**: Formal definition of FNs and methods to analyze them (PDF).

**S2**: FN modeling the glucose consumption by yeast population (PDF).

**S3**: FN modeling the Wilson disease (PDF).

### Data availability

Code: The code of the software tool fnyzer to analyze and control FNs is available at https://bitbucket.org/Julvez/fnyzer.git (see file nets/npjsba2017.txt for the FNs and parameters used in this paper).

## Electronic supplementary material

Supplemental File S1

Supplemental File S2

Supplemental File S3

## Electronic supplementary material

**Supplementary Information** accompanies the paper on the *npj Systems Biology and Applications* website (10.1038/s41540-017-0044-x).
