# Supplementary material for: Handling variability and incompleteness of biological data by flexible nets: a case study for Wilson disease
Source: NPJ Syst Biol Appl. 2018 Jan 11;4:7. doi: 10.1038/s41540-017-0044-x (PMC5765040; doi:10.1038/s41540-017-0044-x)
Supplement: Supplementary file 1 — Supplemental File S1 [file 41540_2017_44_MOESM1_ESM.pdf]

Supplementary Information

Flexible Nets: Definition and Bounds

This document provides formal definitions for flexible nets and methods to analyze them.

Contents

1 Flexible net. Definition **1**

2 Flexible net. Bounds **17**

3 Guarded flexible net. Definition **23**

4 Guarded flexible net. Bounds **30**

5 Intermediate states **35**

6 Optimization and control **36**

A Intensity bounds **37**

B Relaxing non linear constraints **41**

C Auxiliary bounds **43**

1 Flexible net. Definition

A flexible net is composed of an event net together with an intensity net sharing the same set of places and transitions. The intensity net establishes the intensities, rates or speeds at which events are produced. The event net handles these events and updates the net state. Thus, flexible nets explicitly uncouple the net elements representing the relationships between consumption and production of tokens (event handlers and arcs connected to them) from the net elements involved in the timing of the model (intensity handlers and arcs connected to them).

1.1 Event net

This subsection describes event nets, which can be denoted as $T/V/P$ nets, i.e., the actions in transitions T produce markings in places P through event handlers V. The semantics of places and transitions is inherited from Petri nets (see [4] for a gentle introduction). However, the introduction of event handlers to connect those elements significantly enriches the modeling power of the formalism, and produces the following main differences with respect to Petri nets: a) the marking changes are carried out by a net element called *event handler*; b) the marking change produced by the *firing* of an event handler is allowed to be nondeterministic; c) transitions can synchronize through event handlers to perform a join work.

1.1.1 Net structure

Definition 1 (Event net) *An event net is a tuple* $\mathcal{N}_V = (P, T, V, E_V, A, B)$ *where* (P, T, V, E_V) *is a tripartite graph determining the net structure and* (A, B) *are matrices determining the potential evolutions of the model.*

The set of vertices of the net is partitioned into three sets:

- $P = \{p_1, \ldots, p_i, \ldots\}$ is a set of $|P|$ places.
- $T = \{t_1, \ldots, t_j, \ldots\}$ is a set of $|T|$ transitions.
- $V = \{v_1, \ldots, v_k, \ldots\}$ is a set of $|V|$ event handlers.

The places, which are depicted as circles, model the different types of components or elements of the system, e.g., resources, products, items, molecules, energy, servers, machines, processors, etc. The transitions, which are depicted as rectangles, model the different types of operations, activities or processes of the system. Such operations require time to be performed and have the potential to change, i.e., produce and/or consume, the amounts of components of the system. The event handlers, which are depicted as dots, model the different ways in which the operations can change the amounts of components.

The vertices of the net are connected by the edges in E_V. Each pair of vertices can be connected by at most one edge. The set E_V is partitioned into two sets E_V^P and E_V^T, where E_V^P is a set of directed edges connecting places to event handlers and vice versa, and E_V^T is a set of undirected edges connecting transitions and event handlers. For simplicity, directed edges will be referred as arcs, and undirected edges as edges. More formally:

- Every $e \in E_V^P$ is either an arc $e = (p_i, v_k)$ from a place p_i to a handler v_k, or an arc $e = (v_k, p_i)$ from a handler v_k to a place p_i.
- Every $e \in E_V^T$ is an edge $e = \{t_j, v_k\}$ connecting a transition t_j and a handler v_k.

Notice that connections among places and transitions are not allowed. The following notation will be used:

- ${}^p v_k$ denotes the input places of v_k, i.e., ${}^p v_k = \{p_i | (p_i, v_k) \in E_V^P\}$
- p_i^v denotes the output handlers of p_i, i.e., $p_i^v = \{v_k | (p_i, v_k) \in E_V^P\}$
- v_k^p denotes the output places of v_k, i.e., $v_k^p = \{p_i | (v_k, p_i) \in E_V^P\}$
- ${}^v p_i$ denotes the input handlers of p_i, i.e., ${}^v p_i = \{v_k | (v_k, p_i) \in E_V^P\}$
- ${}^t v_k$ denotes the transitions connected to v_k, i.e., ${}^t v_k = \{t_j | \{t_j, v_k\} \in E_V^T\}$
- t_j^v denotes the handlers connected to t_j, i.e., $t_j^v = \{v_k | \{t_j, v_k\} \in E_V^T\}$

Each event handler is assigned a set of linear inequalities that relate the number of actions executed in the transitions connected to it to the marking changes in the places connected to it. More formally, each handler $v_k \in V$ is assigned two matrices (A_k, B_k) of real numbers and same number of rows. The columns of A_k are indexed by the arcs connecting v_k to places, thus, the number

of columns of A_k is $|^p v_k| + |v_k^p|$. The columns of B_k are indexed by the edges connecting transitions to v_k, thus, the number of columns of B_k is $|^t v_k|$.

For the sake of clarity in the mathematical formulation, it is assumed that each handler $v_k \in V$ is connected at least to one place and at least to one transition. This is not a limitation since a handler not connected to places can be modeled by a handler v_k connected to a fake place with a null A_k, and a handler not connected to transitions can be modeled by a handler v_k connected to a fake transition with a null B_k. Such handlers will be depicted without arcs or edges when represented graphically to provide a better intuition.

Matrix A is obtained by arranging matrices A_k *diagonally*, more precisely, A is a matrix with columns indexed by E_V^P and there is a bijection between its rows and the rows of all the matrices A_k. If the row h of A corresponds to the row q of A_k, then $A[h, (p_i, v_k)] = A_k[q, (p_i, v_k)]$ $\forall p_i \in {^p v_k}$, $A[h, (v_k, p_i)] = A_k[q, (v_k, p_i)]$ $\forall p_i \in v_k^p$ and the rest of the elements in row h of A are 0.

Matrix B is obtained similarly, B is a matrix with columns indexed by E_V^T and there is a bijection between its rows and the rows of all the matrices B_k. If the row h of B corresponds to the row q of B_k, then $B[h, \{t_j, v_k\}] = B_k[q, \{t_j, v_k\}]$ $\forall t_j \in {^t v_k}$ and the rest of the elements in row h of B are 0. Moreover, if the row h of B corresponds to the row q of B_k, then the row h of A corresponds to the row q of A_k.

1.1.2 State and events

In an event net, every place and transition is assigned a number of marks, which can be depicted as black dots. The marks in the places are called tokens, a given distribution of tokens is called marking. The number of tokens in a place is the number of components (elements or instances) of the type of components modeled by the place.

The marks in the transitions are called actions. The operations modeled by the transitions require time to carry out its job, i.e., they have time requirements which, for instance, might be related to the allocation of a number of resources during a given time period. An action in a transition means that the time requirements of the operation are satisfied, i.e., the required resources have been allocated during enough time. Thus, in contrast to tokens, actions require time to be produced. The production rate of actions is determined by the intensity net, see subsection 1.2.

The actions of a transition can be executed, i.e., the state change they model is carried out, by the firing of event handlers connected to it. The firing of an event handler v_k decreases the number of available actions in ${^t v_k}$, consumes tokens in ${^p v_k}$ and produces tokens in v_k^p. The number of actions executed, and the number of tokens consumed and produced are determined by the matrices A_k and B_k. Such a firing is instantaneous, i.e., the decrease of available actions and the produced marking change happen simultaneously in 0 time units.

Except event handlers, every element of the net is assigned one ore more variables that define the state of the net. Formally:

Definition 2 (State) *The state of the event net is given by the tuple* $(\sigma, a_T, a_E, \Delta m, m)$, *where:*

- $\sigma \in \mathbb{R}_{\geq 0}^{|T|}$ *is a vector indexed by* T *where* $\sigma[t_j]$ *is the number of actions produced in* t_j,

- $a_T \in \mathbb{R}_{\geq 0}^{|T|}$ *is a vector indexed by* T *where* $a_T[t_j]$ *is the number of actions available in* t_j*,*

- $a_E \in \mathbb{R}_{\geq 0}^{|E_V^T|}$ *is a vector indexed by* E_V^T *where* $a_E[\{t_j, v_k\}]$ *is the number of actions of* t_j *executed by* v_k*,*

- $\Delta m \in \mathbb{R}_{\geq 0}^{|E_V^P|}$ *is a vector indexed by* E_V^P *where* $\Delta m[(p_i, v_k)]$ *is the number of tokens in* p_i *consumed by* v_k*, and* $\Delta m[(v_k, p_i)]$ *is the number of tokens in* p_i *produced by* v_k*,*

- $m \in \mathbb{R}_{\geq 0}^{|P|}$ *is the marking, i.e., a vector indexed by* P *where* $m[p_i]$ *is the number of tokens in* p_i*.*

The number of actions produced is equal to the number of actions executed plus the number of actions available, hence, it holds:

$$\sigma[t_j] = a_T[t_j] + \sum_{v_k \in t_j^v} a_E[\{t_j, v_k\}] \quad \forall\, t_j \in T \tag{1}$$

On the other hand, the number of tokens in a place p_i is equal to the initial number of tokens, which is denoted $m_0[p_i]$, minus the number of tokens consumed plus the number of tokens produced:

$$m[p_i] = m_0[p_i] - \sum_{v_k \in p_i^v} \Delta m[(p_i, v_k)] + \sum_{v_k \in {}^v p_i} \Delta m[(v_k, p_i)] \quad \forall\, p_i \in P \tag{2}$$

Since actions need time to be produced, at the initial state it holds $\sigma = 0$, $a_T = 0$ and $a_E = 0$. The event net establishes how the state evolves as actions are produced and event handlers fire.

Let us first describe the states at which handlers are enabled, and hence, can fire.

Definition 3 (Enabling) *Event handler* v_k *is enabled at* $(\sigma, a_T, a_E, \Delta m, m)$ *if a vector* $a_f \in \mathbb{R}_{\geq 0}^{|{}^t v_k|}$ *indexed by* ${}^t v_k$*, and a vector* $\Delta m_f \in \mathbb{R}_{\geq 0}^{|{}^p v_k| + |v_k^p|}$ *indexed by* ${}^p v_k \cup v_k^p$ *exist such that:*

$$a_f[\{t_j, v_k\}] \leq a_T[t_j] \quad \forall\, t_j \in {}^t v_k \tag{3a}$$

$$A_k \Delta m_f \leq B_k a_f \tag{3b}$$

$$\Delta m_f[(p_i, v_k)] \leq m[p_i] \quad \forall\, p_i \in {}^p v_k \tag{3c}$$

$$\mathbf{1} a_f + \mathbf{1} \Delta m_f > 0 \tag{3d}$$

Inequality (3a) guarantees that enough actions are available, (3b) relates the number of actions to be executed to the number of tokens to be consumed and produced, (3c) guarantees that enough tokens are available to be consumed, (3d) guarantees that the overall state change is not null.

Definition 4 (Firing) *A handler* v_k *enabled at* $(\sigma, a_T, a_E, \Delta m, m)$ *can fire. The firing of* v_k *leads instantaneously to a new state* $(\sigma, a'_T, a'_E, \Delta m', m')$ *where*

only the variables associated with edges, arcs, places and transitions connected to v_k *are updated as follows:*

$$a'_T[t_j] = a_T[t_j] - a_f[\{t_j, v_k\}] \qquad \forall\ t_j \in {}^t v_k \tag{4a}$$

$$a'_E[\{t_j, v_k\}] = a_E[\{t_j, v_k\}] + a_f[\{t_j, v_k\}] \qquad \forall\ t_j \in {}^t v_k \tag{4b}$$

$$\Delta m'[(p_i, v_k)] = \Delta m[(p_i, v_k)] + \Delta m_f[(p_i, v_k)] \qquad \forall\ p_i \in {}^p v_k \tag{4c}$$

$$\Delta m'[(v_k, p_i)] = \Delta m[(v_k, p_i)] + \Delta m_f[(v_k, p_i)] \qquad \forall\ p_i \in v_k^p \tag{4d}$$

$$m'[p_i] = m[p_i] - \Delta m_f[(p_i, v_k)] + \Delta m_f[(v_k, p_i)] \quad \forall\ p_i \in {}^p v_k \cup v_k^p \tag{4e}$$

where a_f *and* Δm_f *satisfy* (3)*.*

In (4e), $\Delta m_f[(p_i, v_k)]$ (resp. $\Delta m_f[(v_k, p_i)]$) is taken as 0 if there is no arc (p_i, v_k) (resp. (v_k, p_i)).

Notice that an enabled handler is not forced to fire, and that the state reached by the firing of an event handler is allowed to be nondeterministic, this is due to the inequality (3b). Moreover, the firing does not force the execution of a minimum number of actions nor the consumption or production of a minimum number of tokens. In fact, equations (4) are trivially satisfied with $a_f = 0$ and $\Delta m_f = 0$. Thus, (4) also holds for every non enabled handler with $a_f = 0$ and $\Delta m_f = 0$.

The overall change in the state produced by several firings is the result of adding the changes produced by each firing. This leads to a set of equations that are satisfied by the states that can be reached from a given initial state.

Proposition 1 (State equations) *Let us consider the state* $(\sigma, \sigma, 0, 0, m_0)$*, i.e.,* σ *actions are available and no event handler has fired. Every state* $(\sigma, a_T, a_E, \Delta m, m)$ *reachable from* $(\sigma, \sigma, 0, 0, m_0)$ *belongs to* $SE_{\mathcal{N}_V}(\sigma, m_0)$ *where:*

$$SE_{\mathcal{N}_V}(\sigma, m_0) = \{(\sigma, a_T, a_E, \Delta m, m) | \sigma = a_T + Y_\sigma a_E \\ A\Delta m \leq B a_E \\ m = m_0 + Z_m \Delta m\} \tag{5}$$

where Y_σ *and* Z_m *are matrices determined by the net structure:*

- Y_σ *is a matrix with rows indexed by* T*, columns indexed by* E_V^T*, and such that* $Y_\sigma[t_j, \{t_j, v_k\}] = 1\ \forall\ \{t_j, v_k\} \in E_V^T$ *and the rest of the elements in* Y_σ *are* 0*,*

- Z_m *is a matrix with rows indexed by* P*, columns indexed by* E_V^P*, and such that* $Z_m[p_i, (p_i, v_k)] = -1\ \forall\ (p_i, v_k) \in E_V^P$*,* $Z_m[p_i, (v_k, p_i)] = 1\ \forall\ (v_k, p_i) \in E_V^P$ *and the rest of the elements in* Z_m *are* 0*,*

and a_T*,* a_E*,* Δm *and* m *are nonnegative variables.*

Informally, the role of matrix Y_σ is to distribute the actions in transitions among the handlers connected to them, see (1). The role of Z_m is to collect and add the marking changes produced by the firings, see (2).

Notice that equations (5) account for the additive effect, and not the sequence, of the firings. In particular, the availability of tokens and actions consumed by the sequence of firings is not checked. This can lead to spurious

solutions [5] in the state equations. Hence, Equations (5) represent a necessary condition for the reachability of $(\sigma, a_T, a_E, \Delta m, m)$.

Notice also that the number of executed actions a_E and marking changes Δm cannot decrease as event handlers fire, i.e., a_E and Δm are nondecreasing functions.

If both, the action variables (σ, a_T, a_E) and the token variables (m, Δm) are nonnegative integer numbers, then the evolution of the event net is discrete. The trajectory of a given variable can be continuous if it is not constrained to the integers but to the nonnegative real numbers. A hybrid evolution, combining both discrete and continuous trajectories, can be obtained if some variables are constrained to the nonnegative integers and the rest to the nonnegative reals.

1.1.3 Bounded initial marking

In order to model the fact that the initial marking might be partially unknown, m_0 is assumed to be a vector constrained as:

$$J_m m_0 \leq K_m \tag{6}$$

where matrices J_m and K_m must satisfy that there exist $m_0 \geq 0$ such that $J_m m_0 \leq K_m$.

Equations (5) can be easily modified to take into account uncertain initial markings:

$$\begin{aligned} SE_{\mathcal{N}_V}(\sigma, J_m, K_m) = \{(\sigma, a_T, a_E, \Delta m, m) | & \sigma = a_T + Y_\sigma a_E \\ & A\Delta m \leq B a_E \\ & m = m_0 + Z_m \Delta m \\ & J_m m_0 \leq K_m\} \end{aligned} \tag{7}$$

1.1.4 Forcing executions

Enabled event handlers are not forced to fire. However, in some cases it might be useful to consider only the states of $SE_{\mathcal{N}_V}(\sigma, J_m, K_m)$ that have executed all the actions of some transitions. Let $T_F \subseteq T$ be the set of transitions whose actions must have been executed, i.e., the number of available actions of $t_j \in T_F$ in any state in $SE_{\mathcal{N}_V}(\sigma, J_m, K_m)$ is 0. This requirement can be taken into account by:

$$\begin{aligned} SE_{\mathcal{N}_V}(\sigma, J_m, K_m) = \{(\sigma, a_T, a_E, \Delta m, m) | & \sigma = a_T + Y_\sigma a_E \\ & A\Delta m \leq B a_E \\ & m = m_0 + Z_m \Delta m \\ & J_m m_0 \leq K_m \\ & a_T[t_j] = 0 \quad \forall\ t_j \in T_F\} \end{aligned} \tag{8}$$

Notice that (8) can be empty, i.e., infeasible, if not all the actions of the transitions in T_F can be executed.

1.1.5 Modeling examples

Consider a reaction with uncertain stoichiometry as $R : nA + 2nB \to C$ where n is known to be between 10 and 12. That is, the production of one molecule of

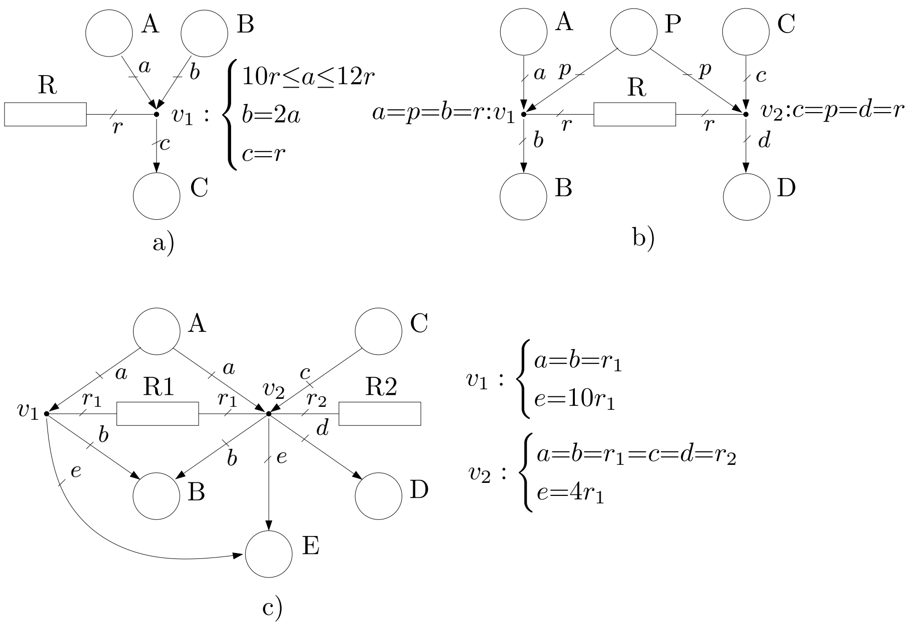

Figure 1: Event net. Modeling examples.

type C requires a quantity of molecules of type A that is in $[10, 12]$ and twice as many molecules of type B. Such a reaction can be modeled by the event net in Figure 1 (a). In the net, labels are associated with the arcs and edges, and a set of equations is associated with the event handler to specify the relationships among the number of actions and tokens consumed and produced. The matrices associated with the handler v_1 are:

$$A_1 = \begin{pmatrix} -1 & 0 & 0 \\ 1 & 0 & 0 \\ 2 & -1 & 0 \\ -2 & 1 & 0 \\ 0 & 0 & -1 \\ 0 & 0 & 1 \end{pmatrix} ; \quad B_1 = \begin{pmatrix} -10 \\ 12 \\ 0 \\ 0 \\ -1 \\ 1 \end{pmatrix}$$

where the indexes of the columns of A_1 are ordered as (A, v_1), (B, v_1) and (v_1, C). Matrices Y_σ and Z_m are:

$$Y_\sigma = \begin{pmatrix} 1 \end{pmatrix} ; \quad Z_m = \begin{pmatrix} -1 & 0 & 0 \\ 0 & -1 & 0 \\ 0 & 0 & 1 \end{pmatrix}$$

where the indexes of the columns of Z_m are ordered as the ones of A_1.

The net in Figure 1 b) models a choice in a transition. In a chemical context, this net might model a reaction R that either takes A and P as reactants and B as product, or C and P as reactants and D as product, i.e., $R : \begin{cases} A + P \to B \\ C + P \to D \end{cases}$.

This net can be interpreted in different ways. If the system is being observed, the transition can model an event that can be observed, and whose occurrence

can produce different state changes (such changes being modeled by the event handlers connected to the transition). For instance, the transition can model phosphorylation, but it is not possible to determine by the observer whether molecule A or C has been phosphorylated. If the net is being controlled, the transition could model an input action whose effect on the system is not fully controllable as any of the connected handlers can fire.

Notice that the two handlers could be merged into a single one with appropriate associated matrices. However, the higher the number of handlers, the more informative the graphical representation of the net.

In a similar way to Figure 1 b), it is straightforward to design event nets for reactions of the form $R : \begin{cases} A + B \to Z \\ C + D \to Z \end{cases}$ or $R : \begin{cases} A \to U + V \\ A \to X + Y \end{cases}$. Roughly speaking, it can be said that the event net can model reactions in which both, the reactants and the products, are expressed in a way similar to a disjunctive normal form.

The net in Figure 1 c) models two reactions $R1$ and $R2$. Reaction $R1$ is $R1 : A \to B + 10E$ where place E models energy, i.e., ten energy units are released for each occurrence of $R1$. The reactant of $R2$ is C and its product is D. Reaction $R2$ is coupled to $R1$ as it can only occur simultaneously to $R1$. The overall energy released after the simultaneous occurrence of both reactions is 4.

A modeling example of an event handler not connected to transitions can be found in Subsection 1.3.3. Such a handler models a marking transformation that requires no action. Recall that actions require time to be produced, hence, the marking transformation does not require time, and can happen instantaneously as soon as there are enough tokens in the input places. Notice that this behavior is not equivalent to that of immediate transitions (for instance, the ones proposed in [1]) in which the states with enabled immediate transition are necessarily *vanishing* and no time is spent on them. In contrast, an event handler that is not connected to transitions and that is enabled at a given state is not forced to fire, and hence time can be spent on such a state.

An event handler not connected to places would model a sink of actions, i.e., the actions in the edges connected to it would produce no marking changes.

1.2 Intensity net

The intensity net determines the intensities or rates at which the actions are produced in transitions. Physically, intensities can be interpreted as: speed of a transition, power applied to perform the work represented by the transition, heat flow which produces heat over time, etc.

These intensities depend on the marking of the places connected to the transitions. An intensity net can be denoted as a $P/S/T$ net, i.e., tokens in places P produce intensities in transitions T through intensity handlers S. In turn, intensities produce actions in transitions along time.

1.2.1 Net structure

The changes in the intensities are produced by tokens in the intensity net in the same way the changes in the markings are produced by actions in the event net.

Thus, the concepts underlying the definition of the intensity net are similar to the ones of the event net but with their own semantics.

Definition 5 (Intensity net) *An intensity net is a tuple* $\mathcal{N}_S = (P, T, S, E_S, C, D)$ *where* (P, T, S, E_S) *is a tripartite graph determining the net structure and* (C, D) *are matrices establishing how the markings produce intensities in the transitions.*

The set of vertices of the net is partitioned into three sets, P is the set of places, T is the set of transitions, and:

- $S = \{s_1, \ldots, s_l, \ldots\}$ is a set of $|S|$ intensity handlers.

Places and transitions model the same system features as in the event net. The intensity handlers are depicted as dots and model the different ways in which the markings can generate intensities in the transitions.

The vertices of the net are connected by the edges in E_S. Each pair of vertices can be connected by at most one edge. The set E_S is partitioned into two sets E_S^T and E_S^P, where E_S^T is a set of directed edges (or simply arcs) connecting transitions to intensity handlers and vice versa, and E_S^P is a set of undirected edges (or simply edges) connecting places and intensity handlers. Thus, although event handlers and intensity handlers are represented as dots, they can be easily distinguished by the arcs and edges that connect them to transitions and places. More formally:

- Every $e \in E_S^T$ is either an arc $e = (t_j, s_l)$ from a transition t_j to a handler s_l, or an arc $e = (s_l, t_j)$ from a handler s_l to a transition t_j.
- Every $e \in E_S^P$ is an edge $e = \{p_i, s_l\}$ connecting a place p_i and a handler s_l.

As in the event net, connections among places and transitions are not allowed. The following notation will be used:

- ${}^t s_l$ denotes the input transitions of s_l, i.e., ${}^t s_l = \{t_j | (t_j, s_l) \in E_S^T\}$
- t_j^s denotes the output handlers of t_j, i.e., $t_j^s = \{s_l | (t_j, s_l) \in E_S^T\}$
- s_l^t denotes the output transitions of s_l, i.e., $s_l^t = \{t_j | (s_l, t_j) \in E_S^T\}$
- ${}^s t_j$ denotes the input handlers of t_j, i.e., ${}^s t_j = \{s_l | (s_l, t_j) \in E_S^T\}$
- ${}^p s_l$ denotes the places connected to s_l, i.e., ${}^p s_l = \{p_i | \{p_i, s_l\} \in E_S^P\}$
- p_i^s denotes the handlers connected to p_i, i.e., $p_i^s = \{s_l | \{p_i, s_l\} \in E_S^P\}$

Each handler $s_l \in S$ is assigned two matrices (C_l, D_l) of real numbers and same number of rows. The columns of C_l are indexed by the arcs connecting s_l to transitions, thus, the number of columns of C_l is $|{}^t s_l| + |s_l^t|$. The columns of D_l are indexed by the edges connecting places to s_l, thus, the number of columns of D_l is $|{}^p s_l|$.

As in the event net and without loss of generality, it is assumed that each handler $s_l \in S$ is connected to at least one place and at least to one transition.

Matrix C is obtained by arranging matrices C_l *diagonally*, more precisely, C is a matrix with columns indexed by E_S^T and there is a bijection between

its rows and the rows of all the matrices C_l. If the row h of C corresponds to the row q of C_l, then $C[h,(t_j,s_l)] = C_l[q,(t_j,s_l)]\ \forall t_j \in {}^t s_l$, $C[h,(s_l,t_j)] = C_l[q,(s_l,t_j)]\ \forall t_j \in s_l^t$ and the rest of the elements in row h of C are 0.

Matrix D is obtained similarly, D is a matrix with columns indexed by E_S^P and there is a bijection between its rows and the rows of all the matrices D_l. If the row h of D corresponds to the row q of D_l, then $D[h,\{p_i,s_l\}] = D_l[q,\{p_i,s_l\}]\ \forall p_i \in {}^p s_l$ and the rest of the elements in row h of D are 0. Moreover, if the row h of D corresponds to the row q of D_l, then the row h of C corresponds to the row q of C_l.

1.2.2 State and intensities

As in the event net, the places in the intensity net contain tokens. These tokens can be used by the intensity handlers to produce intensities in the transitions. A token is active if it is being used by an intensity handler, otherwise it is idle. The intensity handlers determine how much intensity is produced in each transition depending on the marking. Over time, intensities produce actions which can be used by the event net to change the marking.

Except intensity handlers, every element of the net is assigned one ore more variables that define its state. Formally:

Definition 6 (State) *The state of the intensity net is given by the tuple* $(m, \mu_P, \mu_E, \Delta\lambda, \lambda)$*, where:*

- $m \in \mathbb{R}_{\geq 0}^{|P|}$ *is the marking, i.e., a vector indexed by* P *where* $m[p_i]$ *is the number of tokens in* p_i*,*
- $\mu_P \in \mathbb{R}_{\geq 0}^{|P|}$ *is a vector indexed by* P *where* $\mu_P[p_i]$ *is the number of idle tokens in* p_i*,*
- $\mu_E \in \mathbb{R}_{\geq 0}^{|E_S^P|}$ *is a vector indexed by* E_S^P *where* $\mu_E[\{p_i,s_l\}]$ *is the number of active tokens of* p_i *being used by* s_l*,*
- $\Delta\lambda \in \mathbb{R}_{\geq 0}^{|E_S^T|}$ *is a vector indexed by* E_S^T *where* $\Delta\lambda[(t_j,s_l)]$ *is a negative change of intensity in* t_j *produced by* s_l*, and* $\Delta\lambda[(s_l,t_j)]$ *is a positive change of intensity in* t_j *produced by* s_l*,*
- $\lambda \in \mathbb{R}_{\geq 0}^{|T|}$ *is a vector indexed by* T *where* $\lambda[t_j]$ *is the intensity produced in* t_j*.*

While idle tokens are associated with places, active tokens are associated with the edges of the intensity net. The intensity handlers produce intensities in the arcs, the overall intensity change is accounted in transitions. An intensity handler is said to be working when it is using tokens to produce intensities. When an intensity handler s_l starts working (or increases its working rate), the number of idle tokens in ${}^p s_l$ decreases, the number of active tokens in its edges increases (such tokens start being used by the handler), and intensities are produced in its arcs. Conversely, when an intensity handler s_l stops working (or decreases its working rate), the number of idle tokens in ${}^p s_l$ increases (i.e., they are released by the handler), the number of active tokens becomes 0, and no intensities are produced in its arcs.

Thus, in contrast to the firing of event handlers whose firing, i.e., action execution, cannot be reversed once it has occurred, intensity handlers are allowed to increase and decrease their working rates, thus allocating tokens as active tokens and releasing them as idle tokens, over time. This implies that, in contrast to executed actions a_E and marking changes Δm in the event net, the number of active tokens μ_E and the intensity changes $\Delta\lambda$ do not need to be nondecreasing functions.

The number of tokens is equal to the number of idle tokens plus the number of active tokens, hence, it holds:

$$m[p_i] = \mu_P[p_i] + \sum_{s_l \in p_i^s} \mu_E[\{p_i, s_l\}] \quad \forall\, p_i \in P \tag{9}$$

Similarly to the initial marking of places, each transition t_j is assigned an initial (or default) intensity $\lambda_0[t_j]$. The intensity $\lambda[t_j]$ in a transition t_j is equal to $\lambda_0[t_j]$ plus the positive changes of intensity minus the negative changes of intensity:

$$\lambda[t_j] = \lambda_0[t_j] - \sum_{s_l \in t_j^s} \Delta\lambda[(t_j, s_l)] + \sum_{s_l \in {}^s t_j} \Delta\lambda[(s_l, t_j)] \quad \forall\, t_j \in T \tag{10}$$

The amounts of active tokens, $\mu_w \in \mathbb{R}_{\geq 0}^{|{}^p s_l|}$ indexed by ${}^p s_l$, being used by an intensity handler s_l, and the amounts of intensities, $\Delta\lambda_w \in \mathbb{R}_{\geq 0}^{|{}^t s_l| + |s_l^t|}$ indexed by ${}^t s_l \cup s_l^t$, produced by the handler are related by the matrices C_l and D_l as:

$$C_l \Delta\lambda_w \leq D_l \mu_w \tag{11}$$

If $\mathbf{1}\mu_w + \mathbf{1}\Delta\lambda_w > 0$, then s_l is said to be working. Similarly to event handlers, intensity handlers are not forced to work. When a number of intensity handlers work simultaneously, they share the tokens in places and collaborate in the production of intensities.

Mathematically, intensity nets and event nets relate their variables in a similar way, e.g., intensity changes, idle tokens and active tokens have their mathematically equivalent in the event net as marking changes, available actions and executed actions. Thus, the overall number of active and idle tokens, and the produced intensities can be expressed as in (5). The state equations of the intensity net determine the potential states of the net for a given given marking m and default intensities λ_0:

Proposition 2 (State equations) *Let us consider the state $(m, m, 0, 0, \lambda_0)$, i.e., m idle tokens are available and no intensity handler is working. Every state $(m, \mu_P, \mu_E, \Delta\lambda, \lambda)$ reachable from $(m, m, 0, 0, \lambda_0)$ belongs to $SE_{\mathcal{N}_S}(m, \lambda_0)$ where:*

$$\begin{aligned} SE_{\mathcal{N}_S}(m, \lambda_0) = \{(m, \mu_P, \mu_E, \Delta\lambda, \lambda) | & m = \mu_P + Y_m \mu_E \\ & C\Delta\lambda \leq D\mu_E \\ & \lambda = \lambda_0 + Z_\lambda \Delta\lambda\} \end{aligned} \tag{12}$$

where Y_m and Z_λ are matrices determined by the net structure:

- *Y_m is a matrix with rows indexed by P, columns indexed by E_S^P, and such that $Y_m[p_i, \{p_i, s_l\}] = 1$ $\forall \{p_i, s_l\} \in E_S^P$ and the rest of the elements in Y_m are 0,*

- *Z_λ is a matrix with rows indexed by T, columns indexed by E_S^T, and such that $Z_\lambda[t_j,(t_j,s_l)] = -1\ \forall(t_j,s_l) \in E_S^T$, $Z_\lambda[t_j,(s_l,t_j)] = 1\ \forall(s_l,t_j) \in E_S^T$ and the rest of the elements in Z_λ are 0,*

and μ_P, μ_E, $\Delta\lambda$ and λ are nonnegative variables.

As in (5), equations (12) account for the additive intensities produced by the handlers, and hence, $SE_{\mathcal{N}_S}(m,\lambda_0)$ can contain spurious solutions. Similarly to the event net, the variables of the intensity net can be either discrete or continuous. If the token variables m, μ_P, μ_E are nonnegative integers, then the resources they model cannot be split indefinitely, e.g., servers or machines, otherwise they can, e.g., concentration of enzymes (if it is modeled as a real number). If the intensity variables λ, $\Delta\lambda$ are nonnegative integers, then the activities modeled by the transitions can only operate at speeds contained in a discrete set, otherwise theirs speeds can lie in a continuous set.

1.2.3 Bounded default intensities

Partially unknown default intensities λ_0 can be handled similarly as partially unknown initial markings. Let us assume that λ_0 is a vector constrained as:

$$J_\lambda \lambda_0 \leq K_\lambda \tag{13}$$

where matrices J_λ and K_λ must satisfy that there exist $\lambda_0 \geq 0$ such that $J_\lambda \lambda_0 \leq K_\lambda$.

Equations (12) can be modified to handle uncertain defaults intensities:

$$\begin{aligned} SE_{\mathcal{N}_S}(m,J_\lambda,K_\lambda) = \{(m,\mu_P,\mu_E,\Delta\lambda,\lambda) | & m = \mu_P + Y_m\mu_E \\ & C\Delta\lambda \leq D\mu_E \\ & \lambda = \lambda_0 + Z_\lambda\Delta\lambda \\ & J_\lambda\lambda_0 \leq K_\lambda\} \end{aligned} \tag{14}$$

1.2.4 Forcing active tokens

Intensity handlers are not forced to work. However, in some cases it might be informative to consider only the states of $SE_{\mathcal{N}_S}(m,J_\lambda,K_\lambda)$ at which all the tokens of a given set of places are active. Let $P_F \subseteq P$ be the set of places whose tokens must be active, i.e., the number of idle tokens of $p_i \in P_F$ in any state in $SE_{\mathcal{N}_S}(m,J_\lambda,K_\lambda)$ is 0. This requirement can be taken into account by:

$$\begin{aligned} SE_{\mathcal{N}_S}(m,J_\lambda,K_\lambda) = \{(m,\mu_P,\mu_E,\Delta\lambda,\lambda) | & m = \mu_P + Y_m\mu_E \\ & C\Delta\lambda \leq D\mu_E \\ & \lambda = \lambda_0 + Z_\lambda\Delta\lambda \\ & J_\lambda\lambda_0 \leq K_\lambda \\ & \mu_P[p_i] = 0 \ \ \forall\ p_i \in P_F\} \end{aligned} \tag{15}$$

Notice that (15) can be empty, i.e., infeasible, if not all the tokens of the the places in P_F can be active.

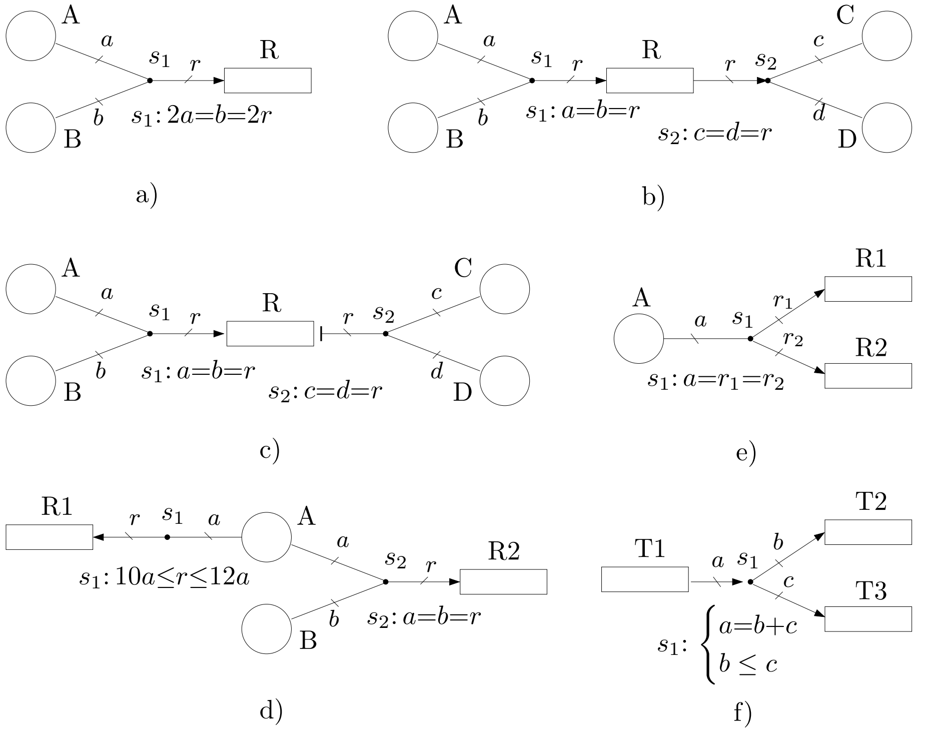

Figure 2: Intensity net. Modeling examples.

1.2.5 Modeling examples

Given that event nets and intensity nets are topologically similar (marking changes in an event net are produced as intensity changes in an intensity net), similar features to those of the event nets can be modeled by intensity nets .

The intensity net in Figure 2 a) establishes a synchronization between the tokens in A and the tokens in B. More precisely, the equation assigned to the intensity handler s_1 states that when a token in A synchronizes with two tokens in B, an intensity of one unit is produced in transition R, i.e., $2\mu_E[(A, s_1)] = \mu_E[(B, s_1)] = 2\Delta\lambda[\{s_1, R\}] = 2\lambda[R]$.

In the net in Figure 2 b), a token in A together with a token in B produce an increase of one unit in the intensity of R. Similarly, a token in C together with a token in D produce a decrease of one unit in the intensity of R. Thus, the tokens in A and B can be seen as enhancers and the tokens in C and D as repressors. As in the event net, the intensity can be expressed as a *disjunctive normal form* of enhancers and repressors. The intensity arc (R, s_2) can be equivalently depicted as in Figure 2 c).

The net in Figure 2 d) models a choice in place A, i.e., a token in A can be used either to produce an intensity within the interval $[10, 12]$ in $R1$ or, together with a token in B an intensity in $R2$ of one unit.

In Figure 2 e), a token in (A, s_1) produces simultaneously an intensity of one unit both in $R1$ and $R2$, i.e., it can be said that the intensities of $R1$ and $R2$ are synchronized by the tokens in A.

Figure 2 f) shows an intensity net without places. If the default intensity

of $T1$ is positive, e.g., $\lambda_0[T1] = 5$, then intensity can be transferred from $T1$ to $T2$ and $T3$ according to the inequalities in the intensity handler. Notice that in contrast to the production and consumption of tokens by event arcs, the intensities at intensity arcs can increase and decrease over time, more formally, Δm is nondecreasing and $\Delta\lambda$ is not.

Consider an intensity handler s_1 not connected to places with an arc (s_1, t) to transition t_1 and with associated matrices $C_1 = (0)$ and $D_1 = (0)$. This would model that tokens are not required to produce intensities and, in fact, the intensity that can be produced in t is not upper bounded. However, in contrast to immediate transitions, this does not imply that the produced intensity is infinite.

An intensity handler not connected to transitions would model the fact that the tokens in the edges connected to it do not produce intensity changes.

1.3 Flexible net. Definition and state equations

1.3.1 Definition

A flexible net is composed of an event net and an intensity net that have the same set of places and the same set of transitions.

Definition 7 (Flexible net) *A flexible net is a tuple* $\mathcal{N} = (P, T, V, E_V, A, B, S, E_S, C, D)$ *where* (P, T, V, E_V, A, B) *is an event net and* (P, T, S, E_S, C, D) *is an intensity net.*

In a flexible net, the event net determines the way actions produce marking changes, and the intensity net determines the way tokens produce intensity changes. Thus, on the one hand, the intensity net can be seen as the engine powering the event net. And, on the other hand, the events occurring in the event net determine how intensities can be produced. A flexible net can be denoted as a $P/H/T$ net, i.e., places P and transitions T are connected through handlers.

1.3.2 State equations

Structurally, the event net and the intensity net are connected by places and transitions. From the point of view of the state variables, they are linked through the integration of $\Delta\lambda$, and consequently λ, over time what leads to the number of produced actions σ. Thus, a flexible net is a time continuous model, where time, denoted as τ, is the independent variable.

In the same way that $\Delta\lambda$ denotes intensities in arcs, let $\Delta\sigma$ denote actions produced in arcs in E_S^T. The intensities in the intensity net produce actions as follows. Each arc e in E_S^T is assigned a function $f_e : \mathbb{R} \to \mathbb{R}$ that maps the integral of $\Delta\lambda[e]$ over the time interval $[0, \tau]$, to the number of actions $\Delta\sigma[e]$ produced in e up to time instant τ:

$$\Delta\sigma[e](\tau) = f_e(\int_0^\tau \Delta\lambda[e](s)\,\mathrm{d}s) \quad \forall e \in E_S^T \tag{16}$$

For instance, f_e could be a floor function if only integer actions can be produced, or f_e could be a Poisson process if the time between two consecutive productions of actions follows an exponential distribution, in such a case, $\Delta\sigma[e](\tau)$ follows

a Poisson distribution with associated parameter $\int_0^\tau \Delta\lambda[e](s)\,\mathrm{d}s$. In order to facilitate the analysis, it is assumed in the following that f_e is the identity function, in other words, it is ignored and $\Delta\sigma[e]$ is a real variable. This can be interpreted as a first order approximation of a Poisson distribution or other probability distributions. Thus:

$$\Delta\sigma(\tau) = \int_0^\tau \Delta\lambda(s)\,\mathrm{d}s \tag{17}$$

The overall number of actions produced in a transition t_j can be computed by integrating $\lambda[t_j]$, or equivalently, by making use of Z_λ, see (12):

$$\sigma(\tau) = \lambda_0\tau + Z_\lambda\Delta\sigma(\tau) \tag{18}$$

In addition to the state variables of the event and intensity net, $\Delta\sigma$ is included in the tuple of variables defining the state of the flexible net.

Definition 8 (State) *The state $\boldsymbol{x}$ of a flexible net is given by the vector that results from the concatenation of the state variables, i.e., $\boldsymbol{x} = (m, \mu_P, \mu_E, \Delta\lambda, \lambda, \Delta\sigma, \sigma, a_T, a_E, \Delta m)$.*

All the state variables are time dependent. For the sake of clarity, the time dependency will be omitted when it is clear from the context, e.g., $m(\tau)$ is shortened to m. At the initial state it holds $\Delta\sigma = 0$, $\sigma = 0$, $a_T = 0$, $a_E = 0$, $\Delta m = 0$, i.e., the initial state can be written as: $(m, \mu_P, \mu_E, \Delta\lambda, \lambda, 0, 0, 0, 0, 0)$.

The state variables a_E, Δm, $\Delta\sigma$ and σ are nondecreasing: a_E represent actions that have been executed, Δm represents consumptions and productions of tokens that have taken place, $\Delta\sigma$ and σ are integrals of the nonnegative variables $\Delta\lambda$ and λ. Thus, m, μ_P, μ_E and Δm can be classified as *marking* variables, $\Delta\lambda$ and λ as *intensity* variables, and $\Delta\sigma$, σ, a_T and a_E as *action* variables.

Notice that some of the state variables are redundant. In fact, for given m_0 and λ_0, the variables m, μ_P, λ, σ and a_T can be obtained from μ_E, $\Delta\lambda$, $\Delta\sigma$, a_E and Δm. Hence, the state of a flexible net can be also expressed as $(\mu_E, \Delta\lambda, \Delta\sigma, a_E, \Delta m)$. Moreover, $\Delta\sigma$ can be obtained from the time trajectory of $\Delta\lambda$ by (17).

By making use of $SE_{\mathcal{N}_V}(\sigma, J_m, K_m)$ (7), $SE_{\mathcal{N}_S}(m, J_\lambda, K_\lambda)$ (14), (17) and (18), it is possible to write a set of equations that any potential state at time τ must satisfy.

Proposition 3 (State equations) *Let $\mathcal{N}$ be a flexible net with initial marking m_0 satisfying $J_m m_0 \leq K_m$, and default intensities λ_0 satisfying $J_\lambda\lambda_0 \leq K_\lambda$. Every state $(m, \mu_P, \mu_E, \Delta\lambda, \lambda, \Delta\sigma, \sigma, a_T, a_E, \Delta m)$ reachable at time τ belongs to $SE_{\mathcal{N}}(\tau, J_m, K_m, J_\lambda, K_\lambda)$ where:*

$$\begin{aligned}
& SE_{\mathcal{N}}(\tau, J_m, K_m, J_\lambda, K_\lambda) = \{(m, \mu_P, \mu_E, \Delta\lambda, \lambda, \Delta\sigma, \sigma, a_T, a_E, \Delta m) | \\
& \quad m = \mu_P + Y_m\mu_E;\ C\Delta\lambda \leq D\mu_E;\ \lambda = \lambda_0 + Z_\lambda\Delta\lambda;\ J_\lambda\lambda_0 \leq K_\lambda \\
& \quad \Delta\sigma = \int_0^\tau \Delta\lambda(s)\,\mathrm{d}s;\ \sigma = \lambda_0\tau + Z_\lambda\Delta\sigma \\
& \quad \sigma = a_T + Y_\sigma a_E;\ A\Delta m \leq Ba_E;\ m = m_0 + Z_m\Delta m;\ J_m m_0 \leq K_m \\
& \quad a_T[t_j] = 0\ \ \forall\, t_j \in T_F;\ \mu_P[p_i] = 0\ \ \forall\, p_i \in P_F\}
\end{aligned} \tag{19}$$

where every variable is nonnegative.

Equations (19) can be read as follows: Some of the tokens in m are active, μ_E, and the rest are idle, μ_P. Active tokens produce intensity changes, $\Delta\lambda$, which result in overall intensities, λ, in transitions. The integral of the intensity changes and overall intensities over time produces action, σ, i.e., σ is produced as time elapses. Some of the produced actions in σ are available, a_T, and the rest were executed, a_E. The executed actions produced marking changes, Δm, which resulted in overall marking, m, in places. This behavior repeats over time: when a new marking is reached, intensities are updated, what can lead to the production and execution of new actions, what in turns results in a new marking.

Equations (19) can be relaxed by dropping their time dependency. This leads to a set of constraints that represent a necessary condition for reachability at any time.

Proposition 4 (Untimed state equations) *Let $\mathcal{N}$ be a flexible net with initial marking m_0 satisfying $J_m m_0 \leq K_m$, and default intensities λ_0 satisfying $J_\lambda \lambda_0 \leq K_\lambda$. Every state $(m, \mu_P, \mu_E, \Delta\lambda, \lambda, \Delta\sigma, \sigma, a_T, a_E, \Delta m)$ reachable at any time $\tau \geq 0$ belongs to $USE_\mathcal{N}(J_m, K_m, J_\lambda, K_\lambda)$ where:*

$$\begin{aligned} &USE_\mathcal{N}(J_m, K_m, J_\lambda, K_\lambda) = \{(m, \mu_P, \mu_E, \Delta\lambda, \lambda, \Delta\sigma, \sigma, a_T, a_E, \Delta m) | \\ &\quad m = \mu_P + Y_m \mu_E;\ C\Delta\lambda \leq D\mu_E;\ \lambda = \lambda_0 + Z_\lambda \Delta\lambda;\ J_\lambda \lambda_0 \leq K_\lambda \\ &\quad \sigma = a_T + Y_\sigma a_E;\ A\Delta m \leq B a_E;\ m = m_0 + Z_m \Delta m;\ J_m m_0 \leq K_m \\ &\quad a_T[t_j] = 0\ \ \forall\ t_j \in T_F;\ \mu_P[p_i] = 0\ \ \forall\ p_i \in P_F\} \end{aligned} \tag{20}$$

where every variable is nonnegative.

1.3.3 Modeling example

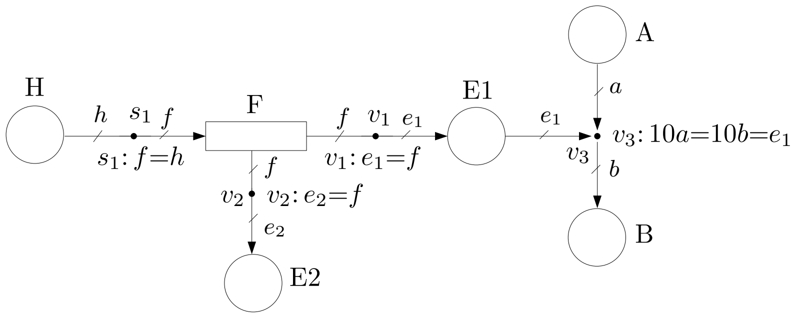

Figure 3: Flexible net. Modeling example.

The net in Figure 3 models a simple system consisting of a heater, modeled by place H, and two types of molecules, modeled by places A and B. Transition F models the heat inflow produced by the heater, which is proportional to the number of tokens in H. The system consists of two compartments and the heater cannot select the compartment to which the heat inflow goes. If the heat inflow enters the first compartment then its energy, modeled by $E1$, increases, otherwise the energy in the second compartment, modeled by $E2$, increases. If enough energy is available in $E1$ the reaction $A \to B$ can occur. Each reaction

occurrence consumes 10 energy units. As the event handler v_3 is not connected to transitions, the marking changes modeled by it do not require actions and can happen as soon as there are enough tokens in the input places.

2 Flexible net. Bounds

According to the previous section, the following features of flexible nets are nondeterministic.

With respect to the event net:

- Actions are not forced to produce marking updates, i.e., event handlers are not forced to fire, or, in other words, no execution policy is enforced.
- The choice of event handlers that handle, and hence execute, a given set of actions is nondeterministic.
- The number of tokens consumed and produced by the firing of an event handler is nondeterministic.

With respect to the intensity net:

- Tokens are not forced to produce intensities, i.e., intensity handlers are not forced to handle the intensities that tokens can produce.
- The choice of intensity handlers that handle a given set of tokens is nondeterministic.
- The intensity produced by an event handler when it handles a given set of tokens is nondeterministic.

The necessary reachability conditions that will be obtained account for all potential dynamic behaviors arising from the above nondeterministic features. More precisely, this section proposes a set of constraints that are necessarily satisfied by the state $\boldsymbol{x}$ of the flexible net at a given time θ.

2.1 Linear constraints

The equations in $SE_{\mathcal{N}}$, see (19), are difficult to solve because of the uncertain initial marking and default intensities, the integral and the potential high number of variables. In order to tackle them, we will derive some necessary conditions that any potential evolution of the net must satisfy. Such necessary conditions will be mainly based on the average values of variables over a time interval $[0, \theta]$. This will lead to a set of linear inequalities that must be satisfied by the state of the net at time θ.

The state equations (19) can be integrated to compute average values. The average state, $\bar{\boldsymbol{x}}$, over the interval $[0, \theta]$ is defined as:

$$\bar{\boldsymbol{x}}(\theta) = \frac{1}{\theta} \int_0^\theta \boldsymbol{x}(\tau) \, \mathrm{d}\tau \tag{21}$$

In the following, all the variables depend on the time parameter θ, and for the sake of clarity, it is omitted, e.g., $\bar{m}(\theta)$ is shortened to $\bar{m}$. Then, the result

of integrating (19) can be expressed as:

$$
\begin{aligned}
&\bar{m} = \bar{\mu}_P + Y_m\bar{\mu}_E;\ C\Delta\bar{\lambda} \le D\bar{\mu}_E;\ \bar{\lambda} = \lambda_0 + Z_\lambda\Delta\bar{\lambda};\ J_\lambda\lambda_0 \le K_\lambda\\
&\Delta\sigma = \Delta\bar{\lambda}\theta;\ \sigma = \lambda_0\theta + Z_\lambda\Delta\sigma\\
&\Delta\bar{\sigma} = \frac{1}{\theta}\int_0^\theta\int_0^\tau \Delta\lambda(s)\,\mathrm{d}s\,\mathrm{d}\tau;\ \bar{\sigma} = \frac{1}{2}\lambda_0\theta + Z_\lambda\Delta\bar{\sigma}\\
&\bar{\sigma} = \bar{a}_T + Y_\sigma\bar{a}_E;\ A\Delta\bar{m} \le B\bar{a}_E;\ \bar{m} = m_0 + Z_m\Delta\bar{m};\ J_m m_0 \le K_m
\end{aligned}
\tag{22}
$$

where $\bar{m}$ is the average marking, $\bar{\mu}_P$ is the average number of idle tokens, $\bar{\mu}_E$ is the average number of active tokens, $C\Delta\bar{\lambda} \le D\bar{\mu}_E$ relates the average number of active tokens to the average intensities in arcs, $\bar{\lambda}$ is the average intensity in transitions; $\Delta\sigma$ and σ are the number of actions produced in arcs and transitions respectively; $\Delta\bar{\sigma}$ and $\bar{\sigma}$ are the average number of actions produced in arcs and transitions respectively, $\bar{a}_T$ is the average number of available actions, $\bar{a}_E$ is the average number of executed actions, $\Delta\bar{m}$ is the average marking change in arcs, and $A\Delta\bar{m} \le B\bar{a}_E$ relates the average number of executed actions to the average marking change.

Since the variables a_E, Δm, $\Delta\sigma$ and σ are nondecreasing, it necessarily holds:

$$\bar{a}_E \le a_E; \Delta\bar{m} \le \Delta m; \Delta\bar{\sigma} \le \Delta\sigma; \bar{\sigma} \le \sigma \tag{23}$$

what establishes an initial relationship between the average and the final values of these variables. This allows us to obtain a set of linear inequalities (24), that must be satisfied by the state at θ, by relating the inequalities in (19) to the inequalities in (22) through (23). Notice that $J_m m_0 \le K_m$, $J_\lambda\lambda_0 \le K_\lambda$ and $\sigma = \lambda_0\theta + Z_\lambda\Delta\sigma$ appear both in (19) and (22), and hence one of the appearances can be omitted.

As in (20) , the constraints $a_T[t_j] = 0 \ \ \forall\ t_j \in T_F$ and $\mu_P[p_i] = 0 \ \ \forall\ p_i \in P_F$ are included in (24) to force the execution of the actions produced in T_F, and to force the final number of tokens in P_F to be active. Moreover, the constraints $\bar{a}_T[t_j] = 0 \ \forall\ t_j \in T_{Av}$ are added in order to model that the actions of given set of transition T_{Av} are required to be executed instantaneously, i.e., at the same rate at which they are produced. Similarly, the constraints $\bar{\mu}_P[p_i] = 0 \ \ \forall\ p_i \in P_{Av}$ are also included to model that the tokens of a given set of places P_{Av} must be active during all the interval $[0, \theta]$. Notice that, depending on the net, these two last sets of constraints can lead to an infeasible set of constraints, e.g., when actions cannot be executed instantaneously, when the number of executed actions in edges is constrained to the integer numbers, etc.

Proposition 5 (Reachable states. Linear constraints) *Let $\mathcal{N}$ be a flexible net with initial marking m_0 satisfying $J_m m_0 \le K_m$, and default intensities λ_0 satisfying $J_\lambda\lambda_0 \le K_\lambda$. Every state $(m, \mu_P, \mu_E, \Delta\lambda, \lambda, \Delta\sigma, \sigma, a_T, a_E, \Delta m)$ reachable at time θ belongs to $LC_{\mathcal{N}}(\theta, J_m, K_m, J_\lambda, K_\lambda)$ where:*

$$
\begin{aligned}
& LC_{\mathcal{N}}(\theta, J_m, K_m, J_\lambda, K_\lambda) = \{(m, \mu_P, \mu_E, \Delta\lambda, \lambda, \Delta\sigma, \sigma, a_T, a_E, \Delta m)| \\
& \quad m = \mu_P + Y_m \mu_E;\ C\Delta\lambda \leq D\mu_E;\ \lambda = \lambda_0 + Z_\lambda \Delta\lambda;\ J_\lambda \lambda_0 \leq K_\lambda \\
& \quad \sigma = a_T + Y_\sigma a_E;\ A\Delta m \leq B a_E;\ m = m_0 + Z_m \Delta m;\ J_m m_0 \leq K_m \\
& \quad \bar{m} = \bar{\mu}_P + Y_m \bar{\mu}_E;\ C\Delta\bar{\lambda} \leq D\bar{\mu}_E;\ \bar{\lambda} = \lambda_0 + Z_\lambda \Delta\bar{\lambda} \\
& \quad \Delta\sigma = \Delta\bar{\lambda}\theta;\ \sigma = \lambda_0\theta + Z_\lambda \Delta\sigma \\
& \quad \bar{\sigma} = \frac{1}{2}\lambda_0\theta + Z_\lambda \Delta\bar{\sigma} \\
& \quad \bar{\sigma} = \bar{a}_T + Y_\sigma \bar{a}_E;\ A\Delta\bar{m} \leq B\bar{a}_E;\ \bar{m} = m_0 + Z_m \Delta\bar{m} \\
& \quad \bar{a}_E \leq a_E;\ \Delta\bar{m} \leq \Delta m;\ \Delta\bar{\sigma} \leq \Delta\sigma;\ \bar{\sigma} \leq \sigma \\
& \quad a_T[t_j] = 0 \ \ \forall\, t_j \in T_F;\ \bar{a}_T[t_j] = 0 \ \ \forall\, t_j \in T_{Av} \\
& \quad \mu_P[p_i] = 0 \ \ \forall\, p_i \in P_F;\ \bar{\mu}_P[p_i] = 0 \ \ \forall\, p_i \in P_{Av}\}
\end{aligned}
\tag{24}
$$

where $\bar{m}$, $\bar{\mu}_P$, $\bar{\mu}_E$, $\Delta\bar{\sigma}$, $\bar{\sigma}$, $\bar{a}_T$, $\bar{a}_E$ and $\Delta\bar{m}$ are average values over the interval $[0, \theta]$, and every variable is nonnegative.

The polytope defined by $LC_{\mathcal{N}}$ contains every reachable state at time τ, i.e., it represents a necessary condition for reachability.

2.2 Quadratic constraints

The only information required to write the inequality $\Delta\bar{\sigma} \leq \Delta\sigma$ in (23) is that $\Delta\sigma$ is nondecreasing, i.e., $\Delta\bar{\sigma} \leq \Delta\sigma$ does not contain any information about the rate at which $\Delta\sigma$ can increase. Such a rate is given by $\Delta\lambda$. Thus, if some constraints on $\Delta\lambda$ are known, then inequality $\Delta\bar{\sigma} \leq \Delta\sigma$ can be improved. This would lead to a reduced polytope $LC_{\mathcal{N}}$, and in other words, to an improvement of the necessary condition for reachability.

We will consider two different types of constraints on $\Delta\lambda$: the first type accounts for equalities, the second one for inequalities. Such constraints might be obtained from the net structure, the initial marking, the defaults intensities, and the time span θ. See Appendix A for methods to compute bounds for linear expressions on $\Delta\lambda$.

2.2.1 Equalities

Let us first assume that constant matrices E_c and F_c exist such that $E_c \neq 0$ and it holds:

$$E_c \Delta\lambda(\tau) = F_c \ \ \forall\, \tau \in [0, \theta] \tag{25}$$

Notice that, as $\lambda = \lambda_0 + Z_\lambda \Delta\lambda$, (25) can be used to constrain λ.

The integration of (25) over $[0, \theta]$ leads to:

$$\int_0^\theta E_c \Delta\lambda(\tau)\, \mathrm{d}\tau = E_c \Delta\sigma(\theta) = \theta F_c \tag{26}$$

moreover,

$$E_c \Delta\bar{\sigma}(\theta) = \frac{1}{\theta}\int_0^\theta E_c \Delta\sigma(\tau)\, \mathrm{d}\tau = \frac{1}{\theta}\int_0^\theta \tau F_c\, \mathrm{d}\tau = \frac{\theta}{2} F_c \tag{27}$$

Thus, the following equalities can be added to $LC_{\mathcal{N}}$, see (24), to reduce it:

$$E_c \Delta\sigma = \theta F_c \tag{28}$$

$$2E_c \Delta\bar{\sigma} = E_c \Delta\sigma \tag{29}$$

2.2.2 Inequalities

Let us now assume that constant matrices E and F exist such that $E \neq 0$ and it holds:

$$E\Delta\lambda(\tau) \leq F \quad \forall\, \tau \in [0, \theta] \tag{30}$$

The integration of (30) produces:

$$E\Delta\sigma \leq \theta F \tag{31}$$

$$2E\Delta\bar{\sigma} \leq \theta F \tag{32}$$

that can be added to $LC_{\mathcal{N}}$. The inequality (32) can be improved by introducing quadratic constraints.

Let us define two vectors L and U as follows: The kth element of L is defined as $L_k = min\{E_k\Delta\lambda \mid E\Delta\lambda \leq F; \Delta\lambda \geq \mathbf{0}\}$, and the kth element of U is defined as $U_k = max\{E_k\Delta\lambda \mid E\Delta\lambda \leq F; \Delta\lambda \geq \mathbf{0}\}$, where E_k denotes the kth row of E. If the first linear program is unbounded, then L_k is not defined. In such a case, we write $L_k{=}{-\infty}$ (the linear program for U_k cannot be unbounded as it is bounded by F_k, the kth element of F).

Notice that if $L_k = U_k$ then $E_k\Delta\lambda$ is constant, and hence, the constraints considered in Subsection 2.2.1 apply. Thus, in the following it is assumed that $L_k < U_k$ for every row k.

We will distinguish two cases for each row k: a) $-\infty{<}L_k$; b) $-\infty{=}L_k$, and then obtain constraints in matrix form accounting for both cases.

Case a: If $-\infty{<}L_k{<}U_k$, then $L_k(U_k)$ is a lower(upper) bound for $E_k\Delta\lambda(\tau)$. Hence, a lower bound for $E_k\Delta\sigma(\tau)$ is given by a function $l_k(\tau)$ that initially increases at rate L_k, then at rate U_k, and reaches the value $E_k\Delta\sigma(\theta)$ at θ, see Figure 4. Similarly, an upper bound for $E_k\Delta\sigma(\tau)$ is given by a function $u_k(\tau)$ with initial rate U_k and then L_k. Thus,

$$\int_0^\theta l_k(\tau)\,\mathrm{d}\tau \leq \int_0^\theta E_k\Delta\sigma(\tau)\,\mathrm{d}\tau = \theta E_k\Delta\bar{\sigma} \leq \int_0^\theta u_k(\tau)\,\mathrm{d}\tau \tag{33}$$

The value of $\int_0^\theta u_k(\tau)\,\mathrm{d}\tau$ can be computed as $\int_0^\theta U_k\tau\,\mathrm{d}\tau{-}Area(B)$ where $Area(B)$ is the area of the triangle labeled B in Figure 4. The area of B will be obtained from the length of one of its sides, $(U_k\theta - E_k\Delta\sigma(\theta))$, and its two adjacent angles $\pi/2{+}a_{lk}$ and $\pi/2{-}a_{uk}$ with $a_{lk} = atan(L_k) \in [0,\infty)$, $a_{uk} = atan(U_k) \in [0,\infty)$:

$$\begin{aligned} Area(B) &= \frac{(U_k\theta - E_k\Delta\sigma(\theta))^2}{2(cot(\pi/2 - a_{uk}) + cot(\pi/2 + a_{lk}))} = \frac{(U_k\theta - E_k\Delta\sigma(\theta))^2}{2(tan(a_{uk}) - tan(a_{lk}))} \\ &= \frac{(U_k\theta - E_k\Delta\sigma(\theta))^2}{2(U_k - L_k)} \end{aligned} \tag{34}$$

then, by (33):

$$\theta E_k\Delta\bar{\sigma} \leq \int_0^\theta u_k(\tau)\,\mathrm{d}\tau = \int_0^\theta U_k\tau\,\mathrm{d}\tau - Area(B) = U_k\,\frac{\theta^2}{2} - \frac{(U_k\theta - E_k\Delta\sigma(\theta))^2}{2(U_k - L_k)} \tag{35}$$

Similarly, $\int_0^\theta l_k(\tau)\,\mathrm{d}\tau$ can be computed as $\int_0^\theta L_k\tau\,\mathrm{d}\tau + Area(A)$ where $Area(A)$ is the area of the triangle labeled A in Figure 4. Hence,

$$\int_0^\theta l_k(\tau)\,\mathrm{d}\tau = \int_0^\theta L_k\tau\,\mathrm{d}\tau + Area(A) = L_k\,\frac{\theta^2}{2} + \frac{(E_k\Delta\sigma(\theta) - L_k\theta)^2}{2(U_k - L_k)} \leq \theta E_k\Delta\bar{\sigma} \tag{36}$$

Case b: When $L_k = -\infty$, the upper bound (35) computed for $\theta E_k\Delta\bar{\sigma}$ becomes:

$$\theta E_k\Delta\bar{\sigma} \leq U_k\,\frac{\theta^2}{2} \tag{37}$$

A lower bound for $\theta E_k\Delta\bar{\sigma}$ when $L_k = -\infty$ can be obtained from (36) as:

$$\lim_{L_k\to-\infty}\left(L_k\,\frac{\theta^2}{2} + \frac{(E_k\Delta\sigma(\theta) - L_k\theta)^2}{2(U_k - L_k)}\right) = \theta E_k\Delta\sigma(\theta) - U_k\,\frac{\theta^2}{2} \leq \theta E_k\Delta\bar{\sigma} \tag{38}$$

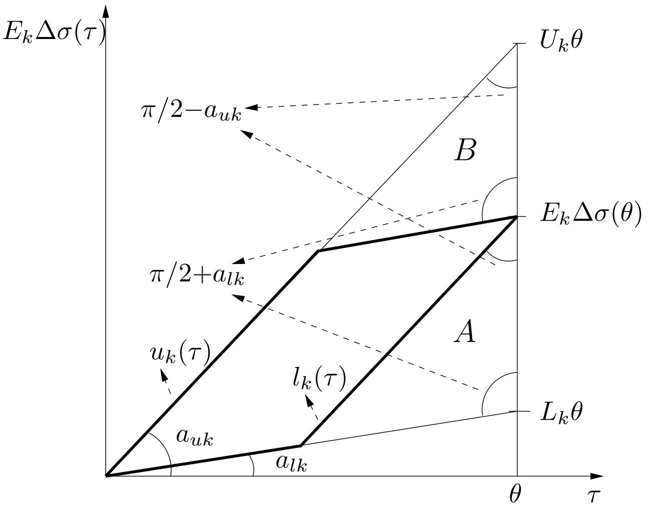

Figure 4: Lower and upper bounds of $E_k\Delta\sigma$; a_{lk} and a_{uk} are the piece-wise linear functions in bold.

Matrix form: Constraints (35) and (37) can be expressed in matrix form as:

$$\theta E\Delta\bar{\sigma} \leq \frac{1}{2}(U\,\theta^2 - (U\theta{-}E\Delta\sigma)G(U\theta{-}E\Delta\sigma)) \tag{39}$$

where $\Delta\sigma$ is short for $\Delta\sigma(\theta)$ and G is a diagonal matrix defined as:

$$G[k,k] = \begin{cases} \frac{1}{U_k - L_k} & \text{if } -\infty < L_k < U_k \\ 0 & \text{otherwise} \end{cases} \tag{40}$$

In a similar way, constraints (36) and (38) can be expressed as:

$$\frac{1}{2}(M\,\theta^2 + (E\Delta\sigma{-}L\theta)G(E\Delta\sigma{-}L\theta)) + \theta N\Delta\sigma \leq \theta E\Delta\bar{\sigma} \tag{41}$$

where M is a vector and N is a matrix defined as:

$$M[k] = \begin{cases} L_k & \text{if } -\infty < L_k \\ -U_k & \text{otherwise} \end{cases} \tag{42}$$

$$N_k = \begin{cases} 0 & \text{if } -\infty < L_k \\ E_k & \text{otherwise} \end{cases} \tag{43}$$

where N_k denotes the kth row of N.

Since G is positive semidefinite, the quadratic constraints in (39) and (41) are convex.

The matrix E can be used both to force desired constraints on the arc intensities, and to further constraint the potential states of the net and hence improve the accuracy of the results. For the latter purpose, it is usually advisable to associate two rows, E_e and E'_e, of E with each arc e in E_S^T such that $E_e[e] = 1$ and $E'_e[e] = -1$, and the rest of the elements of the rows are 0, see Appendix A for methods to compute the corresponding values of F.

2.3 Reachability conditions

The constraints (28), (29), (31), (39) and (41) can be included in $LC_{\mathcal{N}}$, see (24), to account for the intensity bounds.

Proposition 6 (Reachable states. Quadratic constraints) *Let $\mathcal{N}$ be a flexible net with initial marking m_0 satisfying $J_m m_0 \leq K_m$, and default intensities λ_0 satisfying $J_\lambda \lambda_0 \leq K_\lambda$. Every state $(m, \mu_P, \mu_E, \Delta\lambda, \lambda, \Delta\sigma, \sigma, a_T, a_E, \Delta m)$ reachable at time θ belongs to $TC_{\mathcal{N}}(\theta, J_m, K_m, J_\lambda, K_\lambda)$ where:*

$$\begin{aligned}
&TC_{\mathcal{N}}(\theta, J_m, K_m, J_\lambda, K_\lambda) = \{(m, \mu_P, \mu_E, \Delta\lambda, \lambda, \Delta\sigma, \sigma, a_T, a_E, \Delta m) | \\
&\quad m = \mu_P + Y_m \mu_E;\ C\Delta\lambda \leq D\mu_E;\ \lambda = \lambda_0 + Z_\lambda \Delta\lambda;\ J_\lambda \lambda_0 \leq K_\lambda \\
&\quad \sigma = a_T + Y_\sigma a_E;\ A\Delta m \leq B a_E;\ m = m_0 + Z_m \Delta m;\ J_m m_0 \leq K_m \\
&\quad \bar{m} = \bar{\mu}_P + Y_m \bar{\mu}_E;\ C\Delta\bar{\lambda} \leq D\bar{\mu}_E;\ \bar{\lambda} = \lambda_0 + Z_\lambda \Delta\bar{\lambda} \\
&\quad \Delta\sigma = \Delta\bar{\lambda}\theta;\ \sigma = \lambda_0\theta + Z_\lambda \Delta\sigma \\
&\quad \bar{\sigma} = \frac{1}{2}\lambda_0\theta + Z_\lambda \Delta\bar{\sigma} \\
&\quad \bar{\sigma} = \bar{a}_T + Y_\sigma \bar{a}_E;\ A\Delta\bar{m} \leq B\bar{a}_E;\ \bar{m} = m_0 + Z_m \Delta\bar{m} \\
&\quad \bar{a}_E \leq a_E;\ \Delta\bar{m} \leq \Delta m;\ \Delta\bar{\sigma} \leq \Delta\sigma;\ \bar{\sigma} \leq \sigma \\
&\quad E_c \Delta\sigma = \theta F_c;\ E_c \Delta\bar{\sigma} = \frac{1}{2} E_c \Delta\sigma \\
&\quad E\Delta\sigma \leq \theta F \\
&\quad \theta E \Delta\bar{\sigma} \leq \frac{1}{2}(U\theta^2 - (U\theta - E\Delta\sigma) G (U\theta - E\Delta\sigma)) \\
&\quad \frac{1}{2}(M\theta^2 + (E\Delta\sigma - L\theta) G (E\Delta\sigma - L\theta)) + \theta N \Delta\sigma \leq \theta E \Delta\bar{\sigma} \\
&\quad a_T[t_j] = 0 \ \ \forall\, t_j \in T_F;\ \bar{a}_T[t_j] = 0 \ \ \forall\, t_j \in T_{Av} \\
&\quad \mu_P[p_i] = 0 \ \ \forall\, p_i \in P_F;\ \bar{\mu}_P[p_i] = 0 \ \ \forall\, p_i \in P_{Av}\}
\end{aligned} \tag{44}$$

where every variable is nonnegative.

3 Guarded flexible net. Definition

The intensity net proposed can model intensity uncertainties by means of intervals that depend linearly on the marking. Although this might be useful to approximate or abstract some nonlinear dynamics, it cannot model truly intensities that depend nonlinearly on the marking. This section proposes to better approximate nonlinear dynamics by associating a set of guards with each intensity arc. In this way, the intensities produced in arcs depend on the guard being active. More precisely, a guard will correspond to a region of the state space. A guard is said to be active if the net state is in the region corresponding to the guard. This section first introduces some preliminary concepts, and then defines formally guarded nets.

3.1 Guarded intensity arcs and state

Roughly, a guarded flexible net, denoted $\mathcal{N}_G$, is a flexible net such that each intensity arc $e \in E_S^T$ is assigned a set of guards. The intensity in e depends on the guard that is active, if no guard is active then the intensity in e is 0. To model this behavior, two different vectors are considered for the intensity of arcs: the vector $\Delta\lambda_U(\tau)$ contains all the potential intensities that the arcs can get, i.e., one value for each guard associated with the arc; the vector $\Delta\lambda(\tau)$ represents the actual intensity in arcs after the guards are applied. This way, an element of $\Delta\lambda_U$ corresponds to an arc $e \in E_S^T$ and a guard of e, thus, $\Delta\lambda_U$ is indexed by the pairs (e, r) where $e \in E_S^T$ and r is a guard of e. As in the previous section, vector $\Delta\lambda$ is indexed by $e \in E_S^T$.

In a guarded net, matrices A and B are defined as in non-guarded nets, and matrices C and D are used to determine $\Delta\lambda_U$. More precisely, the value of $\Delta\lambda_U$ can be determined by $C\Delta\lambda_U \leq D\mu_E$, where C and D are defined in such a way that every pair (e, r) is taken into account. This can be done by defining two matrices C_{lr} and D_{lr} for each guard r of the intensity arcs connected to handler s_l, such that the intensities of the arcs guarded by r and connected to s_l are given by $\Delta\lambda_{Uwr}$ that satisfies $C_{lr}\Delta\lambda_{Uwr} \leq D_{lr}\mu_w$, where μ_w is indexed by ${}^p s_l$ (see (11)). Matrix $C(D)$ can be built by arranging matrices $C_{lr}(D_{lr})$ diagonally(vertically) so that each column of $C(D)$ corresponds to a pair (e, r) (to an edge in E_S^P).

Thus, in addition to the already defined variables, $\Delta\lambda_U$ is included in the set of variables defining the state of a guarded net, i.e., the state is given by $\boldsymbol{x} = (m, \mu_P, \mu_E, \Delta\lambda_U, \Delta\lambda, \lambda, \Delta\sigma, \sigma, a_T, a_E, \Delta m)$. Notice that, even if the system can only be in some regions at a given time instant, $\Delta\lambda_U$ contains intensities corresponding to all regions. Consequently, the values of $\Delta\lambda_U$ corresponding to regions not containing the state of the system (such values do not affect the system evolution) might be negative. Hence, $\Delta\lambda_U$ will not be constrained to the nonnegative numbers.

As in (20), it is possible to establish a polytope, expressed as a set of linear constraints, that represents a necessary condition for reachability at any time. Such a polytope will be partitioned into regions which will be used as guards. Thus, as the value of $\Delta\lambda$, and in turn λ, depend on the region at which the state lies, $\Delta\lambda$ and λ are excluded from the set of constraints that define the polytope.

Proposition 7 (Untimed state equations (guarded net)) *Let $\mathcal{N}_G$ be a guarded flexible net with initial marking m_0 satisfying $J_m m_0 \leq K_m$. Every state $(m, \mu_P, \mu_E, \Delta\lambda_U, \Delta\lambda, \lambda, \Delta\sigma, \sigma, a_T, a_E, \Delta m)$ reachable at time τ belongs to $UGSE_{\mathcal{N}_G}(J_m, K_m, J_\lambda, K_\lambda)$ where:*

$$\begin{aligned} UGSE_{\mathcal{N}_G}(J_m, K_m, J_\lambda, K_\lambda) = \{&(m, \mu_P, \mu_E, \Delta\lambda_U, \Delta\lambda, \lambda, \Delta\sigma, \sigma, a_T, a_E, \Delta m)| \\ &m = \mu_P + Y_m\mu_E;\ C\Delta\lambda_U \leq D\mu_E \\ &\sigma = a_T + Y_\sigma a_E;\ A\Delta m \leq Ba_E;\ m = m_0 + Z_m\Delta m;\ J_m m_0 \leq K_m \\ &a_T[t_j] = 0\ \ \forall\ t_j \in T_F;\ \mu_P[p_i] = 0\ \ \forall\ p_i \in P_F\} \end{aligned} \quad (45)$$

where every variable, except $\Delta\lambda_U$, is nonnegative.

3.2 Partitions and regions

In order to establish guards on the intensity arcs, the state space, and hence $UGSE_{\mathcal{N}_G}$, is partitioned into several regions. In order to facilitate the task of defining the regions, a set of partitions is considered (defining several partitions instead of one can result in a lower number of regions and consequently in a decreased computational burden).

More formally, let $\mathcal{P} = \{\mathcal{P}_1, \ldots, \mathcal{P}_n, \ldots\}$ be the set of partitions. Each $\mathcal{P}_n$ partitions the state space into a set of regions. Thus, $\mathcal{P}_n$ is a set of regions $\mathcal{P}_n = \{\mathcal{R}_1, \ldots, \mathcal{R}_r, \ldots\}$ such that:

- $UGSE_{\mathcal{N}_G}$ is contained in the union of the regions, i.e., $UGSE_{\mathcal{N}_G} \subseteq \bigcup_{\mathcal{R}_r \in \mathcal{P}_n} \mathcal{R}_r$
- The regions are disjoint, i.e., $\mathcal{R}_r \cap \mathcal{R}_s = \emptyset$ for every pair of regions $\mathcal{R}_r, \mathcal{R}_s \in \mathcal{P}_n$.

Notice that the sets $\{\mathcal{P}_1, \ldots, \mathcal{P}_n, \ldots\}$ do not need to be disjoint, i.e., partitions can share regions . The set of all regions is denoted $\mathcal{R} = \bigcup_{\mathcal{P}_n \in \mathcal{P}} \bigcup_{\mathcal{R}_r \in \mathcal{P}_n} \mathcal{R}_r$. In the following, it will be assumed that each region $\mathcal{R}_r$ is a convex polytope of the form $\mathcal{R}_r = \{\boldsymbol{x} \mid S_r\boldsymbol{x} \leq Q_r\}$ where $S_r(Q_r)$ is a real valued matrix(vector), i.e., regions are assumed to have flat facets. In order to facilitate the analysis, it will also be assumed that the polytopes partition the real space $\mathbb{R}^{|x|}$. The previous expression to define polytopes uses non-strict inequalities ($S_r\boldsymbol{x} \leq Q_r$), i.e., the polytope is closed. This can involve a non-null intersection of polytopes at the borders. When the net state is at a shared border, it will be assumed to be in only one of the regions (any of them) sharing that border. This avoids the burden of having to define open polytopes so that their intersection is null.

Remark that the values of the intensity variables $\Delta\lambda_U$, $\Delta\lambda$ and λ depend on the location of the state in the regions. Hence, such variables should not be used to define the borders of the regions. Thus, it will be assumed that for each region $\mathcal{R}_r$, the columns of S_r that correspond to $\Delta\lambda_U$, $\Delta\lambda$ and λ are 0.

Although the set of partitions $\mathcal{P}$ is not used to define the system dynamics, it is useful to derive algebraic constraints that bound the potentially reachable states.

Let us define a binary variable $\delta_r(\tau)$ associated with each region $\mathcal{R}_r$ that

indicates whether the state is in $\mathcal{R}_r$:

$$\delta_r(\tau) = \begin{cases} 1 & \text{if } \boldsymbol{x}(\tau) \in \mathcal{R}_r \\ 0 & \text{otherwise} \end{cases} \tag{46}$$

Given that each $\mathcal{P}_n$ is a partition of the state space, the state is always in one and only one region in $\mathcal{P}_n$:

$$\sum_{\mathcal{R}_r \in \mathcal{P}_n} \delta_r(\tau) = 1 \ \ \forall\, \mathcal{P}_n \in \mathcal{P} \tag{47}$$

Equation (47) together with the following implication:

$$\delta_r(\tau) = 1 \ \rightarrow \ S_r \boldsymbol{x}(\tau) \leq Q_r \tag{48}$$

can be used to algebraically encode the definition of $\delta_r(\tau)$ in (46) (notice that when the state is in a shared border the $\delta_r(\tau)$ of any region containing that border can be equal to 1). Such an algebraic encoding can be done by substituting the implication (48) by the equivalent inequality:

$$S_r \boldsymbol{x}(\tau) \leq Q_r + W(1 - \delta_r(\tau)) \tag{49}$$

where W is a vector such that $W \geq S_r \boldsymbol{x} - Q_r$ for every reachable state $\boldsymbol{x}$ (see Appendix C for methods to compute W).

3.3 Definition and state equations

Each intensity arc $e \in E_S^T$ is assigned a set of regions through the function $\varphi : E_S^T \rightarrow 2^{\mathcal{R}}$. Each region $\mathcal{R}_r \in \varphi(e)$ is a guard of e that is denoted as r. The guard r is active when the state is in $\mathcal{R}_r$. It is assumed that the regions in $\varphi(e)$ are disjoint, i.e., $\mathcal{R}_r \cap \mathcal{R}_s = \emptyset$ for every pair of regions $\mathcal{R}_r, \mathcal{R}_s \in \varphi(e)$. Therefore, at most one guard of a given intensity arc is active at any given time. If no guard is active then the intensity in the intensity arc is 0.

A guarded flexible net is defined as a flexible net together with a set of partitions $\mathcal{P}$ and a function φ.

Definition 9 (Guarded flexible net) *A guarded flexible net is a tuple $\mathcal{N}_G = (P, T, V, E_V, A, B, S, E_S, C, D, \mathcal{P}, \varphi)$ where $(P, T, V, E_V, A, B, S, E_S, C, D)$ is a flexible net, $\mathcal{P}$ is a set of partitions and φ is a function that associates regions with intensity arcs.*

Let $e \in E_S^T$ and $\mathcal{R}_r \in \varphi(e)$, if the state is in $\mathcal{R}_r$ then the intensity $\Delta\lambda[e](\tau)$ is $\Delta\lambda_U[(e, r)](\tau)$. Thus, $\Delta\lambda$ can be expressed in matrix form as:

$$\Delta\lambda(\tau) = \delta(\tau)\Delta\lambda_U(\tau) \tag{50}$$

where $\delta[e, (e, r)](\tau) = \delta_r(\tau)$ (the pair (e, r) is the index of the column associated with the guard r of e) for every $e \in E_S^T$ and every $\mathcal{R}_r \in \varphi(e)$, and the rest of elements of δ are 0. Equation (50) is used in the following to establish the state equations of the guarded net.

Proposition 8 (State equations (guarded net)) *Let $\mathcal{N}_G$ be a guarded flexible net with initial marking m_0 satisfying $J_m m_0 \leq K_m$, and default intensities λ_0 satisfying $J_\lambda \lambda_0 \leq K_\lambda$. Every state $(m, \mu_P, \mu_E, \Delta\lambda_U, \Delta\lambda, \lambda, \Delta\sigma, \sigma, a_T, a_E, \Delta m)$ reachable at time τ belongs to $SE_{\mathcal{N}_G}(\tau, J_m, K_m, J_\lambda, K_\lambda)$ where:*

$$
\begin{aligned}
&GSE_{\mathcal{N}_G}(\tau, J_m, K_m, J_\lambda, K_\lambda) = \{(m, \mu_P, \mu_E, \Delta\lambda_U, \Delta\lambda, \lambda, \Delta\sigma, \sigma, a_T, a_E, \Delta m)| \\
&\quad m = \mu_P + Y_m \mu_E;\ C\Delta\lambda_U \leq D\mu_E;\ \Delta\lambda = \delta\Delta\lambda_U; \lambda = \lambda_0 + Z_\lambda \Delta\lambda;\ J_\lambda \lambda_0 \leq K_\lambda \\
&\quad \Delta\sigma = \int_0^\tau \Delta\lambda(s)\,\mathrm{d}s;\ \sigma = \lambda_0 \tau + Z_\lambda \Delta\sigma \\
&\quad \sigma = a_T + Y_\sigma a_E;\ A\Delta m \leq B a_E;\ m = m_0 + Z_m \Delta m;\ J_m m_0 \leq K_m\}
\end{aligned}
\tag{51}
$$

where every variable, except $\Delta\lambda_U$, is nonnegative.

By making use of (50), (47) and (49), the untimed state equations in (45) can be improved as:

$$
\begin{aligned}
&UGSE_{\mathcal{N}_G}(J_m, K_m, J_\lambda, K_\lambda) = \{(m, \mu_P, \mu_E, \Delta\lambda_U, \Delta\lambda, \lambda, \Delta\sigma, \sigma, a_T, a_E, \Delta m)| \\
&\quad m = \mu_P + Y_m \mu_E;\ C\Delta\lambda_U \leq D\mu_E;\ \Delta\lambda = \delta\Delta\lambda_U; \lambda = \lambda_0 + Z_\lambda \Delta\lambda;\ J_\lambda \lambda_0 \leq K_\lambda \\
&\quad \sigma = a_T + Y_\sigma a_E;\ A\Delta m \leq B a_E;\ m = m_0 + Z_m \Delta m;\ J_m m_0 \leq K_m \\
&\quad \text{for every } \mathcal{R}_r \in \mathcal{R} \\
&\qquad\quad S_r \boldsymbol{x} \leq Q_r + W(1 - \delta_r) \\
&\qquad\quad \text{for every } e \in E_S^T \text{ such that } \mathcal{R}_r \in \varphi(e) \\
&\qquad\qquad\quad \delta[e, (e, r)] = \delta_r \\
&\qquad\qquad\quad \text{for every } e' \in E_S^T \text{ such that } e' \neq e \\
&\qquad\qquad\qquad\quad \delta[e', (e, r)] = 0 \\
&\quad \text{for every } \mathcal{P}_n \in \mathcal{P} \\
&\qquad\quad \sum_{\mathcal{R}_r \in \mathcal{P}_n} \delta_r = 1 \\
&\quad a_T[t_j] = 0\ \ \forall\, t_j \in T_F;\ \mu_P[p_i] = 0\ \ \forall\, p_i \in P_F\}
\end{aligned}
\tag{52}
$$

where every δ_r is a binary variable $\{0, 1\}$. It must be remarked that although the equation $\Delta\lambda = \delta\Delta\lambda_U$ is not linear, it can be linearized as described in Appendix B.

Notice that guards can be used to easily *silence* intensity arcs, and therefore to stop the production of actions in transitions, at desired regions. Thus, as a transition without actions does not play any role in the system dynamics, a particular use of guards is to model a state dependent net structure.

3.4 Modeling examples

Guards can be used, in particular, to model discontinuities in the intensities produced. As an example, by using guards, inhibition and activation can be modeled. Let us show how to model an intensity handler that produces intensities only when a given marking is below a given threshold z, see Figure 5 a). This can be achieved by associating the following equations (which define

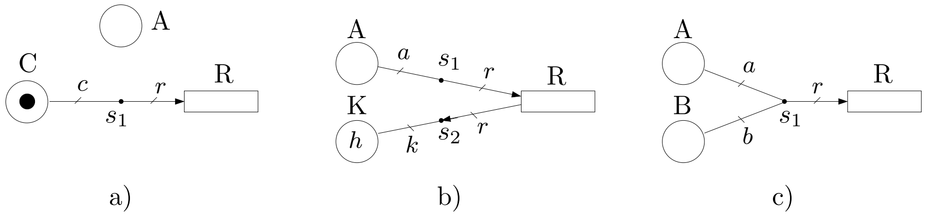

Figure 5: Guarded flexible net. Modeling examples.

matrices C and D) with the intensity handler s_1:

$$s_1 : \begin{cases} r = c & \text{if } \; m[A] \leq z \\ r = 0 & \text{if } \; m[A] \geq z \end{cases} \tag{53}$$

and forcing the tokens in C to be active, see Subsection 1.2.4. This way, the intensity produced in R is equal to the marking in C if the marking of A is lower than z, and 0 otherwise. Notice that the above equations make use of a partition of the state space with two regions defined as $m[A] \leq z$ and $m[A] \geq z$, only the first one is used as a guard of (s_1, R). Notice also that place A is not connected to s_1 as its marking is just used to define the regions and not to produce intensities.

Let us assume that the intensity of a transition is equal to a given marking $m[A]$ up to a certain threshold h and then it is equal to $2m[A]-h$. This nonlinear relationship is modeled by the net in Figure 5 b), where the marking of place K is constant and equal to h, the tokens of both places are forced to be active, and the guards and equations associated with the net elements are:

$$s_1 : \begin{cases} r = a & \text{if } \; \mu_E[\{A, s_1\}] \leq h \\ r = 2a & \text{if } \; \mu_E[\{A, s_1\}] \geq h \end{cases} \tag{54}$$

$$s_2 : \begin{cases} r = 0 & \text{if } \; \mu_E[\{A, s_1\}] \leq h \\ r = k & \text{if } \; \mu_E[\{A, s_1\}] \geq h \end{cases} \tag{55}$$

In this example, (s_1, R) has two guards and (s_2, R) just one.

Guards can also be used to produce intensities that are proportional to the minimum marking of a set of places, i.e., proportional to the limiting factor. In terms of queueing theory, this corresponds to infinitely many servers (the case of single server can be modeled by a non-null default intensity λ_0). Consider the net in Figure 5 c), and assume that the tokens in places A and B are forced to be active. The intensity produced in R can be made equal to the minimum number of tokens in $\mu_E[\{A, s_1\}]$ and $\mu_E[\{B, s_2\}]$ by associating the following equations with s_1:

$$s_1 : \begin{cases} r = a & \text{if } \; \mu_E[\{A, s_1\}] \leq \mu_E[\{B, s_1\}] \\ r = b & \text{if } \; \mu_E[\{B, s_1\}] \leq \mu_E[\{A, s_1\}] \end{cases} \tag{56}$$

Accurate models can be obtained by using guards, however the analysis of a net with a high number of regions might be computationally expensive. Regions can be merged by bounding the intensities that are produced in order to decrease

the number of regions. As an example, the two regions just proposed for the net in Figure 5 c) could be merged in a single one with associated equations $r \leq a; r \leq b$. The resulting net is not guarded but the produced intensities do not need to be equal, but just less that or equal, to the minimum of $\mu_E[\{A, s_1\}]$ and $\mu_E[\{B, s_1\}]$. Such intensities can also be achieved if $r = a = b$ is associated with s_1 and tokens are not forced to be active. In this latter case, the number of active tokens in $\{A, s_1\}$ and $\{B, s_1\}$ must be the same, i.e., $\mu_E[\{A, s_1\}] = \mu_E[\{B, s_1\}]$, and the idle tokens in A and B could be used to produce intensities in other transitions.

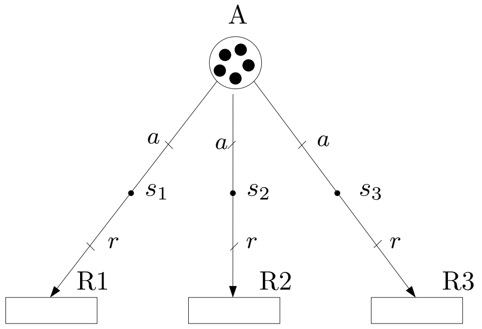

Figure 6: Guarded flexible net.

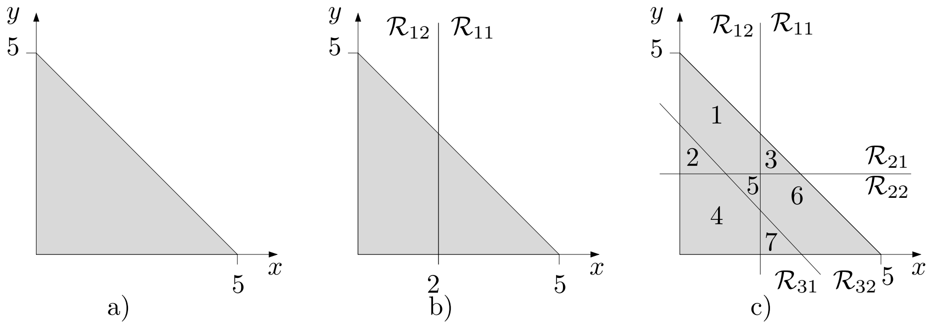

Figure 7: Regions associated with the guards of Figure 6, x denotes $\mu_E[\{A, s_1\}]$ and y denotes $\mu_E[\{A, s_2\}]$.

Let us consider the net in Figure 6 to show how regions can be split to obtain a single partition. Let the initial set of partitions be $\mathcal{P} = \{\mathcal{P}_1, \mathcal{P}_2, \mathcal{P}_3\}$ and the regions be $\mathcal{P}_1 = \{\mathcal{R}_{11}, \mathcal{R}_{12}\}$, $\mathcal{P}_2 = \{\mathcal{R}_{21}, \mathcal{R}_{22}\}$ and $\mathcal{P}_3 = \{\mathcal{R}_{31}, \mathcal{R}_{32}\}$. Let the regions be defined as: $\mathcal{R}_{11} : \mu_E[\{A, s_1\}] \geq 2$, $\mathcal{R}_{12} : \mu_E[\{A, s_1\}] \leq 2$, $\mathcal{R}_{21} : \mu_E[\{A, s_2\}] \geq 2$, $\mathcal{R}_{22} : \mu_E[\{A, s_2\}] \leq 2$ and $\mathcal{R}_{31} : \mu_E[\{A, s_3\}] \geq 2$, $\mathcal{R}_{32} : \mu_E[\{A, s_3\}] \leq 2$. Let us associate the following equations with the intensity handlers:

$$s_1 : \begin{cases} r = a & \text{if } \boldsymbol{x} \in \mathcal{R}_{11} \\ r = 0 & \text{if } \boldsymbol{x} \in \mathcal{R}_{12} \end{cases} \tag{57}$$

$$s_2 : \begin{cases} r = a & \text{if } \boldsymbol{x} \in \mathcal{R}_{21} \\ r = 0 & \text{if } \boldsymbol{x} \in \mathcal{R}_{22} \end{cases} \tag{58}$$

$$s_3 : \begin{cases} r = a & \text{if } \boldsymbol{x} \in \mathcal{R}_{31} \\ r = 0 & \text{if } \boldsymbol{x} \in \mathcal{R}_{32} \end{cases} \tag{59}$$

Given that the marking of A is constant and equal to 5, the following conservation law holds $m[A] = \mu_P[A] + \mu_E[\{A, s_1\}] + \mu_E[\{A, s_2\}] + \mu_E[\{A, s_3\}] = 5$. Let us assume that the tokens in A are forced to be active, see Subsection 1.2.4, then $\mu_E[\{A, s_1\}] + \mu_E[\{A, s_2\}] + \mu_E[\{A, s_3\}] = 5$. Thus, the potential values of $\mu_E[\{A, s_1\}]$, $\mu_E[\{A, s_2\}]$ and $\mu_E[\{A, s_3\}]$ can be depicted in a two dimensional plane, see the gray area in Figure 7 a) where x denotes $\mu_E[\{A, s_1\}]$ and y denotes $\mu_E[\{A, s_2\}]$. Each partition partitions the state space in two regions, see Figure 7 b) for the regions of $\mathcal{P}_1$.

In order to obtain a net with equivalent dynamics and just one partition, each region of a given partition can be split according to the borders that define the regions in the other partitions. In other words, each resulting region is the intersection of $|\mathcal{P}|$ regions, one region per partition. Thus, the number of resulting regions is upper bounded by $\prod_{\mathcal{P}_n \in \mathcal{P}} |\mathcal{P}_n|$. Nevertheless, the actual number of resulting regions can be lower as some intersections can be empty.

The regions obtained after intersecting every possible combination of the previously defined regions are shown in Figure 7 c) (the number i denotes the region $\mathcal{R}_i$). Notice that, although the potential number of resulting regions is 8, one of them (namely $\mathcal{R}_{11} \cap \mathcal{R}_{21} \cap \mathcal{R}_{31}$) is empty, i.e., non reachable, due to the mentioned conservation law.

The equations associated with s_1 in the obtained net are:

$$s_1 : \begin{cases} r = 0 & \text{if } \boldsymbol{x} \in \mathcal{R}_1 \\ r = 0 & \text{if } \boldsymbol{x} \in \mathcal{R}_2 \\ r = a & \text{if } \boldsymbol{x} \in \mathcal{R}_3 \\ r = 0 & \text{if } \boldsymbol{x} \in \mathcal{R}_4 \\ r = 0 & \text{if } \boldsymbol{x} \in \mathcal{R}_5 \\ r = a & \text{if } \boldsymbol{x} \in \mathcal{R}_6 \\ r = a & \text{if } \boldsymbol{x} \in \mathcal{R}_7 \end{cases} \tag{60}$$

where, as an example, $\mathcal{R}_1$ is determined by the inequalities: $\mu_E[\{A, s_1\}] \leq 2; \mu_E[\{A, s_2\}] \geq 2; \mu_E[\{A, s_3\}] \leq 2$. The equations for the s_2 and s_3 can be obtained in a similar way.

Other type of behaviors can be modeled by defining regions on other state variables different than μ_E. For instance, by defining a region in terms of a_E it is possible to stop producing intensity in a given transition, i.e., disabling it, once it has executed more actions than a given threshold.

4 Guarded flexible net. Bounds

This section obtains a set of constraints that represent necessary reachability conditions in guarded nets. Similarly to non-guarded nets (see Section 2), such constraints will be obtained by computing average values. However, in addition to average values computed for the non guarded net, average values of the state variables in each region will be computed.

Subsection 4.1 defines the ratio of time spent by the net at each region. Time ratios are used in Subsection 4.2 to introduce the concept of average state in a region. Subsection 4.3 focuses on the average state variables in regions, and establishes relationships among them by integrating the state equations (51). In a similar way, Subsection 4.4 focuses on the overall average state variables, and obtains expressions for them by integrating the state equations (51). The expressions obtained in Subsections 4.3 and 4.4 are the basis for the reachability conditions in Subsection 4.5.

4.1 Time ratios

The time ratio spent by the net in $\mathcal{R}_r$ during the first θ time units is defined as:

$$\bar{\delta}_r = \frac{1}{\theta}\int_0^\theta \delta_r(\tau)\,\mathrm{d}\tau \tag{61}$$

Thus, by (47), the sum of the ratios of the regions in a partition is equal to 1:

$$\sum_{\mathcal{R}_r\in\mathcal{P}_n} \bar{\delta}_r = \sum_{\mathcal{R}_r\in\mathcal{P}_n} \frac{1}{\theta}\int_0^\theta \delta_r(\tau)\,\mathrm{d}\tau = 1 \quad \forall\ \mathcal{P}_n \in \mathcal{P} \tag{62}$$

Notice that if a given region $\mathcal{R}_r$ is contained in a region $\mathcal{R}_s$, i.e., $\mathcal{R}_r \subseteq \mathcal{R}_s$, then it necessarily holds $\bar{\delta}_r \leq \bar{\delta}_s$. This idea can be extended to sets of regions. Let $\mathcal{V}$ and $\mathcal{W}$ be the spaces defined as the union of a set of disjoint regions, e.g., $\mathcal{V} = \ldots \cup \mathcal{R}_r \cup \ldots$, and $\mathcal{W} = \ldots \cup \mathcal{R}_s \cup \ldots$. Then:

$$\sum_{\mathcal{R}_r\in\mathcal{V}} \bar{\delta}_r \leq \sum_{\mathcal{R}_s\in\mathcal{W}} \bar{\delta}_s \quad \text{if } \mathcal{V} \subseteq \mathcal{W} \tag{63}$$

what implies:

$$\sum_{\mathcal{R}_r\in\mathcal{V}} \bar{\delta}_r = \sum_{\mathcal{R}_s\in\mathcal{W}} \bar{\delta}_s \quad \text{if } \mathcal{V} = \mathcal{W} \tag{64}$$

4.2 Average state in regions

The average state over the interval $[0,\theta]$ when the net is in region $\mathcal{R}_r$ is defined as:

$$\bar{\boldsymbol{x}}_r = \frac{1}{\int_0^\theta \delta_r(\tau)\,\mathrm{d}\tau}\int_0^\theta \delta_r(\tau)\boldsymbol{x}(\tau)\,\mathrm{d}\tau = \frac{1}{\bar{\delta}_r\theta}\int_0^\theta \delta_r(\tau)\boldsymbol{x}(\tau)\,\mathrm{d}\tau \tag{65}$$

Notice that the average $\bar{\boldsymbol{x}}_r$ is computed on the time spent in $\mathcal{R}_r$, i.e., $\int_0^\theta \delta_r(\tau)\,\mathrm{d}\tau$. If the state never visits a region $\mathcal{R}_r$, then $\delta_r(\tau) = 0$ for every $\tau \in [0,\theta]$ and $\bar{\boldsymbol{x}}_r$ is indeterminate.

Let us define a binary variable $\alpha_r \in \{0,1\}$ that indicates if the region $\mathcal{R}_r$ has been visited:

$$\alpha_r = 0 \;\leftrightarrow\; \bar{\delta}_r = 0 \tag{66}$$

Expression (66) is difficult to translate and handle as a linear expression in an exact way. Thus, it will be relaxed to $\alpha_r = 0 \;\leftrightarrow\; \bar{\delta}_r \leq \epsilon$ where $\epsilon > 0$ is an arbitrarily small quantity, e.g., the computer precision. This expression is equivalent to:

$$\begin{aligned} \epsilon - \bar{\delta}_r &\leq 2(1-\alpha_r) \\ \epsilon - \bar{\delta}_r &\geq -2\alpha_r \end{aligned} \tag{67}$$

If $\mathcal{R}_r$ is visited, then by (65) it holds $\bar{\boldsymbol{x}}_r \in \mathcal{R}_r$, i.e., $\bar{\boldsymbol{x}}_r$ satisfies:

$$S_r \bar{\boldsymbol{x}}_r \leq Q_r \tag{68}$$

By using a similar approach as in (49), inequality (68) can be slightly modified as follows to take into account regions that are not visited or are not reachable:

$$S_r \bar{\boldsymbol{x}}_r \leq Q_r + W(1-\alpha_r) \tag{69}$$

Notice that $\boldsymbol{x}(\tau)$ can be expressed as $\boldsymbol{x}(\tau) = \sum\limits_{\mathcal{R}_r \in \mathcal{P}_n} \delta_r(\tau)\boldsymbol{x}(\tau)$ for every $\mathcal{P}_n \in \mathcal{P}$. Then, by (65), the average state $\bar{\boldsymbol{x}}$ can be expressed in terms of $\bar{\boldsymbol{x}}_r$ as:

$$\begin{aligned} \bar{\boldsymbol{x}} &= \frac{1}{\theta}\int_0^\theta \boldsymbol{x}(\tau)\,\mathrm{d}\tau = \frac{1}{\theta}\int_0^\theta \sum_{\mathcal{R}_r \in \mathcal{P}_n} \delta_r(\tau)\boldsymbol{x}(\tau)\,\mathrm{d}\tau \\ &= \frac{1}{\theta}\sum_{\mathcal{R}_r \in \mathcal{P}_n} \int_0^\theta \delta_r(\tau)\boldsymbol{x}_r(\tau)\,\mathrm{d}\tau = \sum_{\mathcal{R}_r \in \mathcal{P}_n} \bar{\delta}_r \bar{\boldsymbol{x}}_r \;\; \forall\, \mathcal{P}_n \in \mathcal{P} \end{aligned} \tag{70}$$

Moreover, if v is a nondecreasing state variable, i.e., if $v \in \{\Delta\sigma, \sigma, a_E, \Delta m\}$, then for every region $\mathcal{R}_r$ that has been visited it holds:

$$\bar{v}_r \leq v(\theta) \tag{71}$$

By using α_r and equation (71) the following inequalities are satisfied:

$$\alpha_r \bar{v}_r \leq v(\theta) \;\; \forall\, \mathcal{R}_r \in \mathcal{R} \tag{72}$$

On the other hand, a relationship among the average state in a set of regions can be established if $\mathcal{V} = \mathcal{W}$, where $\mathcal{V}$ and $\mathcal{W}$ are defined as in Subsection 4.1. Let us define $\bar{\sigma}_{\mathcal{V}}$ as:

$$\bar{\boldsymbol{x}}_{\mathcal{V}} = \frac{1}{\bar{\delta}_{\mathcal{V}}\theta}\int_0^\theta \delta_{\mathcal{V}}(\tau)\boldsymbol{x}(\tau)\,\mathrm{d}\tau \tag{73}$$

where $\delta_{\mathcal{V}} = \sum\limits_{\mathcal{R}_r \in \mathcal{V}} \delta_r$ and $\bar{\delta}_{\mathcal{V}} = \sum\limits_{\mathcal{R}_r \in \mathcal{V}} \bar{\delta}_r$. Recall that $\mathcal{V}$ and $\mathcal{W}$ are defined as the union of disjoint regions. Then, by (65):

$$\begin{aligned} \bar{\boldsymbol{x}}_{\mathcal{V}} &= \frac{1}{\sum\limits_{\mathcal{R}_r \in \mathcal{V}} \bar{\delta}_r\theta}\int_0^\theta \sum_{\mathcal{R}_r \in \mathcal{V}} \delta_r(\tau)\boldsymbol{x}(\tau)\,\mathrm{d}\tau \\ &= \frac{1}{\sum\limits_{\mathcal{R}_r \in \mathcal{V}} \bar{\delta}_r\theta}\sum_{\mathcal{R}_r \in \mathcal{V}}\int_0^\theta \delta_r\boldsymbol{x}(\tau)\,\mathrm{d}\tau = \frac{\sum\limits_{\mathcal{R}_r \in \mathcal{V}} \bar{\delta}_r\bar{\boldsymbol{x}}_r\theta}{\sum\limits_{\mathcal{R}_r \in \mathcal{V}} \bar{\delta}_r\theta} = \frac{\sum\limits_{\mathcal{R}_r \in \mathcal{V}} \bar{\delta}_r\bar{\boldsymbol{x}}_r}{\sum\limits_{\mathcal{R}_r \in \mathcal{V}} \bar{\delta}_r} \end{aligned} \tag{74}$$

If $\mathcal{V} = \mathcal{W}$ then it necessarily holds $\bar{\boldsymbol{x}}_{\mathcal{V}} = \bar{\boldsymbol{x}}_{\mathcal{W}}$, and by (74): $\frac{\sum_{\mathcal{R}_r \in \mathcal{V}} \bar{\delta}_r \bar{\boldsymbol{x}}_r}{\sum_{\mathcal{R}_r \in \mathcal{V}} \bar{\delta}_r} = \frac{\sum_{\mathcal{R}_s \in \mathcal{W}} \bar{\delta}_s \bar{\boldsymbol{x}}_s}{\sum_{\mathcal{R}_s \in \mathcal{W}} \bar{\delta}_s}$. Thus, by (64):

$$\sum_{\mathcal{R}_r \in \mathcal{V}} \bar{\delta}_r \bar{\boldsymbol{x}}_r = \sum_{\mathcal{R}_s \in \mathcal{W}} \bar{\delta}_s \bar{\boldsymbol{x}}_s \quad \text{if } \mathcal{V} = \mathcal{W} \tag{75}$$

4.3 Average state equations in regions

As in the non-guarded net, the equations in (51) will be integrated to obtain expressions for the average state. Such expressions will be used to obtain the number of produced actions, and in turn, the set of reachable states at time θ.

Let us first express the average of the state equations in the regions. Let us assume that $\mathcal{R}_r$ is visited. By integrating the linear equations: $m = \mu_P + Y_m \mu_E$, $C\Delta\lambda_U \leq D\mu_E$, $\lambda = \lambda_0 + Z_\lambda \Delta\lambda$, $\sigma = a_T + Y_\sigma a_E$, $A\Delta m \leq B a_E$ and $m = m_0 + Z_m$ as in (65), the following relationships between average values in regions are obtained:

$$\begin{aligned} \bar{m}_r = \bar{\mu}_{Pr} + Y_m \bar{\mu}_{Er};\ C\Delta\bar{\lambda}_{Ur} \leq D\bar{\mu}_{Er};\ \bar{\lambda}_r = \lambda_0 + Z_\lambda \Delta\bar{\lambda}_r \\ \bar{\sigma}_r = \bar{a}_{Tr} + Y_\sigma \bar{a}_{Er};\ A\Delta\bar{m}_r \leq B\bar{a}_{Er};\ \bar{m}_r = m_0 + Z_m \Delta\bar{m}_r \end{aligned} \tag{76}$$

Notice that if $\mathcal{R}_r \in \varphi(e)$ then $\Delta\bar{\lambda}_r[e] = \Delta\bar{\lambda}_{Ur}[(e,r)]$. That is, the value of $\Delta\bar{\lambda}_r$ can be easily related for those arcs that have $\mathcal{R}_r$ as guard:

$$\Delta\bar{\lambda}_r[e] = \Delta\bar{\lambda}_{Ur}[(e,r)] \ \ \forall\, e \in E_S^T,\ \forall\, \mathcal{R}_r \in \varphi(e) \tag{77}$$

If region $\mathcal{R}_r$ is not reachable (what would imply $\alpha_r = 0$) then $\Delta\bar{\lambda}_{Ur}[(e,r)]$ can be negative. In order to avoid assigning a negative value to $\Delta\bar{\lambda}_r[e]$, (77) can be replaced by:

$$\Delta\bar{\lambda}_r[e] = \alpha_r \Delta\bar{\lambda}_{Ur}[(e,r)] \ \ \forall\, e \in E_S^T,\ \forall\, \mathcal{R}_r \in \varphi(e) \tag{78}$$

Next subsection integrates the equation $\Delta\lambda = \delta\Delta\lambda_U$ to obtain $\Delta\bar{\lambda}$. The resulting expression makes use of $\Delta\bar{\lambda}_{Ur}$ which is determined in (76). The value of $\Delta\bar{\lambda}$ will then be used to compute $\Delta\sigma$, and in turn σ, which drives the net evolution.

4.4 Average state equations

In (51), every equation, except $\Delta\lambda = \delta\Delta\lambda_U$ and $\Delta\sigma = \int_0^\tau \Delta\lambda(s)\,\mathrm{d}s$ is linear. Let us integrate $\Delta\lambda = \delta\Delta\lambda_U$ over time to compute the average intensities $\Delta\bar{\lambda}$ in arcs. These will be used to obtain the number of actions produced. By (21) and $\Delta\lambda = \delta\Delta\lambda_U$:

$$\Delta\bar{\lambda} = \frac{1}{\theta}\int_0^\theta \delta(\tau)\Delta\lambda_U(\tau)\,\mathrm{d}\tau \tag{79}$$

Let us consider each component $\Delta\bar{\lambda}[e]$ of $\Delta\bar{\lambda}$ separately. Then, by the definition of δ, the fact that the regions in $\varphi(e)$ are disjoint and (65), $\Delta\bar{\lambda}[e]$ in (79) can

be expressed as:

$$\begin{aligned}\Delta\bar{\lambda}[e] &= \frac{1}{\theta}\int_0^\theta \sum_{\mathcal{R}_r\in\varphi(e)} \delta_r(\tau)\Delta\lambda_U[(e,r)](\tau)\,\mathrm{d}\tau \\ &= \sum_{\mathcal{R}_r\in\varphi(e)} \frac{1}{\theta}\int_0^\theta \delta_r(\tau)\Delta\lambda_U[(e,r)](\tau)\,\mathrm{d}\tau = \sum_{\mathcal{R}_r\in\varphi(e)} \bar{\delta}_r\Delta\bar{\lambda}_{Ur}[(e,r)]\end{aligned} \tag{80}$$

where the pair (e,r) indexes the element associated with the guard r of e. This can be expressed in matrix form as:

$$\Delta\bar{\lambda} = \bar{\delta}\Delta\bar{\lambda}_G \tag{81}$$

where $\bar{\delta}$ has the same size as δ, and it is defined as $\bar{\delta}[e,(e,r)] = \bar{\delta}_r$ for every $e \in E_S^T$ and every $\mathcal{R}_r \in \varphi(e)$, and the rest of elements of $\bar{\delta}$ are 0; and $\Delta\bar{\lambda}_G$ is indexed by (e,r) and it is defined as:

$$\Delta\bar{\lambda}_G[(e,r)] = \Delta\bar{\lambda}_{Ur}[(e,r)] \;\; \forall\, e\in E_S^T,\; \forall\, \mathcal{R}_r \in \varphi(e) \tag{82}$$

As in (78), in order to avoid assigning negative values to $\Delta\bar{\lambda}_G[(e,r)]$, (82) can be replaced by:

$$\Delta\bar{\lambda}_G[(e,r)] = \alpha_r\Delta\bar{\lambda}_{Ur}[(e,r)] \;\; \forall\, e\in E_S^T,\; \forall\, \mathcal{R}_r \in \varphi(e) \tag{83}$$

Thus, similarly to (22), the average values satisfy:

$$\begin{aligned}&\bar{m} = \bar{\mu}_P + Y_m\bar{\mu}_E;\; C\Delta\bar{\lambda}_U \le D\bar{\mu}_E;\; \Delta\bar{\lambda} = \bar{\delta}\Delta\bar{\lambda}_G;\; \bar{\lambda} = \lambda_0 + Z_\lambda\Delta\bar{\lambda};\; J_\lambda\lambda_0 \le K_\lambda \\ &\Delta\sigma = \Delta\bar{\lambda}\theta;\; \sigma = \lambda_0\theta + Z_\lambda\Delta\sigma \\ &\Delta\bar{\sigma} = \frac{1}{\theta}\int_0^\theta\int_0^\tau \Delta\lambda(s)\,\mathrm{d}s\,\mathrm{d}\tau;\; \bar{\sigma} = \frac{1}{2}\lambda_0\theta + Z_\lambda\Delta\bar{\sigma} \\ &\bar{\sigma} = \bar{a}_T + Y_\sigma\bar{a}_E;\; A\Delta\bar{m} \le B\bar{a}_E;\; \bar{m} = m_0 + Z_m\Delta\bar{m};\; J_m m_0 \le K_m\end{aligned} \tag{84}$$

4.5 Reachability conditions

The following sets of equations will be considered to define necessary reachability conditions for a guarded net at time θ: a) equations that relate the overall state variables: (51), (84), (23); b) equations that hold for each partition: (47), (62), (70); c) equations that hold for each region: (49), (69), (76), (78), (83), (67), (72); d) equations that hold for sets of regions: (63), (64), (75); e) equations that account for intensity bounds: (28), (29), (31), (39), (41).

Proposition 9 (Reachable states) *Let $\mathcal{N}_G$ be a guarded flexible net with initial marking m_0 satisfying $J_m m_0 \le K_m$, and default intensities λ_0 satisfying $J_\lambda\lambda_0 \le K_\lambda$. Every state $\boldsymbol{x} = (m, \mu_P, \mu_E, \Delta\lambda_U, \Delta\lambda, \lambda, \Delta\sigma, \sigma, a_T, a_E, \Delta m)$ reachable at time θ belongs to $TC_{\mathcal{N}_G}(\theta, J_m, K_m, J_\lambda, K_\lambda)$ where:*

$$
\begin{aligned}
&TC_{\mathcal{N}_G}(\theta, J_m, K_m, J_\lambda, K_\lambda) = \{\boldsymbol{x} = (m, \mu_P, \mu_E, \Delta\lambda_U, \Delta\lambda, \lambda, \Delta\sigma, \sigma, a_T, a_E, \Delta m) |\\
&\quad m = \mu_P + Y_m \mu_E;\ C\Delta\lambda_U \leq D\mu_E;\ \Delta\lambda = \delta\Delta\lambda_U; \lambda = \lambda_0 + Z_\lambda \Delta\lambda;\ J_\lambda \lambda_0 \leq K_\lambda\\
&\quad \sigma = a_T + Y_\sigma a_E;\ A\Delta m \leq B a_E;\ m = m_0 + Z_m \Delta m;\ J_m m_0 \leq K_m\\
&\quad \bar{m} = \bar{\mu}_P + Y_m \bar{\mu}_E;\ C\Delta\bar{\lambda}_U \leq D\bar{\mu}_E;\ \Delta\bar{\lambda} = \bar{\delta}\Delta\bar{\lambda}_G;\ \bar{\lambda} = \lambda_0 + Z_\lambda \Delta\bar{\lambda}\\
&\quad \Delta\sigma = \Delta\bar{\lambda}\theta;\ \sigma = \lambda_0\theta + Z_\lambda \Delta\sigma\\
&\quad \bar{\sigma} = \frac{1}{2}\lambda_0\theta + Z_\lambda \Delta\bar{\sigma}\\
&\quad \bar{\sigma} = \bar{a}_T + Y_\sigma \bar{a}_E;\ A\Delta\bar{m} \leq B\bar{a}_E;\ \bar{m} = m_0 + Z_m \Delta\bar{m}\\
&\quad \bar{a}_E \leq a_E;\ \Delta\bar{m} \leq \Delta m;\ \Delta\bar{\sigma} \leq \Delta\sigma;\ \bar{\sigma} \leq \sigma\\
&\quad E_c\Delta\sigma = \theta F_c;\ E_c\Delta\bar{\sigma} = \frac{1}{2}E_c\Delta\sigma\\
&\quad E\Delta\sigma \leq \theta F\\
&\quad \theta E\Delta\bar{\sigma} \leq \frac{1}{2}(U\theta^2 - (U\theta - E\Delta\sigma)G(U\theta - E\Delta\sigma))\\
&\quad \frac{1}{2}(M\theta^2 + (E\Delta\sigma - L\theta)G(E\Delta\sigma - L\theta)) + \theta N\Delta\sigma \leq \theta E\Delta\bar{\sigma}\\
&\quad \textit{for every } \mathcal{R}_r \in \mathcal{R}\\
&\qquad S_r\boldsymbol{x} \leq Q_r + W(1-\delta_r);\ S_r\bar{\boldsymbol{x}}_r \leq Q_r + W(1-\alpha_r)\\
&\qquad \bar{m}_r = \bar{\mu}_{Pr} + Y_m\bar{\mu}_{Er};\ C\Delta\bar{\lambda}_{Ur} \leq D\bar{\mu}_{Er}; \bar{\lambda}_r = \lambda_0 + Z_\lambda\Delta\bar{\lambda}_r\\
&\qquad \bar{\sigma}_r = \bar{a}_{Tr} + Y_\sigma\bar{a}_{Er};\ A\Delta\bar{m}_r \leq B\bar{a}_{Er};\ \bar{m}_r = m_0 + Z_m\Delta\bar{m}_r\\
&\qquad \epsilon - \bar{\delta}_r \leq 2(1-\alpha_r);\ \epsilon - \bar{\delta}_r \geq -2\alpha_r\\
&\qquad \alpha_r\bar{a}_{Er} \leq a_E;\ \alpha_r\Delta\bar{m}_r \leq \Delta m;\ \alpha_r\Delta\bar{\sigma}_r \leq \Delta\sigma;\ \alpha_r\bar{\sigma}_r \leq \sigma\\
&\qquad \textit{for every } e \in E_S^T \textit{ such that } \mathcal{R}_r \in \varphi(e)\\
&\qquad\quad \delta[e,(e,r)] = \delta_r;\ \bar{\delta}[e,(e,r)] = \bar{\delta}_r\\
&\qquad\quad \Delta\bar{\lambda}_G[(e,r)] = \alpha_r\Delta\bar{\lambda}_{Ur}[(e,r)];\ \Delta\bar{\lambda}_r[e] = \alpha_r\Delta\bar{\lambda}_{Ur}[(e,r)]\\
&\qquad\quad \textit{for every } e' \in E_S^T \textit{ such that } e' \neq e\\
&\qquad\qquad \delta[e',(e,r)] = 0;\ \bar{\delta}[e',(e,r)] = 0\\
&\quad \textit{for every } \mathcal{P}_n \in \mathcal{P}\\
&\qquad \sum_{\mathcal{R}_r \in \mathcal{P}_n} \delta_r = 1;\ \sum_{\mathcal{R}_r \in \mathcal{P}_n} \bar{\delta}_r = 1;\ \bar{\boldsymbol{x}} = \sum_{\mathcal{R}_r \in \mathcal{P}_n} \bar{\delta}_r\bar{\boldsymbol{x}}_r\\
&\quad \textit{for every } \mathcal{V}, \mathcal{W} \textit{ such that } \mathcal{V} \subseteq \mathcal{W}\\
&\qquad \sum_{\mathcal{R}_r \in \mathcal{V}} \bar{\delta}_r \leq \sum_{\mathcal{R}_s \in \mathcal{W}} \bar{\delta}_s\\
&\quad \textit{for every } \mathcal{V}', \mathcal{W}' \textit{ such that } \mathcal{V}' = \mathcal{W}'\\
&\qquad \sum_{\mathcal{R}_r \in \mathcal{V}'} \bar{\delta}_r = \sum_{\mathcal{R}_s \in \mathcal{W}'} \bar{\delta}_s;\ \sum_{\mathcal{R}_r \in \mathcal{V}'} \bar{\delta}_r\bar{\boldsymbol{x}}_r = \sum_{\mathcal{R}_s \in \mathcal{W}'} \bar{\delta}_s\bar{\boldsymbol{x}}_s\\
&\quad a_T[t_j] = 0\ \ \forall\ t_j \in T_F;\ \bar{a}_T[t_j] = 0\ \ \forall\ t_j \in T_{Av}\\
&\quad \mu_P[p_i] = 0\ \ \forall\ p_i \in P_F;\ \bar{\mu}_P[p_i] = 0\ \ \forall\ p_i \in P_{Av}\}
\end{aligned}
\tag{85}
$$

where every variable, except $\Delta\lambda_U$*,* $\Delta\bar{\lambda}_U$ *and* $\Delta\bar{\lambda}_{Ur}$*, is nonnegative, every* $\alpha_r \in \{0,1\}$ *and every* $\delta_r \in \{0,1\}$*.*

5 Intermediate states

The reachability conditions in the previous sections can be easily extended to consider several time intervals instead of just one. This can be useful to track the system state at different time instants, and allows the possibility of introducing time dependent parameters. In fact, the dynamics can be determined by different nets during each time interval.

Let $\theta_1, \ldots, \theta_q, \ldots, \theta_z$ be the time instants at which the system is to be tracked. It is assumed that $0<\theta_1<\theta_2<\ldots$ and for clarity θ_0 is defined as $\theta_0 = 0$. The guarded net determining the dynamics during the interval $q \geq 1$, which lasts from θ_{q-1} to θ_q, is denoted $\mathcal{N}_G^q$. Let $TC_{\mathcal{N}_G}(\theta, m_0, J_\lambda, K_\lambda)$ be a set containing the same equations than $TC_{\mathcal{N}_G}(\theta, J_m, K_m, J_\lambda, K_\lambda)$ except $J_m m_0 \leq K_m$.

The marking at the end of one interval is used to obtain the initial marking of the next interval. More precisely, in order to *link* the final marking, m^q, of a given interval q to the initial marking, m_0^{q+1}, of the next interval $q{+}1$, two matrices, In_m^{q+1} and Out_m^q, and the inequality $In_m^{q+1} m_0^{q+1} \leq Out_m^q m^q$ are used. The actions produced that have not been executed, i.e., variables a_T, are also transferred to the next interval so that they can be executed if required. They are transferred by means of matrices In_a^{q+1} and Out_a^q through the inequality $In_a^{q+1} a_0^{q+1} \leq Out_m^q a_T^q$, where a_0^{q+1} denotes available actions at the beginning of interval $q{+}1$. Thus a new vector of variables a_0^q indexed by transitions is included and it is assumed that $a_0^1 = 0$. In order to take into account a_0^q, all the occurrences of equations $\sigma = a_T + Y_\sigma a_E$, $\bar{\sigma} = \bar{a}_T + Y_\sigma \bar{a}_E$ and $\bar{\sigma}_r = \bar{a}_{Tr} + Y_\sigma \bar{a}_{Er}$ are substituted respectively by:

$$\sigma + a_0 = a_T + Y_\sigma a_E \tag{86}$$
$$\bar{\sigma} + a_0 = \bar{a}_T + Y_\sigma \bar{a}_E \tag{87}$$
$$\bar{\sigma}_r + a_0 = \bar{a}_{Tr} + Y_\sigma \bar{a}_{Er} \tag{88}$$

Notice that a nonzero value for a_0^1 could also be considered, such a value would represent the number of available actions at time 0. In such a case, the above substitutions would be required in the equations of this document (we have omitted them for the sake of simplicity). The rest of state variables will not be transferred from one interval to the next one. The state at the end of the qth interval is denoted $\boldsymbol{x}^q$.

Proposition 10 (Reachable states (intermediate states)) *Let $\Omega = (\theta_0, \ldots, \theta_q, \ldots, \theta_z)$ be a sequence of strictly increasing time instants, $SN = \{\mathcal{N}_G^1, \ldots, \mathcal{N}_G^q, \ldots, \mathcal{N}_G^z\}$ be a set of guarded flexible nets, $SJK = \{(J_\lambda^1, K_\lambda^1), \ldots, (J_\lambda^q, K_\lambda^q), \ldots, (J_\lambda^z, K_\lambda^z)\}$ contain the matrices constraining default intensities and $STR = \{\{\ldots, (In_m^q, Out_m^{q-1}), \ldots\}, \{\ldots, (In_a^q, Out_a^{q-1}), \ldots\}\}$ contain the matrices determining the transfer of tokens and actions between intervals. Let the initial marking m_0 satisfy $J_m m_0 \leq K_m$ and let the default intensities λ_0^q of $\mathcal{N}_G^q$ satisfy $J_\lambda^q \lambda_0 \leq K_\lambda^q$. Every state $\boldsymbol{x} = (m, \mu_P, \mu_E, \Delta\lambda_U, \Delta\lambda, \lambda, \Delta\sigma, \sigma, a_T, a_E, \Delta m)$ reachable at time θ_z belongs to $TC_{SN}(\Omega, J_m, K_m, SJK, STR)$ where:*

$$
\begin{aligned}
&TC_{SN}(\Omega, J_m, K_m, SJK, STR) = \\
&\quad \{\boldsymbol{x} = (m, \mu_P, \mu_E, \Delta\lambda_U, \Delta\lambda, \lambda, \Delta\sigma, \sigma, a_T, a_E, \Delta m) | \\
&\qquad \boldsymbol{x}^1 \in TC_{\mathcal{N}_G^1}(\theta_1 - \theta_0, J_m, K_m, J_\lambda^1, K_\lambda^1); \\
&\qquad In_m^2 m_0^2 \le Out_m^1 m^1; \; In_a^2 a_0^2 \le Out_a^1 a_T^1 \\
&\qquad \boldsymbol{x}^2 \in TC_{\mathcal{N}_G^2}(\theta_2 - \theta_1, m_0^2, J_\lambda^2, K_\lambda^2); \\
&\qquad In_m^3 m_0^3 \le Out_m^2 m^2; \; In_a^3 a_0^3 \le Out_a^2 a_T^2 \\
&\qquad \ldots \\
&\qquad \boldsymbol{x}^q \in TC_{\mathcal{N}_G^q}(\theta_q - \theta_{q-1}, m_0^q, J_\lambda^q, K_\lambda^q); \\
&\qquad In_m^{q+1} m_0^{q+1} \le Out_m^q m^q; \; In_a^{q+1} a_0^{q+1} \le Out_a^q a_T^q \\
&\qquad \ldots \\
&\qquad \boldsymbol{x} = \boldsymbol{x}^z \in TC_{\mathcal{N}_G^z}(\theta_z - \theta_{z-1}, m_0^z, J_\lambda^z, K_\lambda^z)\}
\end{aligned}
\tag{89}
$$

6 Optimization and control

6.1 Objective functions

Bounds for functions of interest can be computed by defining a programming problem that consists of a given objective function together with the obtained reachability conditions, either (24), (44), (85) or (89). Any variable in the conditions can be included in the objective function, e.g., $\boldsymbol{x}$, $\bar{\boldsymbol{x}}$, $\bar{\boldsymbol{x}}_r$, $\bar{\delta}_r$, α_r, etc.

The conditions in (24) together with an objective function result in a linear programming problem. Since matrix G in (44) is positive semidefinite, the constraints in (44) are convex and hence the result is a programming problem with convex quadratic constraints. Appendix B shows how the nonlinear multiplications in (85), and (89) can be approximated by linear ones by means of binary variables, what yields a programming problem that contains both real and binary variables.

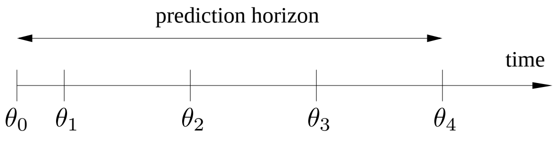

Figure 8: Intervals of different length for model predictive control.

A model predictive control [3] approach can be easily adopted to control flexible nets with intermediate states. Given that the time instants $\Omega = (\theta_0, \ldots, \theta_q, \ldots, \theta_z)$ need not to be evenly separated, intervals (or sample times) of different length can be considered to predict the future system evolution. For instance, one might desire the first interval to be short and the remaining intervals to be longer, see Figure 8. This way, the control actions are allowed to change rapidly (as they are applied during the first time interval only), and the system evolution can be predicted for a long time without having to make use of too many intervals. This long prediction horizon might help to prevent the optimization from falling in local minima. The resulting optimization variables provide a guideline for the controller to steer the system

towards the value of the objective function, e.g., λ_0 determines the values for the default intensities, μ_E for the distribution of tokens over edges, etc. The actual control actions to be implemented will depend on the capability of the controller to reduce the nondeterminism of the system, e.g., the capability to set desired default intensities or to distribute tokens over edges.

6.2 Model accuracy and computational complexity

The problems obtained from (44), (85) and (89) are nonlinear as they contain binary variables and multiplications of variables, being the number of binary variables and multiplications dependent on the number of regions. As shown in Appendix B, these multiplications can be relaxed as a set of linear inequalities with binary variables.

Notice that the dynamics of any guarded flexible net can be mimicked by another guarded flexible net with just one partition, see Subsection 3.4. This can be achieved by splitting each region into a set of regions and updating the set of guards of intensity arcs accordingly. As a result, the necessary reachability conditions proposed in the previous sections might be more accurate. Unfortunately, the number of regions required if only one partition is desired could be very high, what could involve a prohibitive computational cost.

Conversely, neighbor regions can be merged into a single region that abstracts their dynamic behavior by bounding the intensities that can be produced. This can result in a less accurate model that requires a lower computational cost to be analyzed, as less regions have to be handled. If all regions are merged, then the reachability conditions contain no binary variables. As a final relaxation step, the quadratic terms in the conditions can be omitted what would lead to a set of linear inequalities which can be optimized in polynomial time by solving a linear programming problem.

Thus, the trade-off accuracy of the model vs. computational cost can be selected by partitioning the state space appropriately.

A Intensity bounds

The inequality $E\Delta\lambda \leq F$ (Subsection 2.2) can be used by the modeler either to establish a desired constraint on the intensities, or to further constrain the potential states that the system can reach and hence to improve the optimization results. In order to make clear these two different roles, matrices E and F will be divided into two sub-matrices, E^a and E^u, F^a and F^u, such that the inequality $E^u\Delta\lambda \leq F^u$ contains the constraints imposed by the modeler, and $E^a\Delta\lambda \leq F^a$ is used to improve the accuracy of the results. We will assume that F^u is provided by the modeler, and F^a has to be computed.

This section proposes a method to compute F^a provided that E^a satisfies some assumptions. First, an algorithm to compute F^a for a given time interval $[0, \theta]$ is designed, and then, such an algorithm is extended to take into account intermediate states.

Algorithm 1 produces F^a under the following assumptions: a) all the rows of E^a have one and only one nonzero component, i.e., each row of E^a corresponds to the intensity bound of one arc; b) the guards of each of the arcs in E^a form a partition. The algorithm initializes F^a to $-\infty$ and then increases the

components of F^a iteratively. The computed components are stored in the auxiliary variable H^a. The set of guards associated with the arc in row k is denoted G_k, i.e., if $E^a_k[e] \neq 0$ then $G_k = \varphi(e)$. If a given linear program is infeasible (meaning that the guard it represents cannot be reached), then it is discarded. The subtraction of two elements f and f' in $F^a - F'^a$ is defined as 0 if both elements are $-\infty$, and as ∞ if f is finite and f' is $-\infty$.

Input : $\mathcal{N}_G, \theta, J_m, K_m, J_\lambda, K_\lambda, E_c, F_c, E, F$
Output : F^a
$F^a_k = -\infty$ for every row k in F^a
repeat
  $F'^a = F^a$
  for every row k in E^a do

$$
\begin{aligned}
H^a_k = \max\{F^a_k, \max_{\mathcal{R}_r \in G_k} \{\max\{E_k\Delta\lambda | \\
& m = \mu_P + Y_m\mu_E;\ C\Delta\lambda_U \leq D\mu_E; \lambda = \lambda_0 + Z_\lambda\Delta\lambda \\
& \Delta\lambda[e] = \Delta\lambda_U[(e,r)]\ \ \forall\ e \in E^T_S \text{ such that } \mathcal{R}_r \in \varphi(e) \\
& \sigma = a_T + Y_\sigma a_E;\ A\Delta m \leq Ba_E \\
& m = m_0 + Z_m\Delta m;\ J_m m_0 \leq K_m \\
& E_c\Delta\sigma = \theta F_c \\
& E^u\Delta\sigma \leq \theta F^u \\
& E^a_l\Delta\sigma \leq \theta F^a_l \text{ for every } l \text{ such that } F^a_l > -\infty \\
& E^a_m\Delta\sigma = 0 \text{ for every } m \text{ such that } F^a_m = -\infty \\
& \sigma = \lambda_0\theta + Z_\lambda\Delta\sigma; J_\lambda\lambda_0 \leq K_\lambda \\
& S_r\boldsymbol{x} \leq Q_r \\
& \mu_P[p_i] = 0\ \ \forall\ p_i \in P_F\}\}\}
\end{aligned}
$$

  end for
  $F^a = H^a$
until $\|F^a - F'^a\| \leq \epsilon$

Algorithm 1: Algorithm to compute intensity bounds.

The equality $E^a_m\Delta\sigma = 0$ ensures that the arcs for which a bound has not been computed yet, do not produce actions. Notice that while $\mu_P[p_i] = 0$ can be used to force the activity of tokes, the execution of actions must not be forced as we desire to compute bounds for the whole interval $[0, \theta]$. With respect to the above assumptions, if a) could be violated there might be linear relationships (determined by $C\Delta\lambda_U \leq D\mu_E$) between two arcs in E_k, and the equality $E^a_m\Delta\sigma = 0$ might avoid the computation of bounds for row k. Assumption b) guarantees that all the intensities of the state space are accounted for, even those that are equal to 0.

Notice that if the arcs in E^a_k are unguarded, then Algorithm 1 becomes simpler as G_k has only region that contains all the state space and $S_r\boldsymbol{x} \leq Q_r$ is trivially satisfied. Additional constraints, i.e., on the marking or on the

intensities, can be added to the algorithm if they are a requirement of the modeler.

Algorithm 1 can be extended to deal with intermediate states, see Section 5. Following the notation in Section 5, $\Omega = (\theta_0, \ldots, \theta_j)$ is a sequence of strictly increasing time instants, $SN = \{\mathcal{N}_G^1, \ldots, \mathcal{N}_G^j\}$ is a set of guarded flexible nets, and SJK and STR are defined as in Proposition 10. The objects corresponding to the time interval i are identified with the superscript i, e.g., F^i correspond to matrix F for the time interval i.

Algorithm 2 computes F^j for $j > 1$ (F^1 can be computed by Algorithm 1) by making use of the state equations and the bounds computed for the previous intervals. As in Section 5, only the marking is transferred from a given interval to the next one by means of the matrices in STR.

The bounds in Algorithm 2 could be further improved by substituting the constraints in each interval i such that $i < j$ by $TC_{\mathcal{N}_G^i}$ as in (89). Although this might lead to better results, the computational cost might be high.

Input : $\Omega, SN, SJK, STR, J_m, K_m, E_c^1, .., E_c^j, F_c^1, .., F_c^j, E^1, .., E^j, F^1, .., F^j$

Output : F^{aj}

$F_k^{aj} = -\infty$ for every row k in F^{aj}

repeat

$F'^{aj} = F^{aj}$

for every row k in E^{aj} do

$$
\begin{aligned}
H_k^{aj} = \max\{F_k^{aj}, \max_{\mathcal{R}_r^j \in G_k^j} \{\max\{E_k^{aj}\Delta\lambda^j |\\
&\sigma^1 = a_T^1 + Y_\sigma^1 a_E^1;\ A^1 \Delta m^1 \le B^1 a_E^1\\
&m^1 = m_0 + Z_m^1 \Delta m^1;\ J_m m_0 \le K_m\\
&E_c^1 \Delta\sigma^1 = (\theta_1 - \theta_0) F_c^1\\
&E^1 \Delta\sigma^1 \le (\theta_1 - \theta_0) F^1;\ \sigma^1 = \lambda_0^1(\theta_1 - \theta_0) + Z_\lambda^1 \Delta\sigma^1\\
&J_\lambda^1 \lambda_0^1 \le K_\lambda^1\\
&In_m^2 m_0^2 \le Out_m^1 m^1;\ In_a^2 a_0^2 \le Out_a^1 a_T^1\\
&\sigma^2 + a_0^2 = a_T^2 + Y_\sigma^2 a_E^2;\ A^2 \Delta m^2 \le B^2 a_E^2\\
&m^2 = m_0^2 + Z_m^2 \Delta m^2\\
&E_c^2 \Delta\sigma^2 = (\theta_2 - \theta_1) F_c^2\\
&E^2 \Delta\sigma^2 \le (\theta_2 - \theta_1) F^2;\ \sigma^2 = \lambda_0^2(\theta_2 - \theta_1) + Z_\lambda^2 \Delta\sigma^2\\
&J_\lambda^2 \lambda_0^2 \le K_\lambda^2\\
&In_m^3 m_0^3 \le Out_m^2 m^2;\ In_a^3 a_0^3 \le Out_a^2 a_T^2\\
&\cdots\\
&In_m^j m_0^j \le Out_m^{j-1} m^{j-1};\ In_a^j a_0^j \le Out_a^{j-1} a_T^{j-1}\\
&m^j = \mu_P^j + Y_m^j \mu_E^j;\ C^j \Delta\lambda_U^j \le D^j \mu_E^j; \lambda^j = \lambda_0^j + Z_\lambda^j \Delta\lambda^j\\
&\Delta\lambda^j[e] = \Delta\lambda_U^j[(e, r)] \quad \forall\ e \in E_S^T \text{ such that } \mathcal{R}_r \in \varphi(e)\\
&\sigma^j + a_0^j = a_T^j + Y_\sigma^j a_E^j;\ A^j \Delta m^j \le B^j a_E^j\\
&m^j = m_0^j + Z_m^j \Delta m^j\\
&E_c^j \Delta\sigma^j = (\theta_j - \theta_{j-1}) F_c^j\\
&E^{uj} \Delta\sigma^j \le (\theta_j - \theta_{j-1}) F^{uj}\\
&E_l^{aj} \Delta\sigma^j \le (\theta_j - \theta_{j-1}) F_l^{aj} \text{ for every } l \text{ such that } F_l^{aj} > -\infty\\
&E_m^{aj} \Delta\sigma = 0 \text{ for every } m \text{ such that } F_m^{aj} = -\infty\\
&\sigma^j = \lambda_0^j(\theta_j - \theta_{j-1}) + Z_\lambda^j \Delta\sigma^j;\ J_\lambda^j \lambda_0^j \le K_\lambda^j\\
&S_r^j \boldsymbol{x}^j \le Q_r^j\\
&\mu_P^j[p_i] = 0 \quad \forall\ p_i \in P_F^j\}\}\}
\end{aligned}
$$

end for

$F^{aj} = H^{aj}$

until $\|F^{aj} - F'^{aj}\| \le \epsilon$

Algorithm 2: Algorithm to compute intensity bounds for the time interval j.

B Relaxing non linear constraints

B.1 Linear approximations

The equations $\delta\Delta\lambda_U$ in (52) and (85), $\alpha_r\Delta\bar{\lambda}_{Ur}[(e,r)]$ and $\bar{\delta}\Delta\bar{\lambda}_G$ in (85), and $\bar{\delta}_r\bar{\boldsymbol{x}}_r$ in (85) are not linear and hence could be difficult to handle by a solver. Such constraints can be relaxed and approximated by inequalities containing real and binary variables [2]. Notice that while $\delta\Delta\lambda_U$ and $\alpha_r\Delta\bar{\lambda}_{Ur}[(e,r)]$ involve the multiplication of a binary variable and a real variable, $\bar{\delta}\Delta\bar{\lambda}_G$ and $\bar{\delta}_r\bar{\boldsymbol{x}}_r$ involve the multiplication of two real variables, one of them in the interval $[0,1]$.

The product $y=\alpha w$ where $\alpha\in\{0,1\}$ and $w\in[w_l,w_u]$ can be linearized as:

$$\begin{aligned} y &\leq \alpha w_u \\ y &\geq \alpha w_l \\ y &\leq w - w_l(1-\alpha) \\ y &\geq w - w_u(1-\alpha) \end{aligned} \tag{90}$$

This linearization can be used directly in the multiplications containing binary variables and real variables. The values of w_l and w_u do not need to be tight, and they can be computed by using the methods in Appendix C. As only $\Delta\lambda_U$, $\Delta\bar{\lambda}_U$ and $\Delta\bar{\lambda}_{Ur}$ can get negative values, w_l in (90) can be taken as 0 for the multiplications not involving any of these three variables.

Let us now focus on equations of the form $v=\phi u$ where both ϕ and u are nonnegative real variables and $\phi\in[0,1]$. Let us partition $[0,1]$ in q intervals $[0,d_1]$, $[d_1,d_2]$, ..., $[d_{q-1},1]$ and let us define $q{-}1$ binary variables $\beta_j\in\{0,1\}$ as follows:

$$\beta_j = 1 \;\leftrightarrow\; \phi \geq d_j \quad \forall j\in\{1,q{-}1\} \tag{91}$$

As in (67), such a necessary and sufficient condition can be modeled by including in the programming problem the following inequalities:

$$d_j - \phi \leq 2(1-\beta_j) \quad \forall j\in\{1,q{-}1\} \tag{92}$$

$$d_j - \phi \geq -2\beta_j \quad \forall j\in\{1,q{-}1\} \tag{93}$$

where $-2(2)$ is a lower(upper) bound for $d_j{-}\phi$, and (92) encodes the implication $(\phi<d_j\rightarrow\beta_j=0)$ and (93) encodes $(\phi>d_j\rightarrow\beta_j=1)$. The fact that β_j can be either 0 or 1 when $\phi=d_j$ does not pose a problem for the proposed linearization.

This way, the product $v=\phi u$, where $\phi\in[0,1]$ and $u\geq 0$, can be replaced and bounded by the following inequalities:

$$\left(\sum_{j=1}^{q-1}\beta_j(d_j-d_{j-1})\right)u \leq v \leq \left(1-\sum_{j=1}^{q-1}(1-\beta_j)(d_{j+1}-d_j)\right)u \quad \forall j\in\{1,q{-}1\} \tag{94}$$

where d_0 and d_q are defined as $d_0=0$ and $d_q=1$.

Remark that $\Delta\bar{\lambda}_{Ur}$ can be negative and hence the above approach cannot be used to compute the multiplication $\bar{\delta}_r\bar{\boldsymbol{x}}_r$ corresponding to the components $\Delta\bar{\lambda}_{Ur}$. However, as the systems dynamics is driven by $\Delta\bar{\lambda}_{Ur}$ (and in turn by $\Delta\bar{\lambda}_G$) and not by $\Delta\bar{\lambda}_U=\sum_{\mathcal{R}_r\in\mathcal{P}_n}\bar{\delta}_r\Delta\bar{\lambda}_{Ur}$, such multiplications corresponding to

$\Delta\bar{\lambda}_{Ur}$ can be ignored. Similarly, the components $\Delta\bar{\lambda}_r$ and $\bar{\lambda}_r$ in $\bar{\delta}_r\bar{\boldsymbol{x}}_r$ do not play a role in the system dynamics either, and consequently their multiplications can also be ignored.

In order to avoid that $\phi = d_j$ can occur for several ϕ and d_j (this can imply a loss of accuracy because β_j can be either 0 or 1) uneven intervals $[d_{j-1}, d_j]$ can be considered. Moreover, in order to minimize the error induced by the linearization in (94), it is advisable to partition the interval $[0, 1]$ densely near the most likely values of ϕ. For instance, in (85), $\bar{\delta}_r \in [0, 1]$ is the time ratio during which the system is in region $\mathcal{R}_r$. If it is assumed that all regions are equally likely, then the expected value of $\bar{\delta}_r$ is $1/n$ where $n = |\mathcal{P}_n|$ is the number of regions in partition $\mathcal{P}_n$. Thus, $\bar{\delta}_r$ can be approximated as a random variable with support $[0, 1]$ and mean $1/n$. If no more information about $\bar{\delta}_r$ is known, an appropriate density function is a truncated exponential as it is the one with maximum entropy. The density function would be $p(x) = he^{hx}/(e^h - 1)$ with h being the unique solution to $1/n = e^h/(e^h - 1) - 1/h$. The quantiles of such probability distribution can be used to set the values $d_1, d_2, \ldots$ of the intervals. In order to compute quantiles straightforwardly, the proposed truncated exponential function can be approximated by an exponential distribution with mean $1/n$ for $n > 2$, if $n = 2$ it becomes a uniform distribution.

Equation (94) still contains products of binary variables with real variables. Such products can be linearized by using the inequalities in (90).

The number of binary variables in (85) and (89) after applying the proposed relaxations is proportional to the number of regions and the number of intervals used to linearize products of variables. As discussed in Subsections 3.4 and 6.2, the number of regions can be decreased by merging some of them.

B.2 Average variables

The relaxations of $\bar{\delta}_r\bar{\boldsymbol{x}}_r$ in (85) can be improved in the following cases: a) a given variable $\bar{\boldsymbol{x}}_r$ is known to be 0; b) a given region $\mathcal{R}_r$ is not visited and hence $\bar{\delta}_r$ is 0; c) only one region $\mathcal{R}_r$ of a given partition is visited.

Case a): Variables $\bar{a}_T$, $\bar{a}_{T\tau}$ and $\bar{\mu}_P$ are 0 for those transitions that are required to be executed instantaneously, and those places whose tokens must always be active. Thus, equations

$$\bar{\mu}_{Pr}[p_i] = 0 \;\; \forall\, p_i \in P_{Av}, \;\forall\, \mathcal{R}_r \in \mathcal{R}$$

$$\bar{a}_{Tr}[t_j] = 0 \;\forall\, t_j \in T_{Av}, \;\forall\, \mathcal{R}_r \in \mathcal{R}$$

can be added to (85).

Case b): If $\mathcal{R}_r$ is not visited then α_r is 0. Hence the constraint:

$$\bar{\delta}_r\bar{\boldsymbol{x}}_r \leq \alpha_r w_u \;\; \forall\, \mathcal{R}_r \in \mathcal{R} \tag{95}$$

can be used to improve the product $\bar{\delta}_r\bar{\boldsymbol{x}}_r$ for every nonnegative $\bar{\boldsymbol{x}}_r$, i.e., $\Delta\bar{\lambda}_{Ur}$ is excluded from $\bar{\delta}_r\bar{\boldsymbol{x}}_r$, where w_u is an upper bound for $\boldsymbol{x}_r$.

Case c): If only one region $\mathcal{R}_r$ of a given partition is visited, then $\bar{\boldsymbol{x}}$ should be equal to $\bar{\boldsymbol{x}}_r$, and $\Delta\bar{\lambda}[e]$ should be equal to $\Delta\bar{\lambda}_G[(e, r)]$. This can be achieved by defining an auxiliary binary variable γ_r such that $\gamma_r = 1$ iff $\mathcal{R}_r$ is the only region visited of its partition. The vale of γ_r can be computed by the following

constraints:

$$1 - \epsilon \leq \bar{\delta}_r + 2(1 - \gamma_r) \;\; \forall\, \mathcal{R}_r \in \mathcal{R} \tag{96}$$

$$1 - \epsilon \geq \bar{\delta}_r - 2\gamma_r \;\; \forall\, \mathcal{R}_r \in \mathcal{R} \tag{97}$$

In order to avoid numerical issues, $\bar{\delta}_r$ can be set exactly to 0 for those regions that are not visited with the following constraint:

$$\bar{\delta}_r \leq \alpha_r \;\; \forall\, \mathcal{R}_r \in \mathcal{R} \tag{98}$$

Then, the constraints below can be added to set $\bar{\boldsymbol{x}} = \bar{\boldsymbol{x}}_r$ where $\mathcal{R}_r$ is the only visited region of its partition:

$$\bar{\boldsymbol{x}} \leq \bar{\boldsymbol{x}}_r + w_u(1 - \gamma_r) \quad \forall\, \mathcal{R}_r \in \mathcal{P}_n \tag{99}$$

$$\bar{\boldsymbol{x}} \geq \bar{\boldsymbol{x}}_r - 2w_u(1 - \gamma_r) \quad \forall\, \mathcal{R}_r \in \mathcal{P}_n \tag{100}$$

where w_u is an upper bound for $\boldsymbol{x}$.

Similarly, $\Delta\bar{\lambda}[e] = \Delta\bar{\lambda}_G[(e, r)]$ can be obtained by:

$$\Delta\bar{\lambda}[e] \leq \Delta\bar{\lambda}_G[(e, r)] + w_u(1 - \gamma_r) \quad \forall\, e \in E_S^T,\; \forall\, \mathcal{R}_r \in \varphi(e) \tag{101}$$

$$\Delta\bar{\lambda}[e] \geq \Delta\bar{\lambda}_G[(e, r)] - 2w_u(1 - \gamma_r) \quad \forall\, e \in E_S^T,\; \forall\, \mathcal{R}_r \in \varphi(e) \tag{102}$$

where w_u is an upper bound for $\Delta\lambda$.

C Auxiliary bounds

This section proposes methods to compute values for W, and for the auxiliary bounds w_l and w_u to linearize products of variables. More precisely, the following values will be computed for each type of analysis:

1. Untimed: bounds to linearize $\delta\Delta\lambda_U$ and components of W in (52).
2. Timed: bounds to linearize $\delta\Delta\lambda_U$, $\bar{\delta}\Delta\bar{\lambda}_G$, $\bar{\delta}_r\bar{\boldsymbol{x}}_r$, $\alpha_r\Delta\bar{\lambda}_{Ur}[(e, r)]$ and components of W in (85).

For simplicity, it will be assumed that $\Delta\lambda_U$ is bounded by the constraint $C\Delta\lambda_U \leq D\mu_E$.

C.1 Untimed

Let us consider the following set of constraints based on (45):

$$\begin{aligned} C_{un} = \{ & m = \mu_P + Y_m\mu_E;\; C\Delta\lambda_U \leq D\mu_E \\ & \sigma = a_T + Y_\sigma a_E;\; A\Delta m \leq Ba_E \\ & m = m_0 + Z_m\Delta m;\; J_m m_0 \leq K_m \} \end{aligned} \tag{103}$$

Values for w_l, w_u and W can be computed by combining C_{un} with appropriate objective functions. As in Appendix A, additional constraints can be added to (103) if they are a requirement that must be satisfied by the model.

Namely, w_l and w_u for the linearization of $\delta\Delta\lambda_U$ in (52) can be obtained from the following optimization problems:

$$\begin{aligned} w_l &= \min_{\substack{(e,r)\\ e\in E_S^T, \mathcal{R}_r\in\varphi(e)}} \{\min\{\Delta\lambda_U[(e,r)|C_{un}\}\} \\ w_u &= \max_{\substack{(e,r)\\ e\in E_S^T, \mathcal{R}_r\in\varphi(e)}} \{\max\{\Delta\lambda_U[(e,r)|C_{un}\}\} \end{aligned} \tag{104}$$

On the other hand, the value w produced by the linear program (105) can be used for all the components of W in (52).

$$w = \max\{\sum_{\mathcal{R}_r\in\mathcal{R}} \sum_k \big(\sum_l |S_r[k,l]|\boldsymbol{x}[l] + |Q_r[k]|\big)|C_{un}\} \tag{105}$$

where k and l are the indices for the rows and columns of S_r. Notice that in this case, the constraint $C\Delta\lambda_U \leq D\mu_E$ in C_{un} can be removed as the columns of S_r that correspond to $\Delta\lambda_U$, $\Delta\lambda$ and λ are assumed to be 0.

C.2 Timed

If the problems (104) and (105) are unbounded, then time dependent values for w_l, w_u and W could still be computed for the time interval $[0,\theta]$. Consider the following set of constraints:

$$\begin{aligned} C_{tr} = \{&m = \mu_P + Y_m\mu_E;\ C\Delta\lambda_U \leq D\mu_E \\ &\sigma = a_T + Y_\sigma a_E;\ A\Delta m \leq Ba_E \\ &m = m_0 + Z_m\Delta m;\ J_m m_0 \leq K_m \\ &E_c\Delta\sigma = \theta F_c \\ &E\Delta\sigma \leq \theta F;\ \sigma = \lambda_0\theta + Z_\lambda\Delta\sigma;\ \ J_\lambda\lambda_0 \leq K_\lambda\} \end{aligned} \tag{106}$$

The following problems produce values for w_l and w_u for the linearization of $\delta\Delta\lambda_U$ in (85), $\bar{\delta}\Delta\bar{\lambda}_G$ in (85) and $\alpha_r\Delta\bar{\lambda}_{Ur}[(e,r)]$.

$$\begin{aligned} w_l &= \min_{\substack{(e,r)\\ e\in E_S^T, \mathcal{R}_r\in\varphi(e)}} \{\min\{\Delta\lambda_U[(e,r)|C_{tr}\}\} \\ w_u &= \max_{\substack{(e,r)\\ e\in E_S^T, \mathcal{R}_r\in\varphi(e)}} \{\max\{\Delta\lambda_U[(e,r)|C_{tr}\}\} \end{aligned} \tag{107}$$

As discussed in Appendix B, it is assumed that the components of the intensity variables $\Delta\bar{\lambda}_{Ur}$, $\Delta\bar{\lambda}_r$ and $\bar{\lambda}_r$ in the multiplication $\bar{\delta}_r\bar{\boldsymbol{x}}_r$ in (85) are ignored. Thus, we will focus on $\bar{\delta}_r\bar{\boldsymbol{x}}'_r$, where $\bar{\boldsymbol{x}}'_r$ is the result of removing the components $\Delta\bar{\lambda}_{Ur}$, $\Delta\bar{\lambda}_r$ and $\bar{\lambda}_r$ from $\bar{\boldsymbol{x}}_r$. The value for w'_u for the linearization of $\bar{\delta}_r\bar{\boldsymbol{x}}'_r$ can be obtained from:

$$w'_u = \max\{\mathbf{1}m + \mathbf{1}\Delta\sigma + \mathbf{1}\sigma + \mathbf{1}\Delta m|C_{tr}\} \tag{108}$$

In the objective function, the terms $\mathbf{1}m$; $\mathbf{1}\Delta\sigma$; $\mathbf{1}\sigma$; $\mathbf{1}\Delta m$ guarantee and upper bound for $\bar{m}$, $\bar{\mu}_P$ and $\bar{\mu}_E$; $\Delta\bar{\sigma}$; $\bar{\sigma}$, $\bar{a}_T$ and $\bar{a}_E$; and $\Delta\bar{m}$ respectively. Notice that, as all the variables in $\boldsymbol{x}'$ are nonnegative, 0 is a trivial value for w'_l for the linearization of $\bar{\delta}_r\bar{\boldsymbol{x}}'_r$.

All the components of W in (85) can be set to the value w given by:

$$w = \max\{ \sum_{\mathcal{R}_r \in \mathcal{R}} \sum_{k} \big(\sum_{l} |S_r[k,l]| \boldsymbol{x}[l] + |Q_r[k]| \big) |C_{tr}\} \tag{109}$$

In order to account for intermediate states, the set of constraints in (110) is proposed. Similar optimization problems to the ones introduced above can be designed on the basis of these constraints to compute auxiliary bounds.

$$\begin{aligned}
C_{istr} = \{ & \sigma^1 = a_T^1 + Y_\sigma^1 a_E^1;\ A^1 \Delta m^1 \leq B^1 a_E^1 \\
& m^1 = m_0 + Z_m^1 \Delta m^1;\ J_m m_0 \leq K_m \\
& E_c^1 \Delta\sigma^1 = (\theta_1 - \theta_0) F_c^1 \\
& E^1 \Delta\sigma^1 \leq (\theta_1 - \theta_0) F^1;\ \sigma^1 = \lambda_0^1 (\theta_1 - \theta_0) + Z_\lambda^1 \Delta\sigma^1 \\
& J_\lambda^1 \lambda_0^1 \leq K_\lambda^1 \\
& In_m^2 m_0^2 \leq Out_m^1 m^1;\ In_a^2 a_0^2 \leq Out_a^1 a_T^1 \\
& \sigma^2 + a_0^2 = a_T^2 + Y_\sigma^2 a_E^2;\ A^2 \Delta m^2 \leq B^2 a_E^2 \\
& m^2 = m_0^2 + Z_m^2 \Delta m^2 \\
& E_c^2 \Delta\sigma^2 = (\theta_2 - \theta_1) F_c^2 \\
& E^2 \Delta\sigma^2 \leq (\theta_2 - \theta_1) F^2;\ \sigma^2 = \lambda_0^2 (\theta_2 - \theta_1) + Z_\lambda^2 \Delta\sigma^2 \\
& J_\lambda^2 \lambda_0^2 \leq K_\lambda^2 \\
& In_m^3 m_0^3 \leq Out_m^2 m^2;\ In_a^3 a_0^3 \leq Out_a^2 a_T^2 \\
& \dots \\
& In_m^j m_0^j \leq Out_m^{j-1} m^{j-1};\ In_a^j a_0^j \leq Out_a^{j-1} a_T^{j-1} \\
& m = \mu_P + Y_m^j \mu_E;\ C^j \Delta\lambda_U \leq D^j \mu_E \\
& \sigma + a_0^j = a_T + Y_\sigma^j a_E;\ A^j \Delta m \leq B^j a_E \\
& m = m_0^j + Z_m^j \Delta m \\
& E_c^j \Delta\sigma = (\theta_j - \theta_{j-1}) F_c^j \\
& E^j \Delta\sigma \leq (\theta_j - \theta_{j-1}) F^j;\ \sigma = \lambda_0 (\theta_j - \theta_{j-1}) + Z_\lambda^j \Delta\sigma \\
& J_\lambda^j \lambda_0 \leq K_\lambda^j \}
\end{aligned} \tag{110}$$

References

[1] M. Ajmone Marsan, G. Balbo, G. Conte, S. Donatelli, and G. Franceschinis. *Modelling with Generalized Stochastic Petri Nets.* Wiley, 1995.

[2] A. Bemporad and M. Morari. Control of systems integrating logic, dynamics, and constraints. *Automatica*, 35(3):407–427, March 1999.

[3] B. Kouvaritakis and M. Cannon. *Model Predictive Control. Classical, Robust and Stochastic.* Springer International Publishing, 2016.

[4] T. Murata. Petri Nets: Properties, Analysis and Applications. *Procs. of the IEEE*, 77(4):541–580, 1989.

[5] M. Silva, E. Teruel, and J. M. Colom. Linear Algebraic and Linear Programming Techniques for the Analysis of Net Systems. *Lecture Notes in Computer Science*, 1491:309–373, 1998.
