# Supplementary material for: Handling variability and incompleteness of biological data by flexible nets: a case study for Wilson disease
Source: NPJ Syst Biol Appl. 2018 Jan 11;4:7. doi: 10.1038/s41540-017-0044-x (PMC5765040; doi:10.1038/s41540-017-0044-x)
Supplement: Supplementary file 2 — Supplemental File S2 [file 41540_2017_44_MOESM2_ESM.pdf]

Supplementary Information

FN modeling the glucose consumption by yeast population

1 Model description

The glucose consumption system for an individual yeast cell was modeled by the FN in Fig 1. The state of the system is given by the extracellular and intracellular glucose concentrations, $[Go]$ and $[Gi]$ respectively, which are modeled by the places Go and Gi. Three processes that change the state were considered: the inflow of extracellular glucose (this is a control variable in the considered control problems), the glucose uptake across the cell boundary, and the glucose utilization; these processes are modeled by transitions F, HXT and R, respectively. The net has one event handler per transition, vF, $vHXT$ and vR. The equations associated with the event handlers are vF:f=go, $vHXT$:hxt=go=gi and vR:r=gi. The net has one intensity handler, $sHXT$, that models how $[Gi]$ and $[Go]$ determine the glucose uptake rate. Details on how this rate is modeled and default intensities of transitions are provided in the next section.

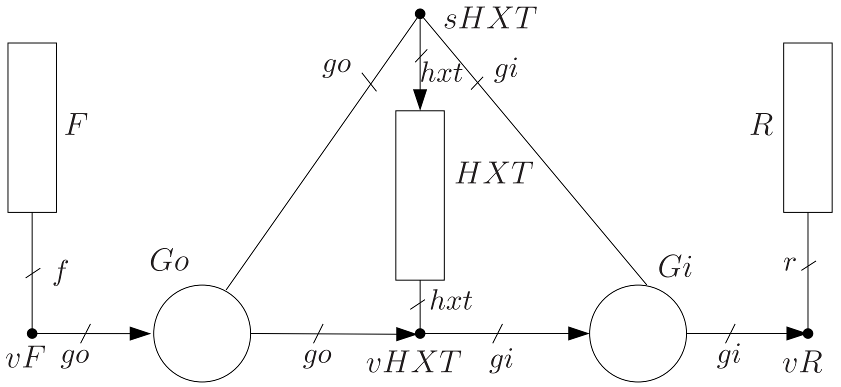

Figure 1: **FN modeling glucose consumption.**

Growth was represented by the doubling of cell mass, which is abstracted by the accumulation of intracellular glucose until its amount reaches twice the initial value. The model omits cell death during the described exponential growth period of the population as an assumption. The fact that the division of the daughter yeast cell is slower than that of its mother was taken into account by the introduction of a unitless parameter h as 5.804e-2 and 2.107e-1 to adjust for the time it takes to accumulate the targeted amount of

glucose to match the reported doubling times for yeast cells during low and high affinity periods, respectively. These parameters allowed the system to accumulate glucose within the first 25-35 minutes of each doubling period, corresponding approximately to the G1 phase, remaining constant thereafter for the duration of S-G2-M phases of the cell cycle [1].

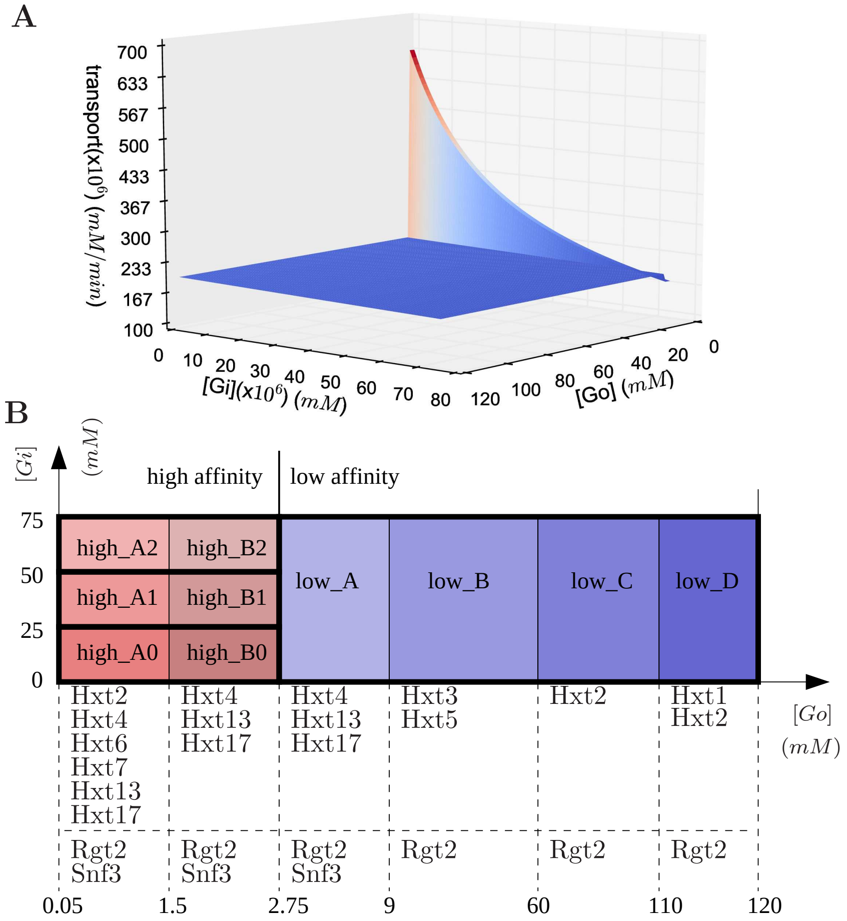

Figure 2: **Glucose uptake rate.** A: Glucose uptake rate (1) as a function of intracellular and extracellular glucose in a wild type yeast cell. The flat surface and the narrow band with high rates correspond to the low and high affinity modes, respectively. B: Activity regions of the yeast glucose sensors (Rgt2 and Snf3) and the yeast hexose transporters (Hxt1-7, Hxt13, and Hxt17). The rectangular regions represent the *guards* in the net model. In both A and B, Go and Gi are in mM and the rate of transport (xe6) is in mM/min. Notice that the axes are not in scale.

2 System parameters

The initial concentrations of glucose are $m_0[Go] = 110\ mM$ and $m_0[Gi] = 2.1e\text{-}6\ mM$.

We adapted the glucose utilization rate to meet the glucose-equivalent energy required for maintenance from [7] as $199e\text{-}6\ mM/min$ normalized for a single yeast cell. This is modeled by setting the default intensity $\lambda_0[R]$ to $199e\text{-}6$. The default intensity of the control variable F was constrained to the interval $[0, 110e\text{-}6]$. As discussed below, the intensity of HXT depends on $[Go]$ and $[Gi]$, and hence, its default intensity is set to 0.

Glucose uptake across the cell boundary employs the relevant sensors [2] and transporters [3] (see Fig 2). The hexose transport rate is given by [5]:

$$transport = V_{comp} \left(\frac{V_{max} \left(\frac{[Go]}{K_m} - \frac{[Gi]}{K_m} \right)}{1 + \frac{[Go]}{K_m} + \frac{[Gi]}{K_m} + \frac{0.91 \cdot [Go] \cdot [Gi]}{K_m^2}} \right) \qquad (1)$$

where $[Go]$ and $[Gi]$ are the concentrations of extracellular and intracellular glucose (mM), respectively, V_{max} is the maximum specific growth rate (mM/min), K_m is the affinity constant (mM), and V_{comp} is the unit compartment volume (L). The ranges of $[Go]$ and $[Gi]$ under investigation are $5e\text{-}2\ mM \leq [Go] \leq 120\ mM$ and $0\ mM \leq [Gi] \leq 75e\text{-}6\ mM$. Affinity-dependent values of these constants were used whenever available, adjusted to a single yeast cell. If $[Go] \geq 2.75\ mM$ then the affinity is low [6] and $V_{max} = 3.44e\text{-}3\ mM/min$ and $K_m = 1.1655e\text{-}3\ mM$. If $[Go] < 2.75\ mM$ then the affinity is high and $V_{max} = 3.0e\text{-}3\ mM/min$ and $K_m = 2.50278e\text{-}5\ mM$ [4]. The units of $[Gi]$ and $[Go]$ were used consistently throughout our study, however, for the sake of presentation, $[Gi]$ and $[Go]$ can be expressed in either mM or $mmol$.

Fig 2A plots the transport rate given by (1) as a function of $[Gi]$ and $[Go]$. Given that the transport rate is nonlinear, it was approximated by a piece-wise linear function as follows. The state space was partitioned into regions, and each region was assumed to follow a linear dynamic profile given by the linear regression (ordinary least squares) of (1), i.e. the FN in Fig 1 is guarded. Since, the transport rate profile is rather flat in low affinity mode, relatively few regions were sufficient to approximate the rate, whereas more regions were required to approximate the rapidly changing high affinity mode. In particular, for analyses not involving genetic chages, the state space was partitioned into 4 regions (corresponding to the rectangles with bold boundaries in Fig 2B). For analyses involving genetic changes, the state

space was re-partitioned into 10 regions, each corresponding to a coloured rectangle in Fig 2B.

Fig 2B shows the activity regions of the yeast glucose sensors (Rgt2 and Snf3) and the yeast hexose transporters (Hxt1-7, Hxt13, and Hxt17) determined by Gi and Go. The affinity profile of the system is based on Go. If at least one sensor and one transporter is active then the transport rate is given by (1), otherwise the transport rate becomes 0.

References

[1] B. J. Brewer, E. Chlebowicz-Sledziewska, and W. L. Fangman. Cell cycle phases in the unequal mother/daughter cell cycles of Saccharomyces cerevisiae. *Molecular and Cellular Biology*, 4(11):2529–2531, 1984.

[2] S. Busti, P. Coccetti, L. Alberghina, and M. Vanoni. Glucose Signaling-Mediated Coordination of Cell Growth and Cell Cycle in Saccharomyces Cerevisiae. *Sensors*, 10(6):6195, 2010.

[3] S. Ozcan and M. Johnston. Function and regulation of yeast hexose transporters. *Microbiology and Molecular Biology Reviews*, 63(3):554–569, 1999.

[4] B. Teusink, J. A. Diderich, H. V. Westerhoff, K. van Dam, and M. C. Walsh. Intracellular glucose concentration in derepressed yeast cells consuming glucose is high enough to reduce the glucose transport rate by 50%. *Journal of Bacteriology*, 180(3):556–562, 1998.

[5] B. Teusink, J. Passarge, C. A. Reijenga, E. Esgalhado, C. C. van der Weijden, M. Schepper, M. C. Walsh, B. M. Bakker, K. van Dam, H. V. Westerhoff, and J. L. Snoep. Can yeast glycolysis be understood in terms of in vitro kinetics of the constituent enzymes? Testing biochemistry. *European Journal of Biochemistry*, 267(17):5313–5329, 2000.

[6] W. A. Wilson, S. A. Hawley, and D. Hardie. Glucose repression/derepression in budding yeast: SNF1 protein kinase is activated by phosphorylation under derepressing conditions, and this correlates with a high AMP:ATP ratio. *Current Biology*, 6(11):1426 – 1434, 1996.

[7] M. Zakhartsev, X. Yang, M. Reuss, and H. O. Pörtner. Metabolic efficiency in yeast Saccharomyces cerevisiae in relation to temperature dependent growth and biomass yield. *Journal of Thermal Biology*, 52:117 – 129, 2015.
