# Supplementary material for: Handling variability and incompleteness of biological data by flexible nets: a case study for Wilson disease
Source: NPJ Syst Biol Appl. 2018 Jan 11;4:7. doi: 10.1038/s41540-017-0044-x (PMC5765040; doi:10.1038/s41540-017-0044-x)
Supplement: Supplementary file 3 — Supplemental File S3 [file 41540_2017_44_MOESM3_ESM.pdf]

Supplementary Information

FN modeling the Wilson disease

1 Model description

The FN modeling the Wilson disease is shown in Fig 1. Transition t_CI represents the copper intake, at least 99% of the intake flow is directed towards p_CA and the remainder towards p_Cuin. Transition t_CR models the flow of copper taken from p_CuA to be used in different parts of the body. The places p_CuA, p_CuB, p_CuC and p_Cuproc were used to split copper flows, and hence, their marking is forced to be 0 throughout the performed analyses. The red elements model the additional dissipation of ACP, which does not occur in healthy individuals, and hence, these elements were not considered when studying the healthy state. The green net elements open a path from the copper pool, p_Cupool, and allow such copper to be excreted in the feces. Such green elements were only considered when a treatment, either copper absorption blocking or urinary excretion induction, was applied. The blue net elements allow the copper in the pool to be processed and used in the body, such a path is only open when the copper absorption blocking treatment in being applied. The dotted lines represent the liver boundaries.

A short description of the net elements together with the sets of inequalities associated with handlers can be found in Table 1. For short, the handlers with one to one relationships among the connected elements are not shown in Table 1, e.g., in event handler v_CFUE a one unit speed in t_CFUE produces a one unit flow from p_Cupool to p_CuU, we express this as $(t\_CFUE, v\_CFUE) = (p\_Cupool, v\_CFUE) = (v\_CFUE, p\_CuU)$, and this one to one relationship is ommitted in Table 1. The places p_CuA, p_CuB, p_CuC and p_Cuproc were used to split the copper flows and are also ommitted in Table 1.

2 System parameters

The model parameters, i.e., initial concentrations and flows, depend on the *case* or *state* under consideration. Here, we considered four different states: healthy (no copper accumulation), sick (copper accumulation dependent on ATP7b functionality), copper absorption blocking treatment and urinary excretion induction treatment. The constraints of the model are provided in Table 2 for the healthy and sick case, and in Table 3 for the treatment cases. We adjusted the constraints such that they complied with the AASLD Practice Guidelines for the Diagnosis of Wilson disease.

All values are reported for the hepatocyte system with a defined boundary, unless stated otherwise. The "healthy" term denotes the maximal allowable range for each parameter as reported in medical guidelines to describe the healthy state. The "sick" term denotes the minimal diagnostic thresholds for the relevant parameters. The "absorption blocker" term denotes the employment of intestinal copper absorption blockers or metallothionein inducers; Zinc or tetrathiomolybdate, and the "cuprupria inducer" term denotes the employment of the copper chelators; D-penicillamine or trientine, for the treatment of

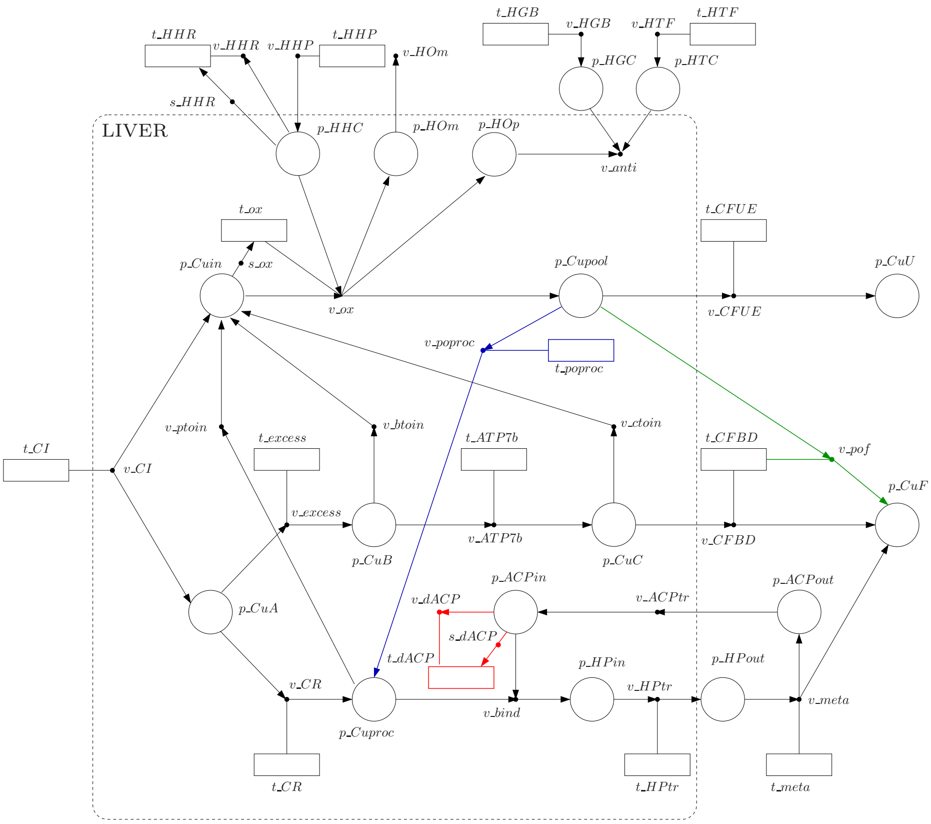

Figure 1: **FN model of the Wilson disease.**

Wilson disease and lifelong management of the condition. Two values or ranges separated by "/" in a column denote the value for the parameter during initial symptomatic treatment followed by the maintenance treatment in the given order. A standard weight for an adult human body was taken as $70kg$ whenever necessary.

Hepatocyte to liver ratios were adopted from [1]. The typical volume of a hepatocyte was adopted from [6]. In order to maintain the pseudo-first order state for the oxidation reaction involving Cu(I), the H_2O_2 concentration was ensured to be at least 5-fold higher than amount of Cu(I) entering the system boundary [11]. Mass stochiometries were handled via molecular weights.

References

[1] M. N. Berry and A. M. Edwards. *The Hepatocyte Review*. Springer, 2000.

[2] G. Brewer. Diagnosis of Wilson's disease: an experience over three decades. *Gut*, 50(1):136, January 2002.

[3] G. J. Brewer. Treatment of Wilson's disease: our patients deserve better. *Expert Opinion on Orphan Drugs*, 2(12):1245–1248, 2014.

[4] U. E.J. *Trace Elements in Humanand Animal Nutrition.* Academic Press Inc. New York., 1956.

[5] D. Frommer. Defective biliary excretion of copper in Wilson's disease. *Gut*, 2(15):125—129, 1974.

[6] L. H., B. A., Z. S. L., M. P., B. D., and D. J. E. *Molecular Cell Biology (Fifth Edition).* W. H. Freeman and Company. New York, 2000.

[7] D. Huster, A. Kühne, A. Bhattacharjee, L. Raines, V. Jantsch, J. Noe, W. Schirrmeister, I. Sommerer, O. Sabri, F. Berr, J. Mössner, B. Stieger, K. Caca, and S. Lutsenko. Diverse functional properties of Wilson disease {ATP7B} variants. *Gastroenterology*, 142(4):947 – 956.e5, 2012.

[8] N. Kaplowitz. The importance and regulation of hepatic glutathione. *The Yale Journal of Biology and Medicine*, 6(54):497—502, 1981.

[9] I. of Medicine (US) Panel on Micronutrients. *Dietary Reference Intakes for Vitamin A, Vitamin K, Arsenic, Boron, Chromium, Copper, Iodine, Iron, Manganese, Molybdenum, Nickel, Silicon, Vanadium, and Zinc.* National Academies Press (US), 2001.

[10] J. E. Perdigão. *Tooth Whitening. An Evidence-Based Perspective.* Springer, 2016.

[11] K. Ranguelova, D. Ganini, M. G. Bonini, R. E. London, and R. P. Mason. Kinetics of the oxidation of reduced cu,zn-superoxide dismutase by peroxymonocarbonate. *Free radical biology & medicine*, 53(3):589—594, August 2012.

[12] E. A. Roberts and M. L. Schilsky. Diagnosis and treatment of Wilson disease: An update. *Hepatology*, 47(6):2089–2111, 2008.

[13] E. Rocchi, Y. Seium, L. Camellini, G. Casalgrandi, A. Borghi, P. D'Alimonte, and G. Cioni. Hepatic tocopherol content in primary hepatocellular carcinoma and liver metastases. *Hepatology*, 26(1):67 – 72, 1997.

[14] E. Sedlák, G. Zoldák, and P. Wittung-Stafshede. Role of copper in thermal stability of human ceruloplasmin. *Biophysical journal*, 94(4):1384—1391, February 2008.

[15] J. Williams. The decomposition of hydrogen peroxide by liver catalase. *The Journal of General Physiology*, 11(4):309–337, 1928.

[16] G. Wu, Y.-Z. Fang, S. Yang, J. R. Lupton, and N. D. Turner. Glutathione metabolism and its implications for health. *The Journal of Nutrition*, 134(3):489–492, 2004.

[17] V. Yuzbasiyan-Gurkan, A. Grider, T. Nostrant, R. Cousins, and G. Brewer. Treatment of Wilson's disease with zinc: X. Intestinal metallothionein induction. *The Journal of laboratory and clinical medicine*, 120(3):380—386, September 1992.

Table 1: Description of the net elements of the FN in Fig 1

Element	Description
t_CI	Copper intake
t_ox	Copper oxidation
t_HHP	H_2O_2 production
t_HHR	H_2O_2 decomposition
t_HGB	Hepatic glutathione biosynthesis
t_HTF	Hepatic tocopherol inflow
t_CFUE	Flow for urinary excretion
t_CR	Copper requirement
t_excess	Excess of copper
t_dACP	Additional dissipation of ACP
t_HPtr	Transport of CP
t_meta	Metabolic processes
t_ATP7b	ATP7b process
t_CFBD	Flow to biliary ducts
t_poproc	Flow to cover copper requirement
p_Cuin	Copper to be oxidised
p_HHC	H_2O_2
p_HOm	HO^-
p_HOp	$HO^\bullet$
p_HGC	Hepatic glutathione
p_HTC	Hepatic tocopherol
p_Cupool	Copper pool
p_HPin	CP in liver
p_HPout	CP outside liver
p_ACPin	ACP in liver
p_ACPout	ACP outside liver
p_CuU	Copper in urine
p_CuF	Copper in feces
v_CI	At most 1% of the copper intake is directed to p_Cuin:<br>$(v\_CI, p\_Cuin) <= 0.01(t\_CI, v\_CI)$,<br>$(v\_CI, p\_Cuin) + (v\_CI, p\_CuA) = (t\_CI, v\_CI)$
v_ox	The ratio copper to be oxidised to H_2O_2 is 2 : 1:<br>$(p\_Cuin, v\_ox) == 2(p\_HHC, v\_ox)$
v_anti	Antioxidation of $HO^\bullet$:<br>$(p\_HOp, v\_anti)/17 == (p\_HGC, v\_anti)/307$<br>$+(p\_HTC, v\_anti)/431$
v_bind	Bind copper to ACP:<br>$(v\_bind, p\_HPin) = (p\_Cuproc, v\_bind) + (p\_ACPin, v\_bind)$,<br>$(p\_Cuproc, v\_bind)/0.00315 = (p\_ACPin, v\_bind)$
v_meta	Unbind copper to ACP:<br>$(p\_HPout, v\_meta) = (v\_meta, p\_CuF) + (v\_meta, p\_ACPout)$,<br>$(v\_meta, p\_CuF)/0.00315 = (v\_meta, p\_ACPout)$,<br>$(t_{m}eta, v_{m}eta) = (v\_meta, p\_CuF)$
s_ox	Oxidation rate: $(s\_ox, t\_ox) = 2.924e5(p\_Cuin, s\_ox)$
s_HHR	H_2O_2 decomposition rate:<br>$(s\_HHR, t\_HHR) = 685.44(p\_HHC, s\_HHR)$
s_dACP	ACP degradation rate: $(s\_dACP, t\_dACP) =$<br>$0.5(1-ATP7b\_functionality)(p\_ACPin, s\_dACP)$

Table 2: System parameters for copper metabolism in healthy and sick states

Parameter	Healthy	Sick
ATP7b functionality (AF)	1 [7]	0.015-0.555 [7]
Cu intake range (CI) (mg/day)	1.2-10.0 [9]	1.2-10.0 [9]
Cu requirement (CR) (mg/day)	0.9±5% [12]	0.9±5% [12]
Cu flow to biliary ducts (CFBD) (mg/day)	1.18±5% [5]	0.619±5% [5]
Cu flow for urinary excretion (CFUE) (mg/day)	0.02-0.04 [12, 2]	0.1-0.3 [12]
Biliary Cu excretion: urinary Cu excretion (BCE:UCE)	$\geq$ 9 [4]	$\leq$ 9 [4, 12]
Total CP concentration (TCC) (mg)	400-800 [12]	$\leq$400 [12]
Cu-bound CP concentration (CCC) (mg)	200-400 [12]	$\leq$ 50 [12]
Ceruloplasmin-bound Cu : free Cu (CBC:FC)	$\geq$ 9 [12]	$\leq$ 0.63 [12]
1st order rate constant for additional ACP dissipation (k_{dacp}) (day^{-1})	0	0.5 [14]
Hepatic Cu content (HCC) (mg)	52.5±5% [12],	$\geq$ 52.5±5% [12]
Hepatic H_2O_2 production (HHP) (mg/day)	6480±5% [10, 11]	6480±5% [10, 11]
1st order rate constant for hepatic H_2O_2 decomposition ($k_{H_2O_2}$) (day^{-1})	685.44 [15]	685.44 [15]
Hepatic glutathione biosynthesis (HGB) (mg/day)	$\leq$1547 [16]	$\leq$1547 [16]
Hepatic glutathione concentration (HGC) (mg)	$\leq$ 2149 [8]	$\leq$ 2149 [8]
1st order rate constant for Cu oxidation (k_{CuO}) (day^{-1})	$2.924e5$ [11]	$2.924e5$ [11]
Hepatic tocopherol content (HTC) (mg)	$\leq$ 290 [13]	$\leq$161 [13]

Table 3: System parameters for copper metabolism under treatment

Parameter	Absorption blocker	Cupruria inducer
ATP7b functionality (AF)	0.015-0.555 [7, 3]	0.015-0.555 [7]
Cu intake range (CI) (mg/day)	0.0-0.9 [9]	1.0-2.0 [9]
Cu requirement (CR) (mg/day)	0.9±5% [12]	0.9±5% [12]
Cu flow to biliary ducts (CFBD) (mg/day)	0.619-1.18 [5, 17]	0.619±5% [5]
Cu flow for urinary excretion (CFUE) (mg/day)	$\leq$0.1/0.03-0.08 [12]	0.5-1.0 / 0.2-0.5 [12]
Biliary Cu excretion: urinary Cu excretion (BCE:UCE)	$\leq$ 9 [4, 12]	$\leq$ 9 [4, 12]
Total CP concentration (TCC) (mg)	$\leq$400 [12]	$\leq$400 [12]
Cu-bound CP concentration (CCC) (mg)	$\leq$ 50 [12]	$\leq$ 50 [12]
Ceruloplasmin-bound Cu : free Cu (CBC:FC)	$\leq$ 0.63/0.63-1.05 [12]	$\leq$ 0.63/0.63-1.05 [12]
1st order rate constant for additional ACP dissipation (k_{dacp}) (day^{-1})	0.5 [14]	0.5 [14]
Hepatic Cu content (HCC) (mg)	52.5±5% [12],	52.5±5% [12]
Hepatic H_2O_2 production (HHP) (mg/day)	6480±5% [10, 11]	6480±5% [10, 11]
1st order rate constant for hepatic H_2O_2 decomposition ($k_{H_2O_2}$) (day^{-1})	685.44 [15]	685.44 [15]
Hepatic glutathione biosynthesis (HGB) (mg/day)	$\leq$1547 [16]	$\leq$1547 [16]
Hepatic glutathione concentration (HGC) (mg)	$\leq$ 2149 [8]	$\leq$ 2149 [8]
1st order rate constant for Cu oxidation (k_{CuO}) (day^{-1})	$2.924e5$ [11]	$2.924e5$ [11]
Hepatic tocopherol content (HTC) (mg)	$\leq$ 161 [13]	$\leq$ 161 [13]
Target hepatic tocopherol content (THTC) (mg)	$\leq$ 290, $\geq HTC$ [13]	$\leq$ 290, $\geq HTC$ [13]
